# Navigating Uncertainty in Clinical Practice: A Workshop to Prepare Medical Students to Problem-Solve During Complex Clinical Challenges

**DOI:** 10.15766/mep_2374-8265.11334

**Published:** 2023-08-09

**Authors:** Frances Rusnack, Dimitrios Papanagnou, Kestrel Reopelle, Dylan Devlin, Jared Kilpatrick, Martinique Ogle, Mary Stephens, Deborah Ziring, Nethra Ankam

**Affiliations:** 1 Clinical Instructor, Department of Emergency Medicine, Sidney Kimmel Medical College at Thomas Jefferson University; 2 Professor and Vice Chair for Education, Department of Emergency Medicine, and Associate Dean for Faculty Development, Sidney Kimmel Medical College at Thomas Jefferson University; 3 Clinical Instructor, Department of Emergency Medicine, Yale School of Medicine; 4 Assistant Professor, Department of Emergency Medicine, Course Director of Clinical Skills, and Course Director of Introductory Clinical Experience, Western Michigan University Homer Stryker M.D. School of Medicine; 5 Second-Year Medical Student, Sidney Kimmel Medical College at Thomas Jefferson University; 6 Associate Professor, Department of Family and Community Medicine, Sidney Kimmel Medical College at Thomas Jefferson University; 7 Professor, Department of Medicine, and Senior Associate Dean for Academic Affairs, Sidney Kimmel Medical College at Thomas Jefferson University; 8 Associate Professor, Department of Rehabilitation Medicine, Sidney Kimmel Medical College at Thomas Jefferson University; † Co-primary author

**Keywords:** Complexity, Uncertainty, Clinical Teaching/Bedside Teaching, Disabilities, Health Systems, Problem-Solving, Diversity, Equity, Inclusion

## Abstract

**Introduction:**

Uncertainty is an inherent part of medicine. Prior work has trained medical students to better communicate diagnostic uncertainty; however, this work touches on only one aspect of the uncertainty students will face in practice. We developed a session to provide them with a taxonomy for categorizing the various types of uncertainty, as well as a framework to apply when navigating uncertainty during clinical challenges. These tools can help students make sense of uncertainty and determine actions in a complex health system.

**Methods:**

We designed a virtual workshop for third-year medical students at the end of their core clerkships. It included a didactic session followed by a small-group immersive unfolding case experience with several challenge points during which we tasked students with applying the framework, classifying the uncertainty domain, and discussing how they would problem-solve each scenario.

**Results:**

We conducted the workshop with 128 third-year medical students. We collected data through an anonymous postsession survey (86% response rate; 110 of 128 students). Most found the workshop useful (64%; 54 of 85), and a large number found the framework helpful in appraising uncertainty (47%; 42 of 89). A majority felt their perspectives on uncertainty changed after the workshop (66%; 73 of 110). Students integrated prior health systems science knowledge in their strategies to problem-solve each challenge.

**Discussion:**

This session provides a novel application of a sense-making framework and taxonomy for medical students to classify uncertainty. It offers a simple, low-cost, interactive, virtual activity that can be implemented at other institutions.

## Educational Objectives

By the end of this activity, learners will be able to:

1. Appraise specific types of uncertainty they will encounter in clinical practice.
2. Describe a sense-making framework to solve problems when working through clinical uncertainty.
3. Reflect on specific strategies they can apply when navigating complex clinical challenges.

## Introduction

Clinical practice is increasingly complex and uncertain, as highlighted by the recent challenges health care practitioners faced during the COVID-19 pandemic. It is essential that educators equip trainees in the health professions with strategies to better navigate uncertainty in clinical practice.^1^ Current training prepares students in the classroom to approach patient care with evidence-based medicine, but this approach falls short as students enter the clinical realm and are met with the realities of complex clinical care. The gap in training becomes more apparent as medical students transition from the classroom into the clinical environment, where they may be presented with identifying, processing, and providing solutions to various types of clinical uncertainty. These types include not just diagnostic uncertainty but also uncertainty as it relates to aspects of health systems science (HSS), such as patient communication, care coordination, health system improvement, and systems-based practice. One way to help address the uncertainty of clinical practice is to recognize its overlap and integration with HSS and to blend educational strategies in these areas.^2^

Han, Klein, and Arora provide a framework for various types of uncertainty through an integrative conceptual taxonomy.^3^ With this taxonomy, uncertainty can be organized by the specific issue or content at hand. These include scientific (i.e., disease-centered), practical (i.e., system-centered), and personal (i.e., patient-centered) uncertainty. Scientific uncertainty includes the uncertainty surrounding a particular diagnosis, prognosis, and treatment options. Practical uncertainty relates to structures and processes of care, such as uncertainty in wanting to obtain a second opinion or issues accessing care. Lastly, personal uncertainty pertains to the psychosocial and existential issues patients face within health care, such as the uncertainty manifesting in their personal life or belief system. With this framework, students can categorize and start to understand, or make sense of, the uncertainty they encounter.

Snowden and Boone's Cynefin framework has been adapted as a sense-making tool for understanding medical uncertainty.^4,5^ Originating in the business sector, this tool was developed to evaluate decision-making in various contextual environments. Issues can be sorted into four main contexts: simple, complicated, complex, or chaotic.^2,4,5^ In a dynamic clinical environment, physicians can use these four decision-making contexts to better understand their experiences and act accordingly. The simple (known knowns) and complicated (known unknowns) domains are more ordered; cause and effect are easily identifiable, and physicians can determine next steps by reviewing the data and facts presented to them.^2,4,5^ The complex (unknown unknowns) and chaotic (unclear cause and effect) domains are more disordered and require physicians to engage in experimentation to develop new data and solutions.^2,4,5^ The COVID-19 pandemic is a timely example of physicians navigating the uncertainty of the clinical environment and working through the chaotic and complex domains. Physicians first encountered the chaos of COVID-19 and acted swiftly to stabilize patients and respond to the disorder. They then faced not knowing what they did not know and did not have evidence-based practices upon which to act initially. Instead, physicians relied on creativity and critical thinking to allow for experimentation to determine appropriate actions. The Cynefin framework for navigating the clinical environment can be layered upon Han and colleagues’ taxonomy to provide a more comprehensive approach students can take to characterize uncertainty.^3,5^

Prior interventions have aimed to prepare students for the uncertainty of clinical decision-making and the challenges in communicating diagnostic uncertainty.^6–9^ However, none have immersed students in experiences that prompt them to appraise the uncertainty they are working through, categorize, and choose appropriate problem-solving strategies to navigate the clinical uncertainty at hand. Building upon the prior work of Poluch and colleagues,^6^ we addressed this gap in training by designing a virtual evolving case study that would allow medical students to become familiar with these frameworks and problem-solve through various challenges posed by clinical uncertainty.

## Methods

We developed a virtual immersive unfolding case experience for third-year medical students and implemented it at the conclusion of their third-year core clerkships. The session began with a didactic lecture followed by collaborative breakout rooms where students worked together in small groups to analyze the case.

### Case Design

Three experienced medical educators (Mary Stephens, Nethra Ankam, and Dimitrios Papanagnou) developed the case (Appendix A). The case tracked a patient's clinical care trajectory over time, from initial presentation in the emergency department and through the health system as the case unfolded. The scenario followed a woman living with Down syndrome over 3 months as she navigated her diagnosis and management of a newly discovered pituitary mass. The educators built in challenge points that presented students with various aspects of uncertainty and asked them to apply tools and strategies from their prior HSS coursework to solve presented problems. The purpose of the case was not for leaners to determine clinical management but rather to allow them to reflect on how to problem-solve in the setting of uncertainty and determine the best course of action to seek out resources and/or assistance in specific clinical contexts. While there was consensus among content experts as to what decision(s) would be most appropriate for each challenge, there were no correct answers.

The case specifically used disability as a lens to evaluate uncertainty in health care. It was well known that health care providers required further training for managing patients with disabilities.^10^ Without such training, medical students would be likely to struggle with the uncertainty conferred by their diagnoses and physical abilities and could fall victim to diagnostic overshadowing, further adding to the health care disparities facing patients with disabilities.^10^

### Prework and Preparation

We did not include prework as part of this design. Offered as part of a series, this workshop was a second, stand-alone session that followed a course on preparing students to communicate diagnostic uncertainty during transitions in care. (This course has been previously published in *MedEdPORTAL.*^6^) Completion of the communication course, however, was not required for this workshop. There were also tie-ins to knowledge gained from our students’ longitudinal vertically integrated HSS curricular thread, though similar knowledge would likely be gained after the completion of core clerkships. While we opted for this format at our medical school, it need not be replicated. The current session could be offered to students without any prework or preparation.

### Equipment

This virtual workshop required minimal equipment for implementation. We used Zoom and leveraged its breakout room capabilities to host the session. All students needed computers or tablets with internet functionality, webcams, and microphones for full participation and engagement. We provided links ahead of time for students to access the virtual session. We conducted the didactic portion by presenting a PowerPoint file through host screen sharing. We delivered the interactive case using Nearpod (Renaissance Holding Corporation), an application-based digital tool that allowed slide sharing and activity integration, in order for students to submit answers in real time for faculty review. Although we chose Nearpod, educators could pick any format to collect student responses, including Zoom's chat function or an electronic survey form.

### Personnel

Two main faculty members facilitated the session. We recruited three additional facilitators to assist with managing breakout rooms, recording the virtual session, providing links to supplemental materials (Appendix B) as needed, keeping time for the breakout rooms, and reviewing the challenge responses as they were submitted in real time. The two main faculty also facilitated the debrief at the end of the virtual case. All facilitators were practicing physicians with direct patient care experiences and faculty appointments within the medical school.

### Implementation

The students first gathered on the virtual platform for a 20-minute didactic lecture (Appendix C) that introduced medical uncertainty, the uncertainty taxonomy, and the Cynefin framework. We provided students with examples of applying these concepts in health care. In addition, we introduced the students to the disparities that patients with disabilities face.

We then provided students with instructions on case analysis, as well as supplemental materials (Appendix B) to refer to as they worked through the case. We assigned students randomly to breakout rooms of 10 students each. We instructed each group to identify one student to share their screen within the small group and capture the group's responses to the eight case challenges presented. We allotted 5 minutes for each of the challenges. The facilitator advanced the slides for the groups remotely and provided all groups with time notifications as 2-minute and 1-minute warnings. Students submitted responses to each of the following questions for each challenge presented:

• *Sense-making:* Applying the Cynefin framework, appraise the uncertainty you are working through. What quadrant are you working in? Try to make sense of why the situation is uncertain to you.
• *Naming the uncertainty:* Classify the medical content domain of the uncertainty (as it pertains to the diagnosis, prognosis, treatment, etc.).
• *Problem-solving:* Discuss how you would problem-solve in this situation. What resources would you seek out? What communication strategies would you use?

Facilitators reviewed the responses as students submitted them and later highlighted noteworthy ones in the debriefing to stimulate further conversation. At the conclusion of the case, students exited their breakout rooms and reentered the main virtual room for a facilitated debriefing.

### Debriefing

The main facilitators engaged with students in the main virtual room to review the case challenge points and share some of the noteworthy responses. The facilitators also used additional prompts to help facilitate the discussion, as detailed in Appendix D.

### Assessment

After the debriefing, we asked all students to follow a link to complete an anonymous postsession questionnaire (Appendix E). We created this survey and determined items through consensus agreement. As a result, we asked a mix of open- and closed-ended questions to determine students’ attitudes about the session. To gauge knowledge acquisition, we asked students to share one strategy they intended to apply when problem-solving through uncertainty in future clinical environments. We exported survey data into Excel spreadsheets for analysis. For quantitative data, we reported proportions as percentages and continuous variables as medians with quartiles. For qualitative data, we used open- and axial-coding methods to analyze individual open-ended responses, identify concepts, and categorize responses into themes.

## Results

We conducted this session with 128 third-year medical students at the conclusion of their core clerkships. Most of the students responded to the postsession survey (response rate 86%; 110 of 128 students). We evaluated the data for each question independently since all respondents did not complete each question.

Many students found the workshop moderately, very, or extremely useful (64%; 54 of 85) and most (62%; 51 of 82) felt the workshop should be included in the future curriculum. Students indicated several areas where they felt uncomfortable navigating uncertainty in clinical practice, with the least comfort surrounding prognostic uncertainty (21%; 32 of 153). While not representing the majority, a large number of students found the Cynefin framework to be a helpful tool (yes/no) when appraising uncertainty in clinical practice (47%; 42 of 89). Most students felt that their perspectives surrounding uncertainty in clinical practice changed (yes/no) after this session (66%; 73 of 110).

Students shared various strategies to apply when problem-solving through uncertainty in the clinical environment. While this was not a qualitative study, we made an effort to categorize these responses into a set of themes. The themes included applying a framework to navigate uncertainty, acknowledging uncertainty and asking for help, open communication, team collaboration, patient-centered care, and preparation. Representative quotes from the three most prominent themes are provided below:

• Acknowledging uncertainty and asking for help: “Asking for help in times of uncertainty.”
• Applying a framework to navigate uncertainty: “There will always be aspects of uncertainty in clinical practice [and] addressing it systematically will be helpful.”
• Open communication: “Having lots of open communication with patients and interdisciplinary collaboration to foster joint decision making.”

We also asked students for feedback on the utility of the Cynefin framework and how to improve the workshop for future sessions. Some key insights included the following:

• “As we went through the challenges it definitely helped us think about how to process information related to our situation and include the uncertainty as an active issue that needs to be regularly evaluated.”
• “Helped me identify specific areas of uncertainty within larger intricate issues.”
• “Give 2–3 practice examples prior to the breakout groups so that the breakout groups hit the ground running.”

## Discussion

While the concepts of sense-making and appraising uncertainty have been introduced in the literature, no strategies to include them as educational experiences in a medical education program have been described, especially for students in the nascent stages of their clinical training.^2,3,5^ Our uncertainty workshop successfully introduced a sense-making framework and taxonomy for classifying uncertainty to medical students as they entered the clinical learning environment. Medical students participating in our workshop found it useful in preparing them for problem-solving during times of clinical uncertainty. Students successfully applied the Cynefin framework to identify diverse types of uncertain scenarios they faced and appropriately classified the medical content domains as defined by the taxonomy.^2^ Students demonstrated an array of problem-solving strategies that integrated prior HSS knowledge, such as patient advocacy, patient-centered communication, care coordination, interprofessional collaboration, managing social and structural determinants of health, transitions of care, medical error and error disclosure, shared decision-making, and identifying patient resources. Furthermore, our case was successful in illustrating the complexities and interconnectedness of care for persons living with disabilities, as we tasked students with navigating challenge points that addressed various biases in care. Our workshop can serve as an opportunity to address disability and the biases and microaggressions that frequently accompany caring for patients living with a disability.^11^

Given that we designed our workshop to prompt students to critically reflect on experiences of clinical uncertainty and their problem-solving strategies, we did not develop it to include the use of a pre- or posttest. Additionally, we designed challenge points to invoke problem-solving without formal correct answers. Our review of student responses, followed by a debriefing, allowed for a more formative assessment of student comprehension of the educational objectives. Further assessment of student learning is limited due to these design aspects. In addition, we developed our session for third-year medical students as they transition into the fourth year of medical school, after having completed core clerkships. Because of this, as well as the integration of prior HSS knowledge, generalizability may be limited to learners with basic foundational clinical experience. While HSS is presented longitudinally across our students’ curriculum, the problem-solving strategies they are prompted to apply likely reflect core content also gained from the clerkships. Therefore, even for medical schools without a dedicated HSS curriculum, this experience is likely generalizable to students after completion of core clerkships yet may not be as readily applied to preclinical medical students.

Interestingly, session feedback showed that 40% of students did not find the Cynefin framework helpful. Student feedback requesting additional examples of applying the framework and classification system prior to entering the breakout rooms also reflected this. We hypothesize that the didactic portion may not have had an adequate amount of time to introduce this sophisticated topic and framework and that students could have benefited from prework, such as supplemental readings. Students also recommended shortening the number of challenge points. The time gained from reducing the number of challenges could be applied to better explaining the Cynefin framework and complexity science. Additional feedback received regarded breakout rooms and concerns about unequal participation by students within the small groups. These concerns can be mitigated by having facilitators intermittently join the breakout rooms to encourage student participation and address any questions students may have about the exercise. We caution, however, that this strategy be weighed against the potential harm of invoking anxiety and inhibiting students’ authentic conversations surrounding their problem-solving.

Our workshop provides a virtual learning activity that is simple, low cost, and interactive and can be easily implemented at other institutions. If desired, the workshop can be readily adapted to an in-person format. In addition, the prior course our students completed on communicating diagnostic uncertainty is a complementary educational session and can be considered part of a series.^6^ Furthermore, the workshop can be expanded to include additional cases and integrated throughout a curriculum to allow for spaced-repetition instruction on these concepts. These strategies would aid educators in creating a robust educational experience on uncertainty within clinical care. While the workshop was created for third-year medical students as part of their transition to the fourth year of medical school, we believe it has utility in graduate medical education, as well as in various other health professions education, to better prepare all trainees to reflect on their skills in navigating the complexity and uncertainty of clinical practice.

## Appendices


Case Slides.pptxStudent Instructions.docxUncertainty Didactic Slides.pptxFacilitator Instructions.docxPostsession Survey.docx
*All appendices are peer reviewed as integral parts of the Original Publication.*

## References

[1] Herzog L. COVID-19: unveiling the role of uncertainty in medical education [version 1]. MedEdPublish. 2020;9:166. 10.15694/mep.2020.000166.1 PMC10702646 38073849

[2] Papanagnou D, Jaffe R, Ziring D. Highlighting a curricular need: uncertainty, COVID-19, and health systems science. Health Sci Rep. 2021;4(3):e363. 10.1002/hsr2.363 34485705 PMC8407290

[3] Han PKJ, Klein WMP, Arora NK. Varieties of uncertainty in health care: a conceptual taxonomy. Med Decis Making. 2011;31(6):828–838. 10.1177/0272989X10393976 22067431 PMC3146626

[4] Papanagnou D, Watkins KE, Lundgren H, Alcid GA, Ziring D, Marsick VJ. Informal and incidental learning in the clinical learning environment: learning through complexity and uncertainty during COVID-19. Acad Med. 2022;97(8):1137–1143. 10.1097/ACM.0000000000004717 35476789 PMC9311294

[5] Snowden DJ, Boone ME. A leader's framework for decision making. Harv Bus Rev. 2007;85(11):68–76. 18159787

[6] Poluch M, Feingold-Link J, Ankam N, et al. I don't have a diagnosis for you: preparing medical students to communicate diagnostic uncertainty in the emergency department. MedEdPORTAL. 2022;18:11218. 10.15766/mep_2374-8265.11218 35178469 PMC8814030

[7] Willett L. Uncertain times: clinical decision-making in sickness and in health. MedEdPORTAL. 2013;9:9620. 10.15766/mep_2374-8265.9620

[8] Rising KL, Powell RE, Cameron KA, et al. Development of the Uncertainty Communication Checklist: a patient-centered approach to patient discharge from the emergency department. Acad Med. 2020;95(7):1026–1034. 10.1097/ACM.0000000000003231 32101919 PMC7302334

[9] Ho B, Ishizaki A, Ko A, et al. Kim Lee: problem-based learning (PBL) case for delivering uncertain news. MedEdPORTAL. 2013;9:9525. 10.15766/mep_2374-8265.9525

[10] Bowen CN, Havercamp SM, Karpiak Bowen S, Nye G. A call to action: preparing a disability-competent health care workforce. Disabil Health J. 2020;13(4):100941. 10.1016/j.dhjo.2020.100941 32467076

[11] Iezzoni LI, Rao SR, Ressalam J, et al. Physicians’ perceptions of people with disability and their health care. *Health Aff (Millwood)*. 2021;40(2):297–306. 10.1377/hlthaff.2020.01452 PMC8722582 33523739

