# Supplementary material for: Navigating Uncertainty in Clinical Practice: A Workshop to Prepare Medical Students to Problem-Solve During Complex Clinical Challenges
Source: MedEdPORTAL. 2023 Aug 9;19:11334. doi: 10.15766/mep_2374-8265.11334 (PMC10409886; doi:10.15766/mep_2374-8265.11334)
Supplement: Supplementary file 1 — Case Slides.pptxStudent Instructions.docxUncertainty Didactic Slides.pptxFacilitator Instructions.docxPostsession Survey.docx [file mep_2374-8265.11334-s001.zip › A. Case Slides.pptx]

## Slide 1
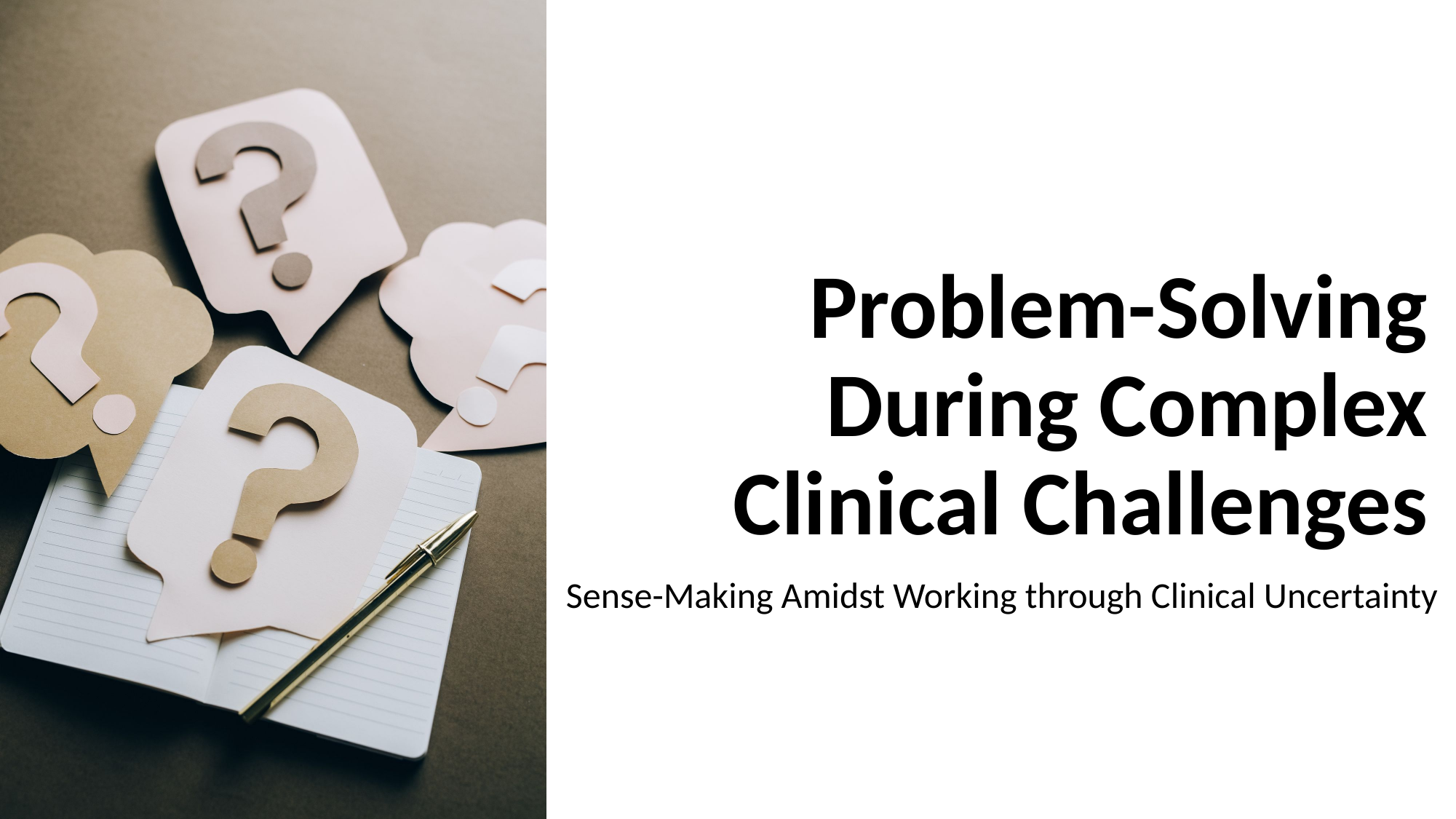

# Problem-Solving During Complex Clinical Challenges
Sense-Making Amidst Working through Clinical Uncertainty

## Slide 2
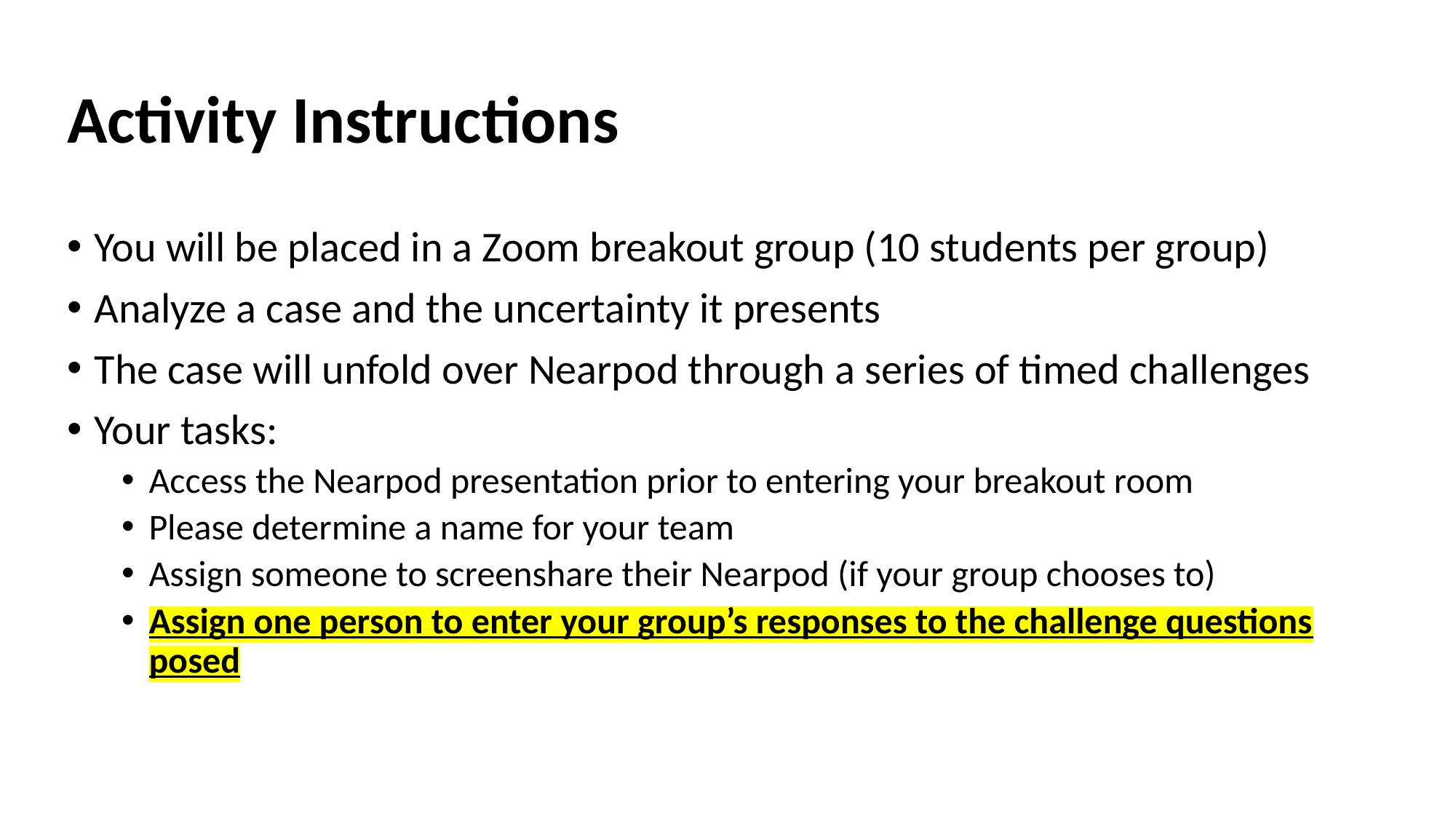

# Activity Instructions
You will be placed in a Zoom breakout group (10 students per group)
Analyze a case and the uncertainty it presents
The case will unfold over Nearpod through a series of timed challenges
Your tasks:
Access the Nearpod presentation prior to entering your breakout room
Please determine a name for your team
Assign someone to screenshare their Nearpod (if your group chooses to)
Assign one person to enter your group’s responses to the challenge questions posed

## Slide 3
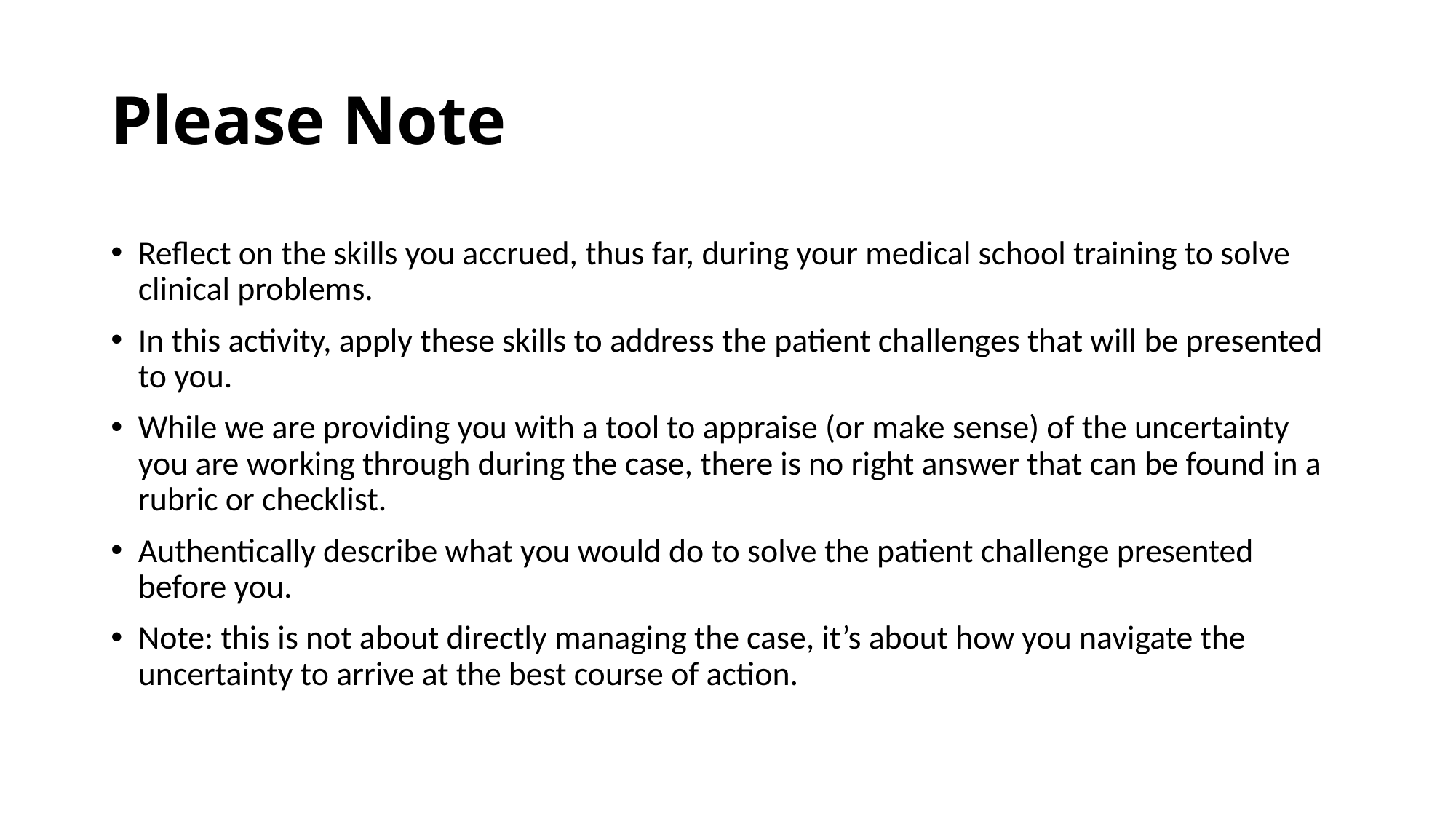

# Please Note
Reflect on the skills you accrued, thus far, during your medical school training to solve clinical problems.
In this activity, apply these skills to address the patient challenges that will be presented to you.
While we are providing you with a tool to appraise (or make sense) of the uncertainty you are working through during the case, there is no right answer that can be found in a rubric or checklist.
Authentically describe what you would do to solve the patient challenge presented before you.
Note: this is not about directly managing the case, it’s about how you navigate the uncertainty to arrive at the best course of action.

## Slide 4
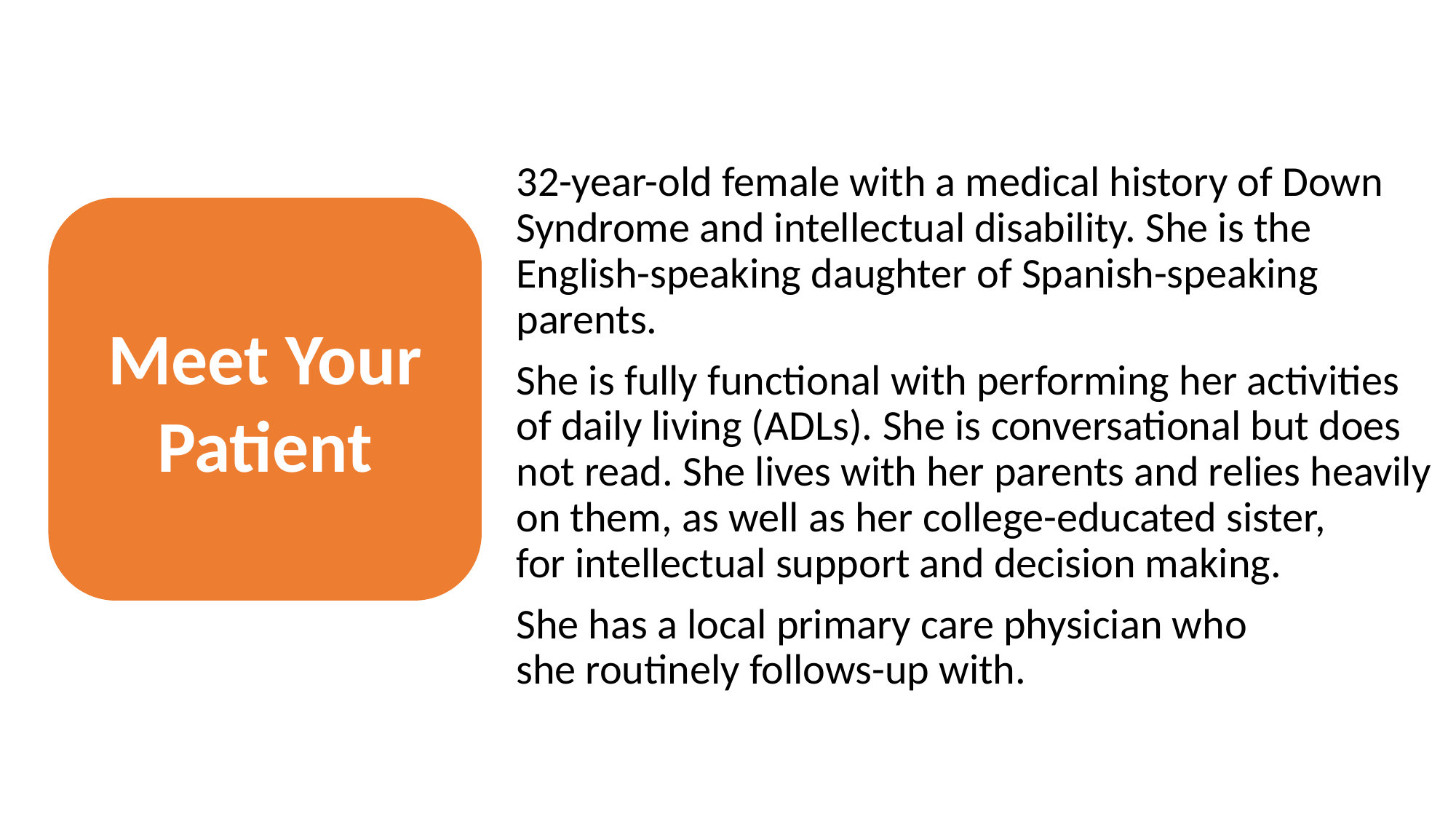

32-year-old female with a medical history of Down Syndrome and intellectual disability. She is the English-speaking daughter of Spanish-speaking parents. ​
She is fully functional with performing her activities of daily living (ADLs). She is conversational but does not read. She lives with her parents and relies heavily on them, as well as her college-educated sister, for intellectual support and decision making. ​
She has a local primary care physician who she routinely follows-up with. ​
Meet Your Patient

## Slide 5
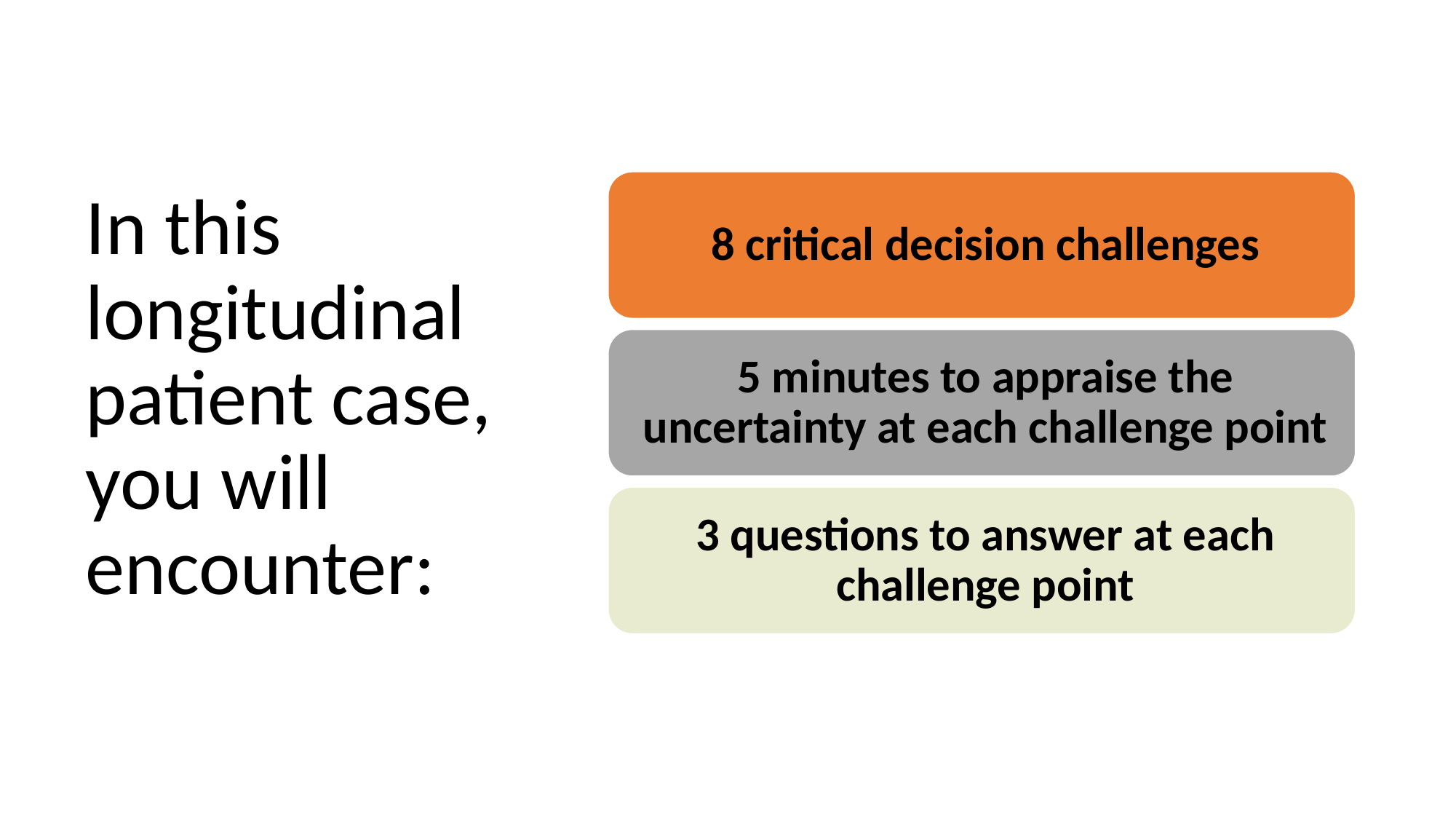

# In this longitudinal patient case, you will encounter:

## Slide 6
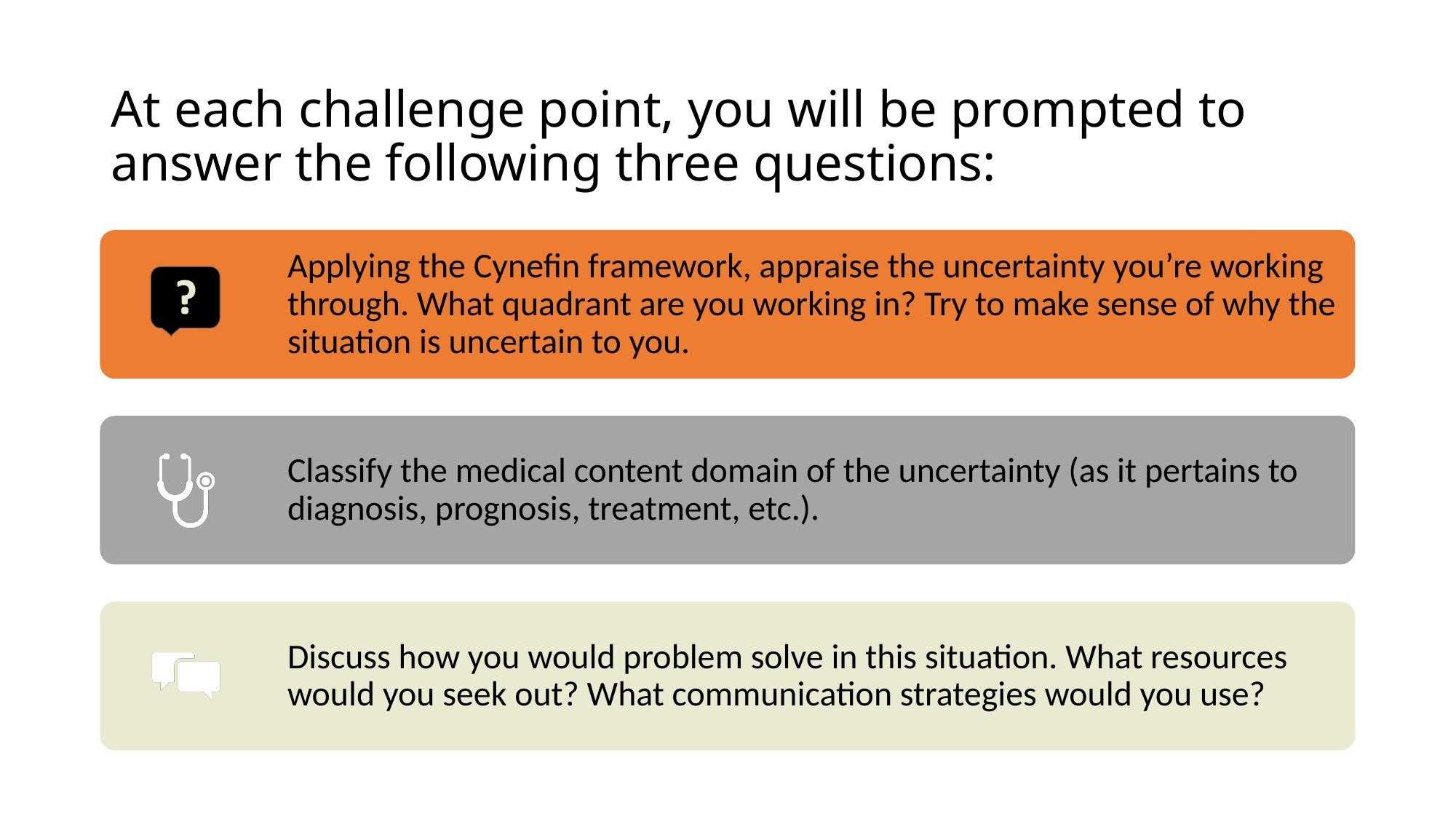

# At each challenge point, you will be prompted to answer the following three questions:

## Slide 7
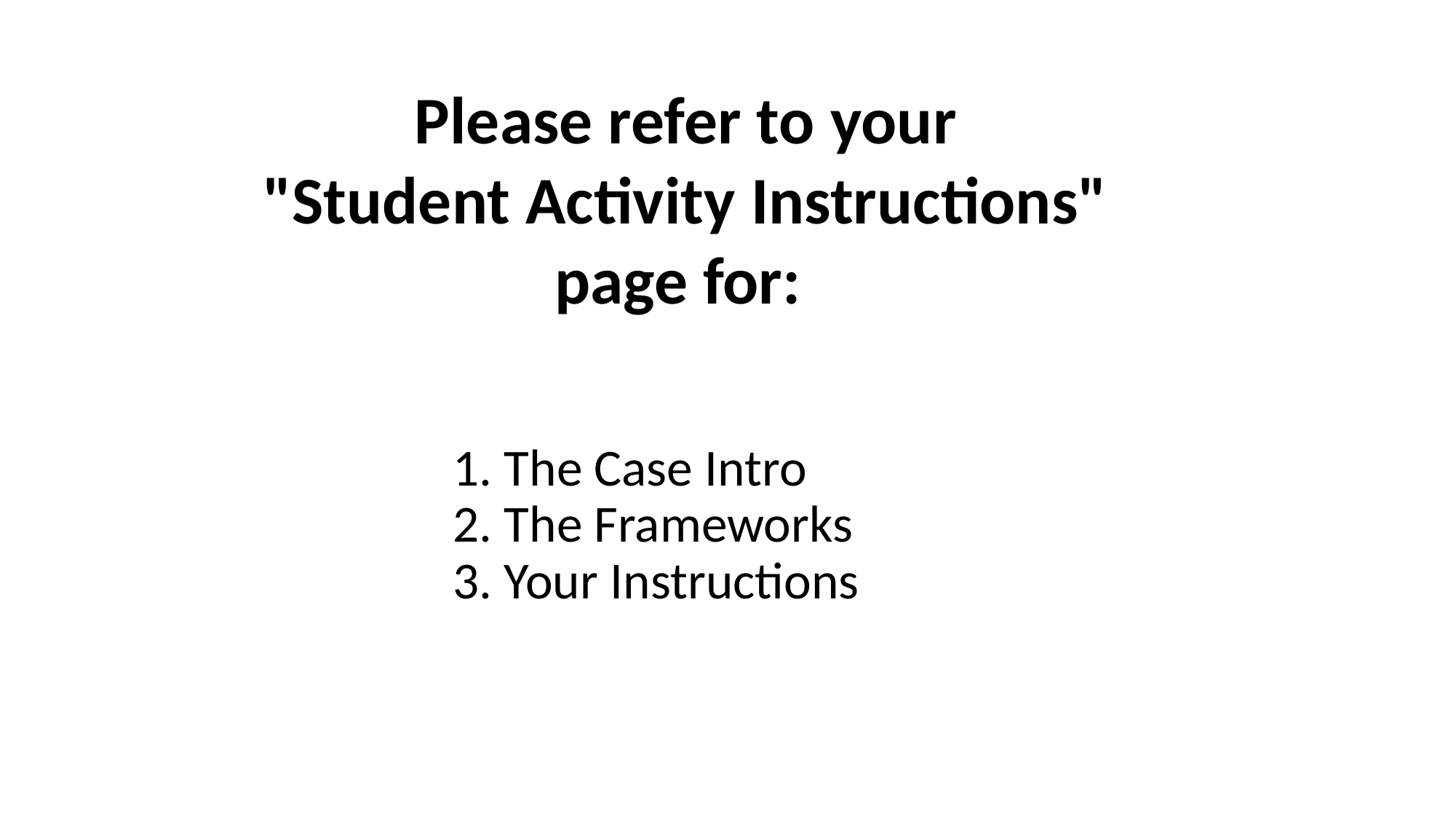

Please refer to your
 "Student Activity Instructions"
page for:
# 1. The Case Intro 2. The Frameworks 3. Your Instructions

## Slide 8
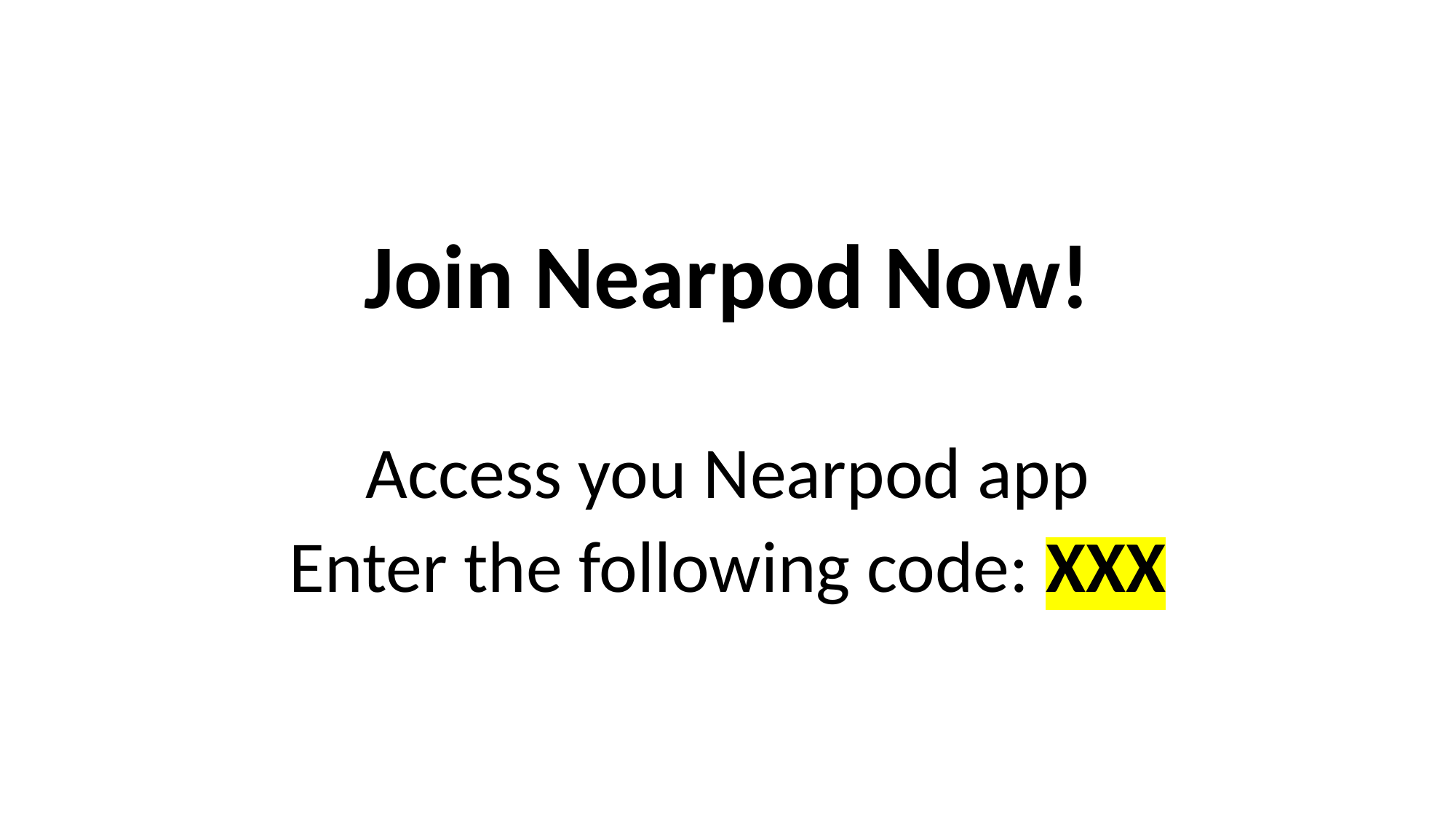

# Join Nearpod Now!
Access you Nearpod app
Enter the following code: XXX

## Slide 9
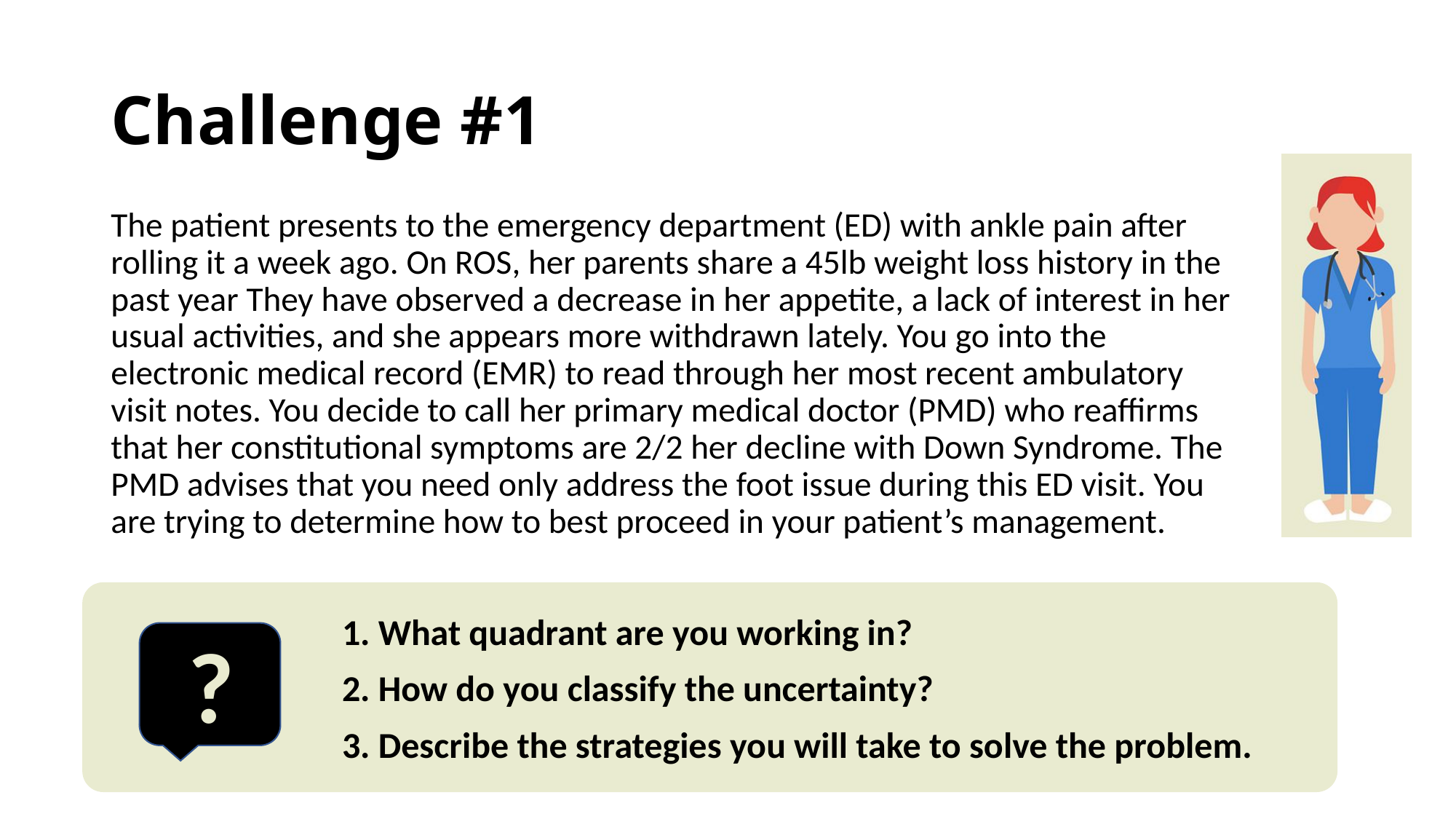

# Challenge #1
The patient presents to the emergency department (ED) with ankle pain after rolling it a week ago. On ROS, her parents share a 45lb weight loss history in the past year They have observed a decrease in her appetite, a lack of interest in her usual activities, and she appears more withdrawn lately. You go into the electronic medical record (EMR) to read through her most recent ambulatory visit notes. You decide to call her primary medical doctor (PMD) who reaffirms that her constitutional symptoms are 2/2 her decline with Down Syndrome. The PMD advises that you need only address the foot issue during this ED visit. You are trying to determine how to best proceed in your patient’s management.
1. What quadrant are you working in?
2. How do you classify the uncertainty?
3. Describe the strategies you will take to solve the problem.
?

## Slide 10
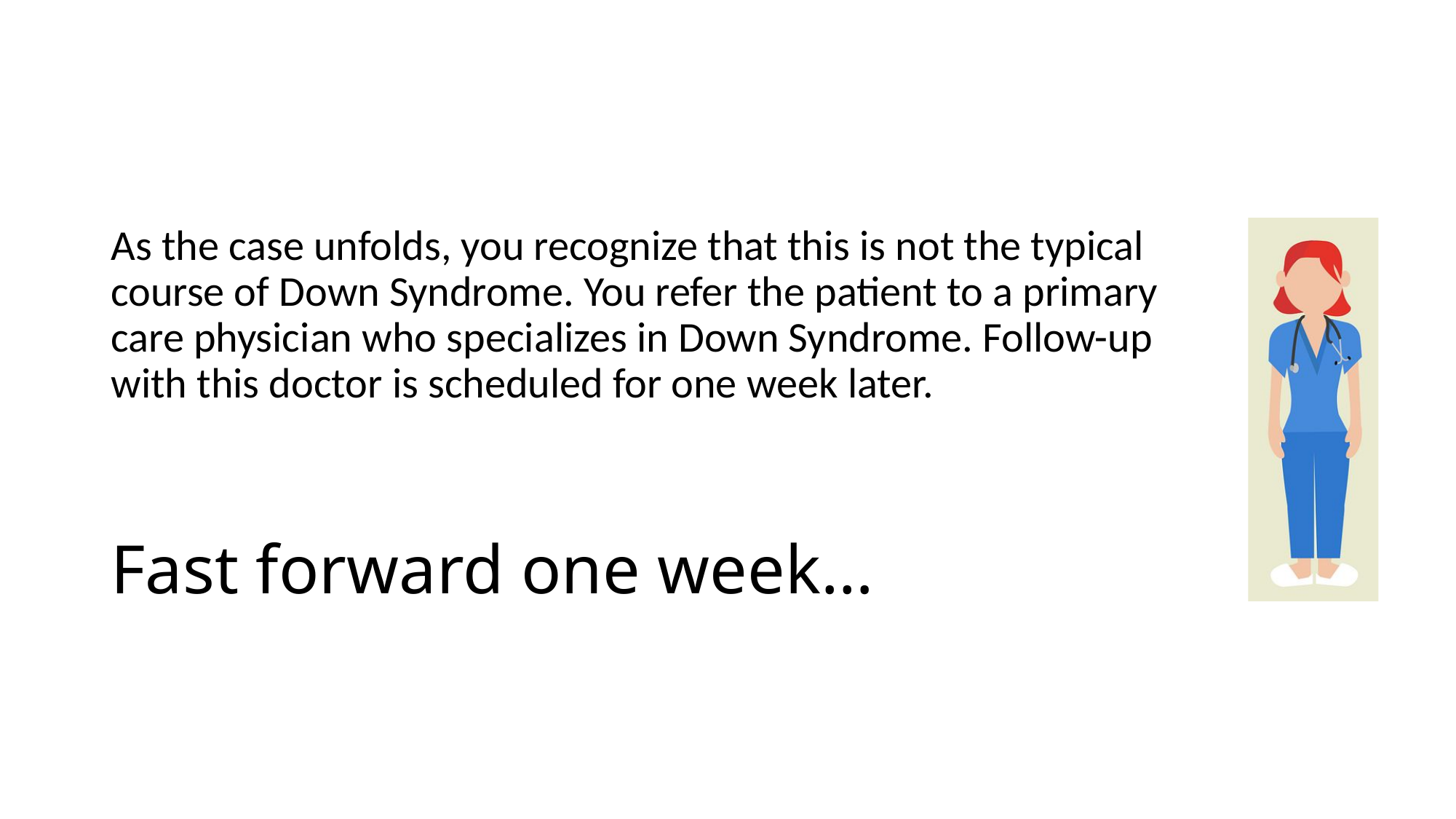

As the case unfolds, you recognize that this is not the typical course of Down Syndrome. You refer the patient to a primary care physician who specializes in Down Syndrome. Follow-up with this doctor is scheduled for one week later.
# Fast forward one week…

## Slide 11
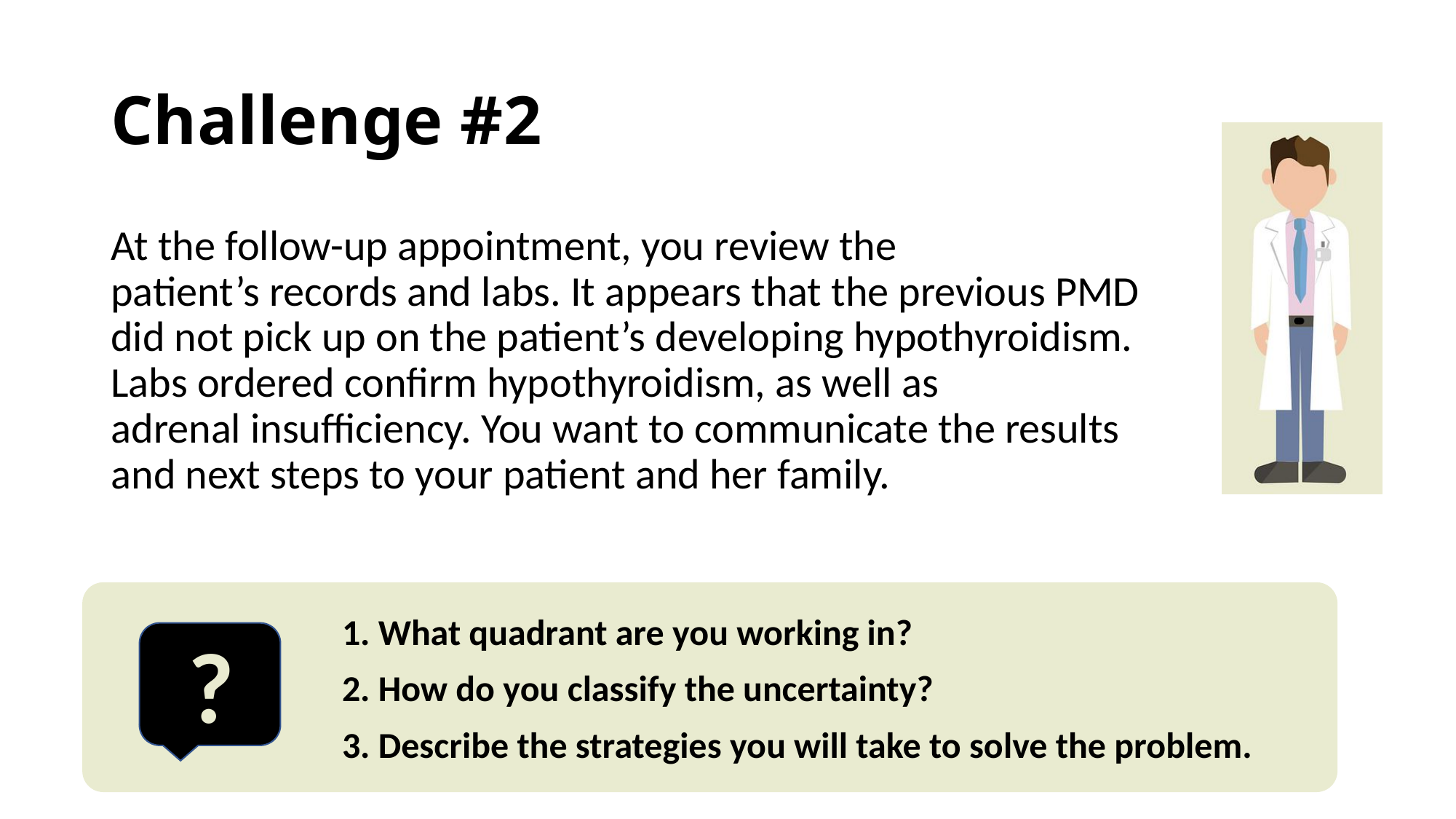

# Challenge #2
At the follow-up appointment, you review the patient’s records and labs. It appears that the previous PMD did not pick up on the patient’s developing hypothyroidism. Labs ordered confirm hypothyroidism, as well as adrenal insufficiency. You want to communicate the results and next steps to your patient and her family. ​
1. What quadrant are you working in?
2. How do you classify the uncertainty?
3. Describe the strategies you will take to solve the problem.
1. What quadrant are you working in?
2. How do you classify the uncertainty?
3. Describe the strategies you will take to solve the problem.
?

## Slide 12
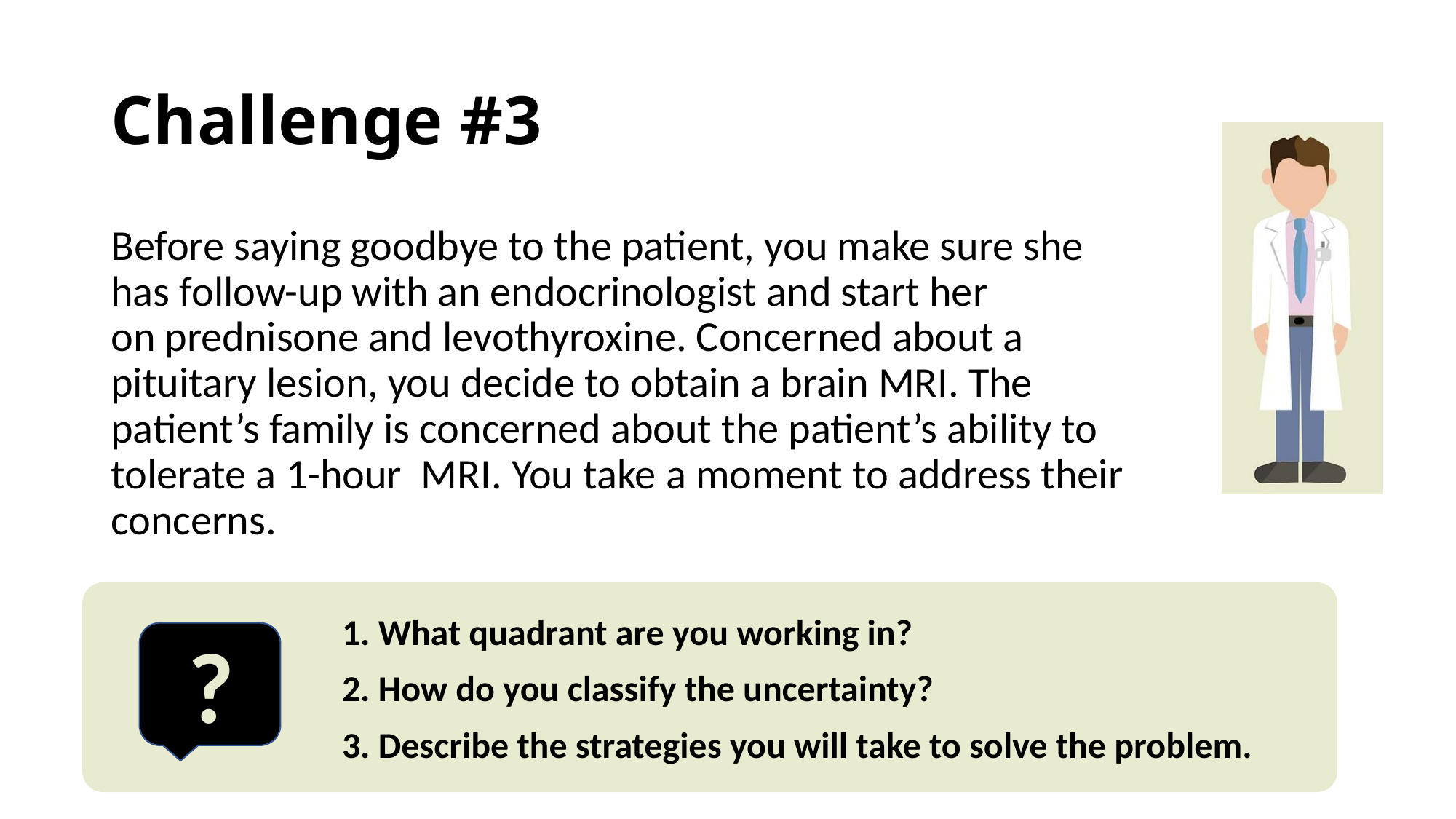

# Challenge #3
Before saying goodbye to the patient, you make sure she has follow-up with an endocrinologist and start her on prednisone and levothyroxine. Concerned about a pituitary lesion, you decide to obtain a brain MRI. The patient’s family is concerned about the patient’s ability to tolerate a 1-hour  MRI. You take a moment to address their concerns.  ​
1. What quadrant are you working in?
2. How do you classify the uncertainty?
3. Describe the strategies you will take to solve the problem.
?

## Slide 13
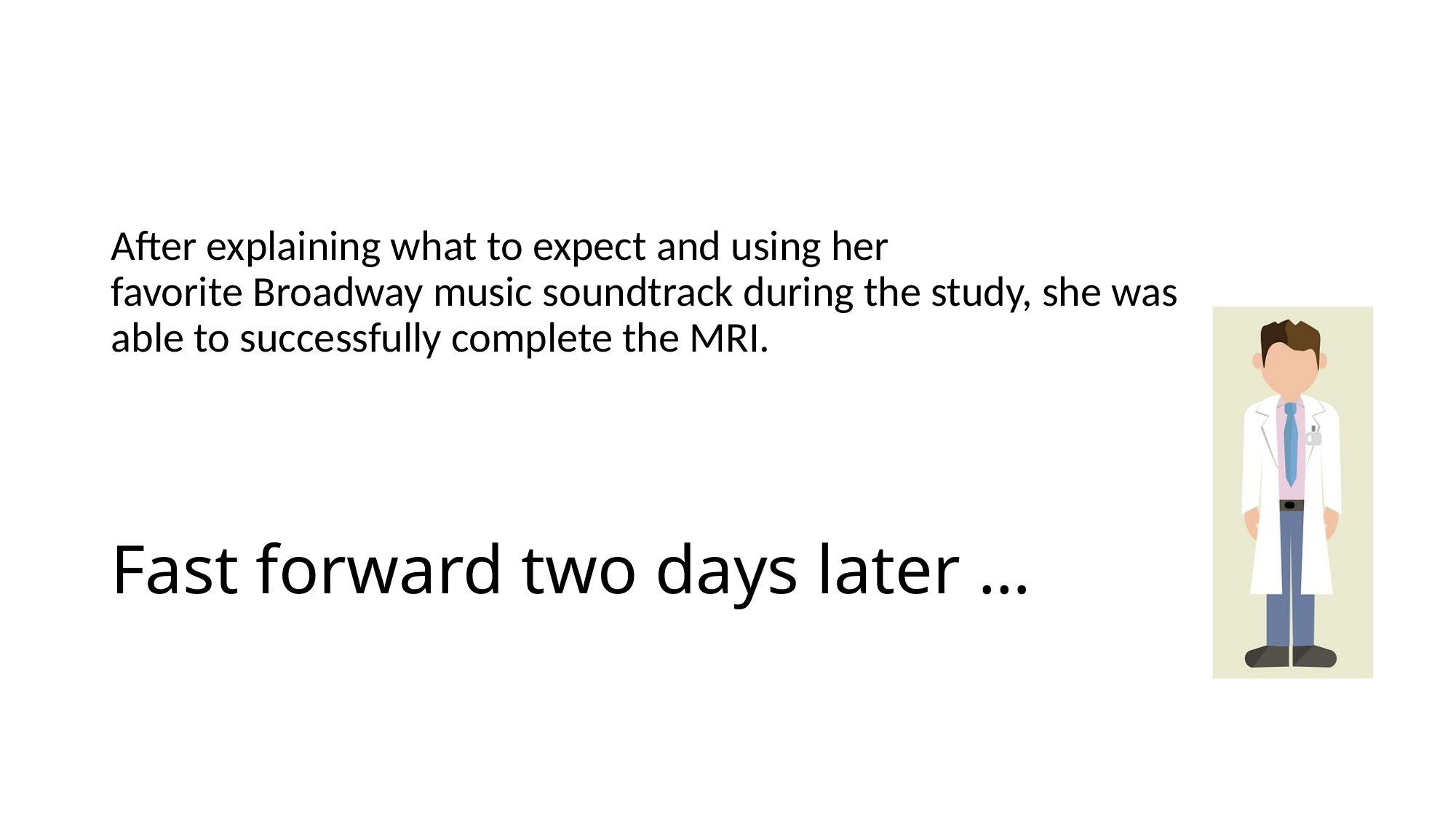

After explaining what to expect and using her favorite Broadway music soundtrack during the study, she was able to successfully complete the MRI. ​
# Fast forward two days later …

## Slide 14
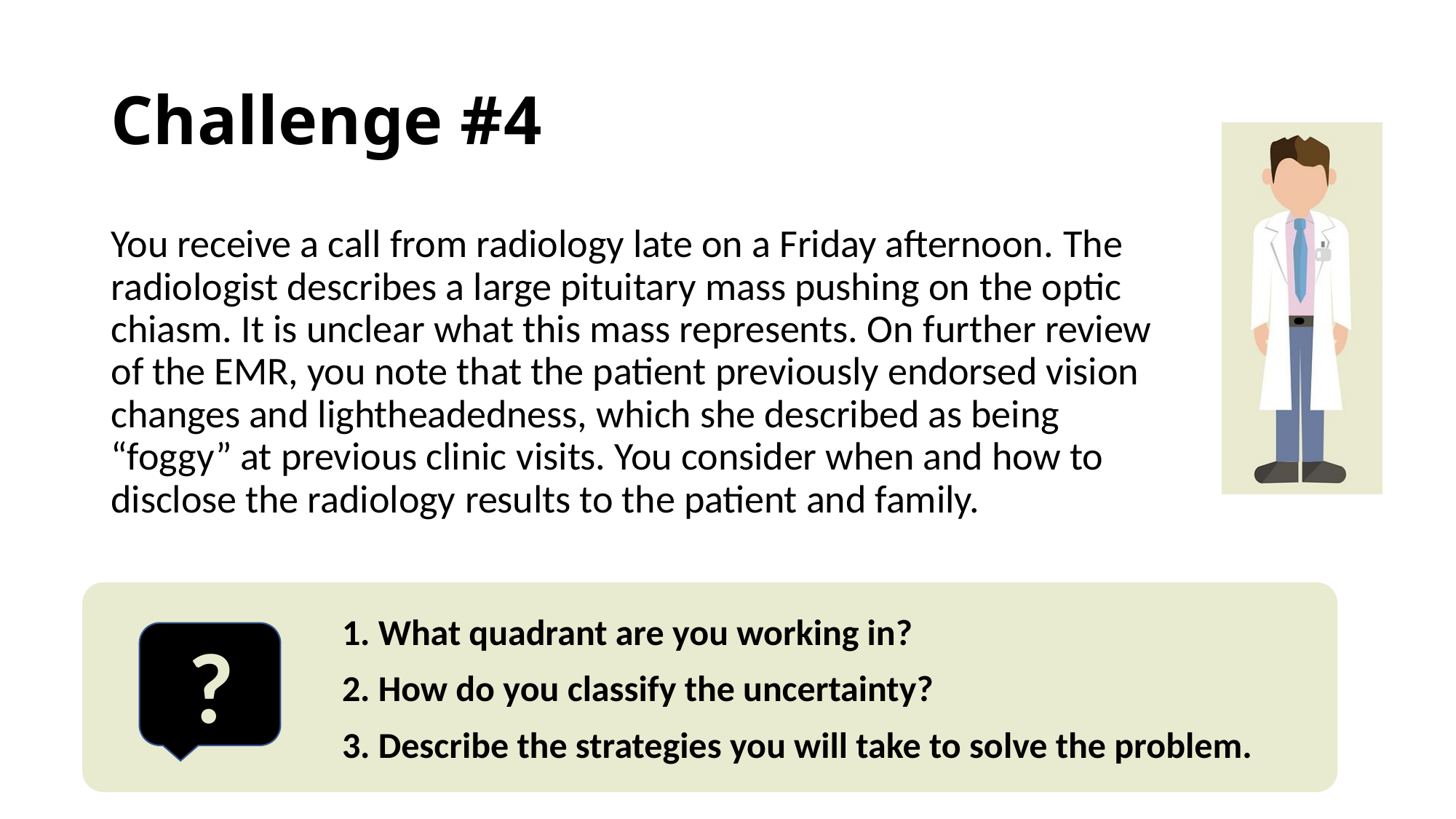

# Challenge #4
You receive a call from radiology late on a Friday afternoon. The radiologist describes a large pituitary mass pushing on the optic chiasm. It is unclear what this mass represents. On further review of the EMR, you note that the patient previously endorsed vision changes and lightheadedness, which she described as being “foggy” at previous clinic visits. You consider when and how to disclose the radiology results to the patient and family.  ​
1. What quadrant are you working in?
2. How do you classify the uncertainty?
3. Describe the strategies you will take to solve the problem.
?

## Slide 15
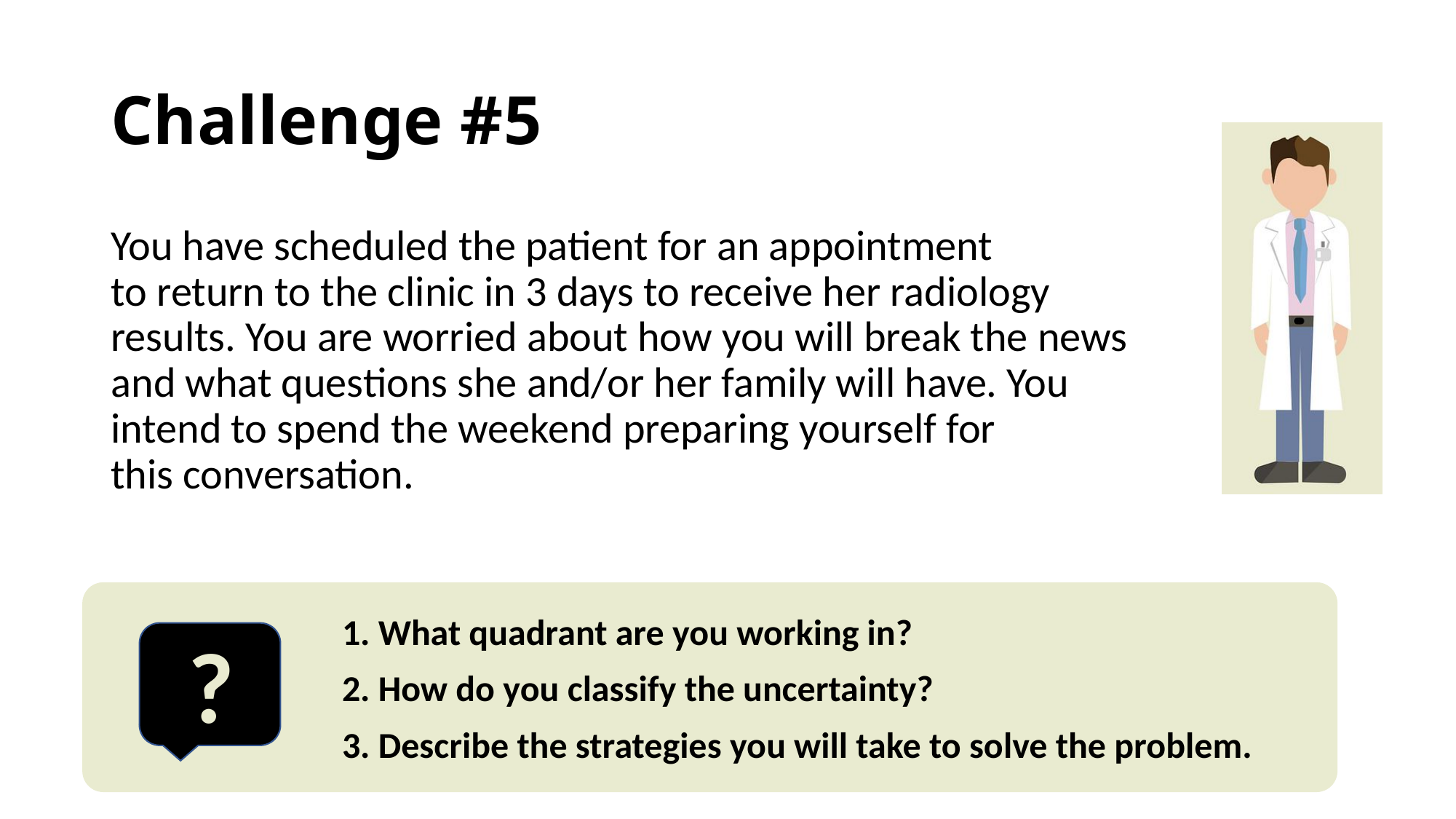

# Challenge #5
You have scheduled the patient for an appointment to return to the clinic in 3 days to receive her radiology results. You are worried about how you will break the news and what questions she and/or her family will have. You intend to spend the weekend preparing yourself for this conversation. ​
1. What quadrant are you working in?
2. How do you classify the uncertainty?
3. Describe the strategies you will take to solve the problem.
?

## Slide 16
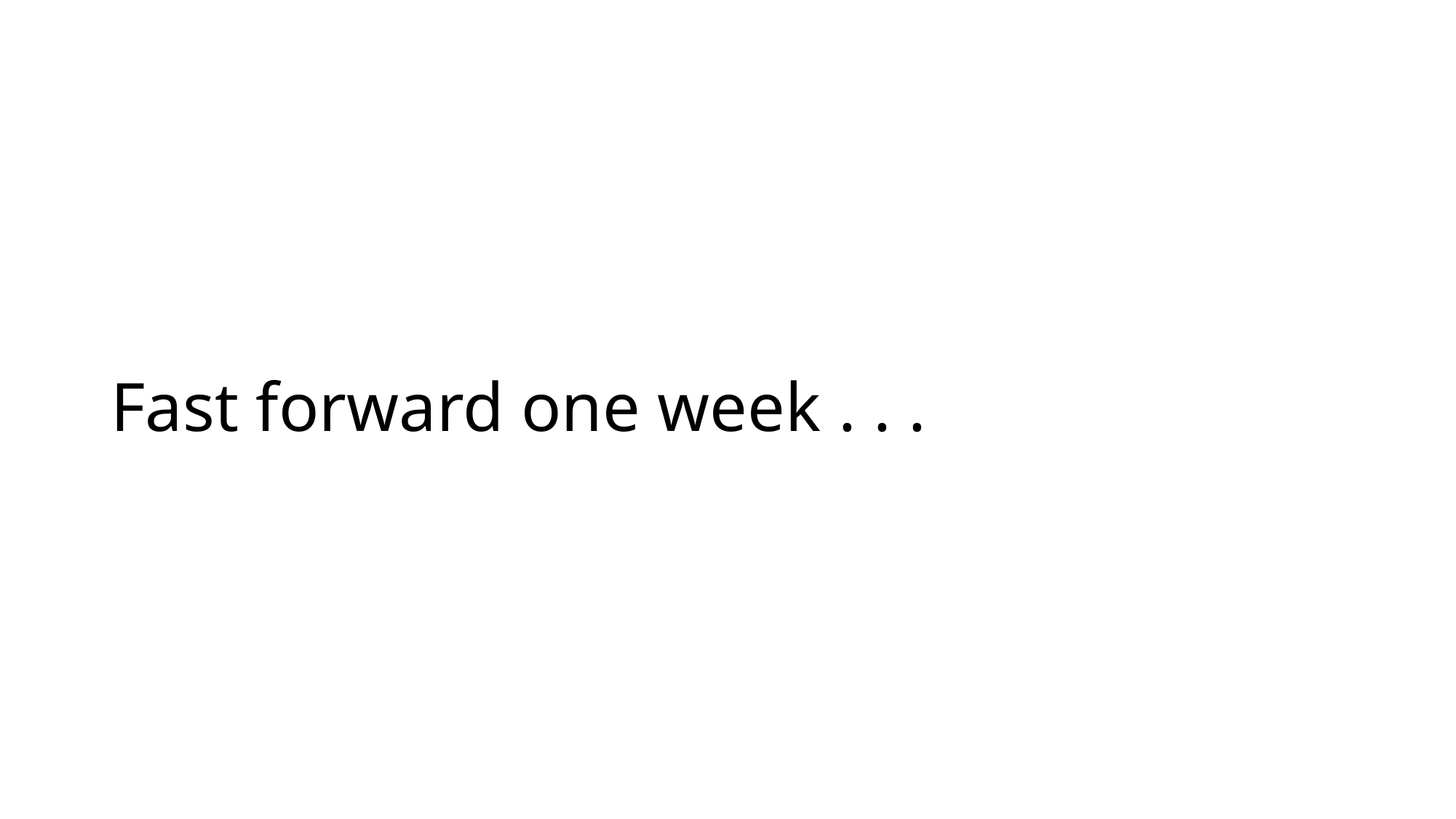

# Fast forward one week . . .

## Slide 17
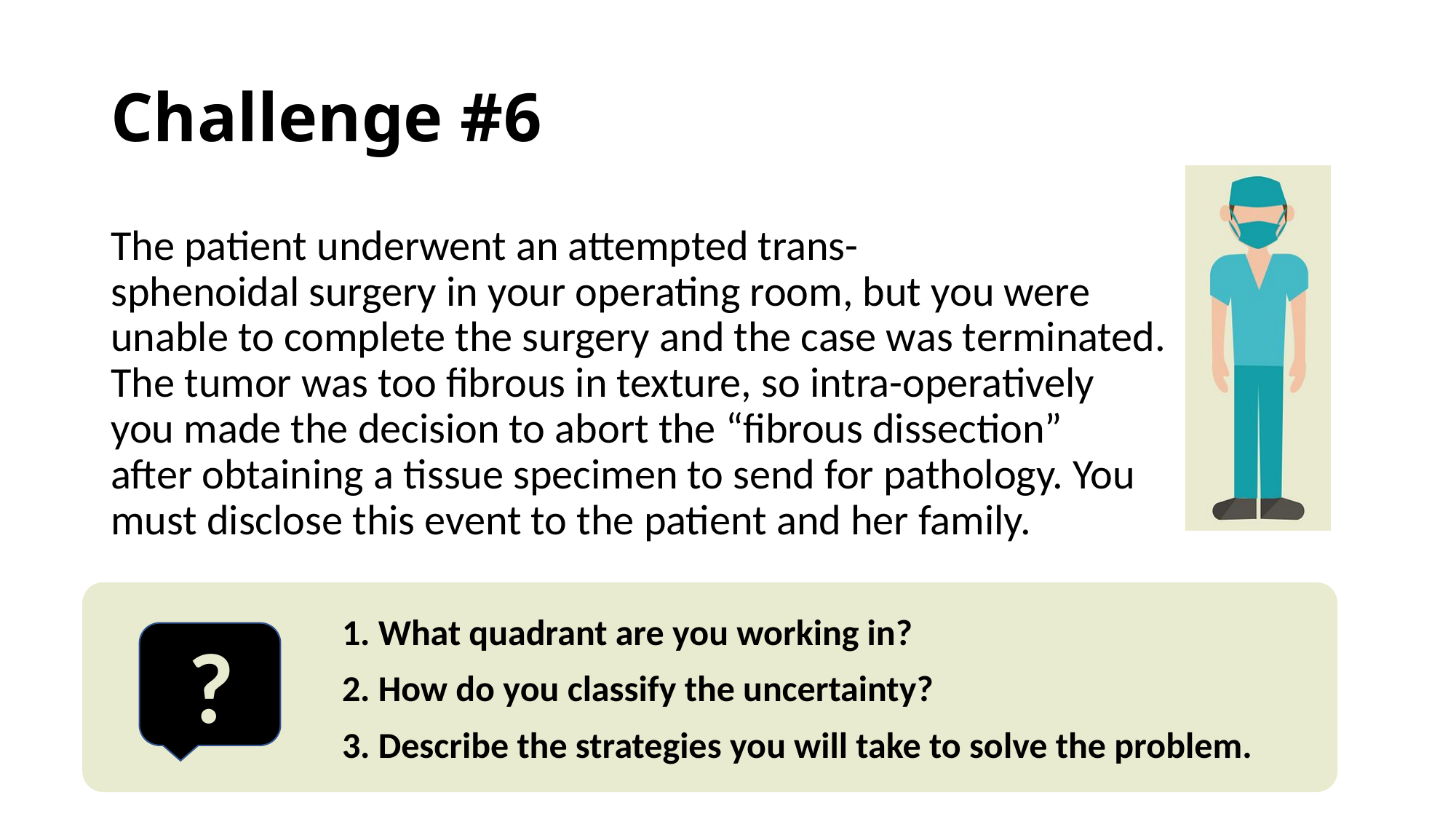

# Challenge #6
The patient underwent an attempted trans-sphenoidal surgery in your operating room, but you were unable to complete the surgery and the case was terminated. The tumor was too fibrous in texture, so intra-operatively you made the decision to abort the “fibrous dissection” after obtaining a tissue specimen to send for pathology. You must disclose this event to the patient and her family.​
1. What quadrant are you working in?
2. How do you classify the uncertainty?
3. Describe the strategies you will take to solve the problem.
?

## Slide 18
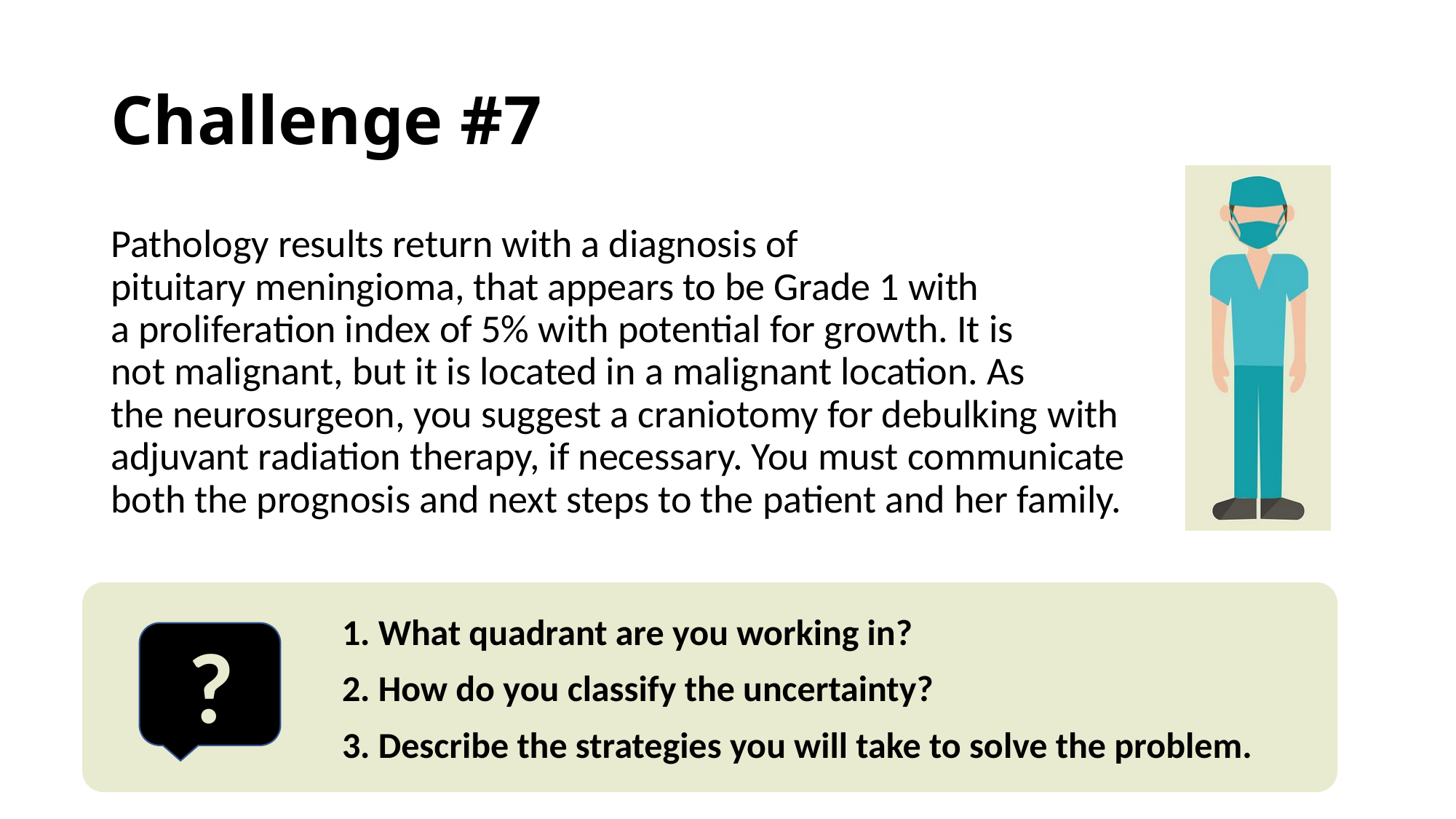

# Challenge #7
Pathology results return with a diagnosis of pituitary meningioma, that appears to be Grade 1 with a proliferation index of 5% with potential for growth. It is not malignant, but it is located in a malignant location. As the neurosurgeon, you suggest a craniotomy for debulking with adjuvant radiation therapy, if necessary. You must communicate both the prognosis and next steps to the patient and her family. ​
1. What quadrant are you working in?
2. How do you classify the uncertainty?
3. Describe the strategies you will take to solve the problem.
?

## Slide 19
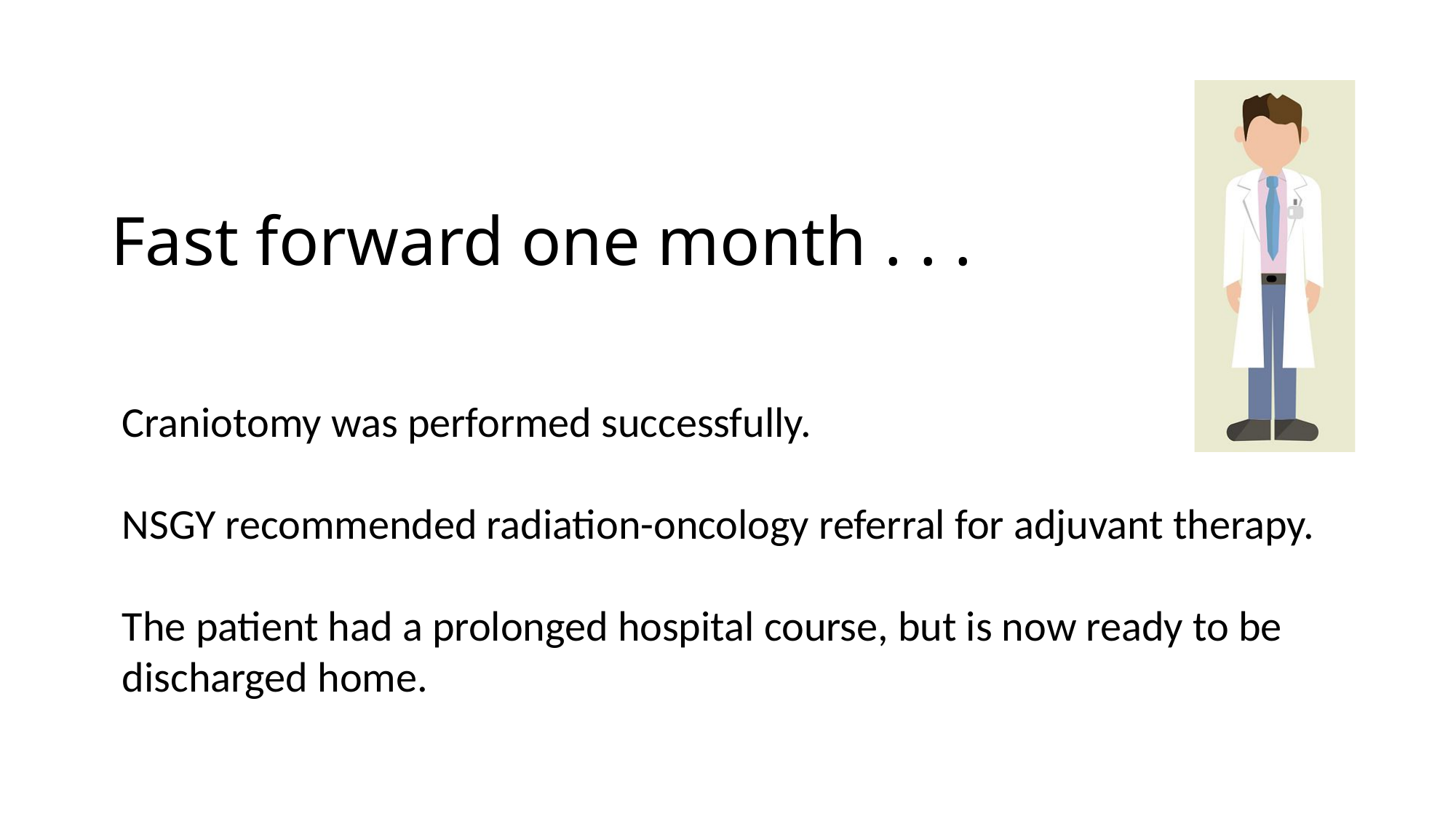

# Fast forward one month . . .
Craniotomy was performed successfully.
NSGY recommended radiation-oncology referral for adjuvant therapy.
The patient had a prolonged hospital course, but is now ready to be discharged home.

## Slide 20
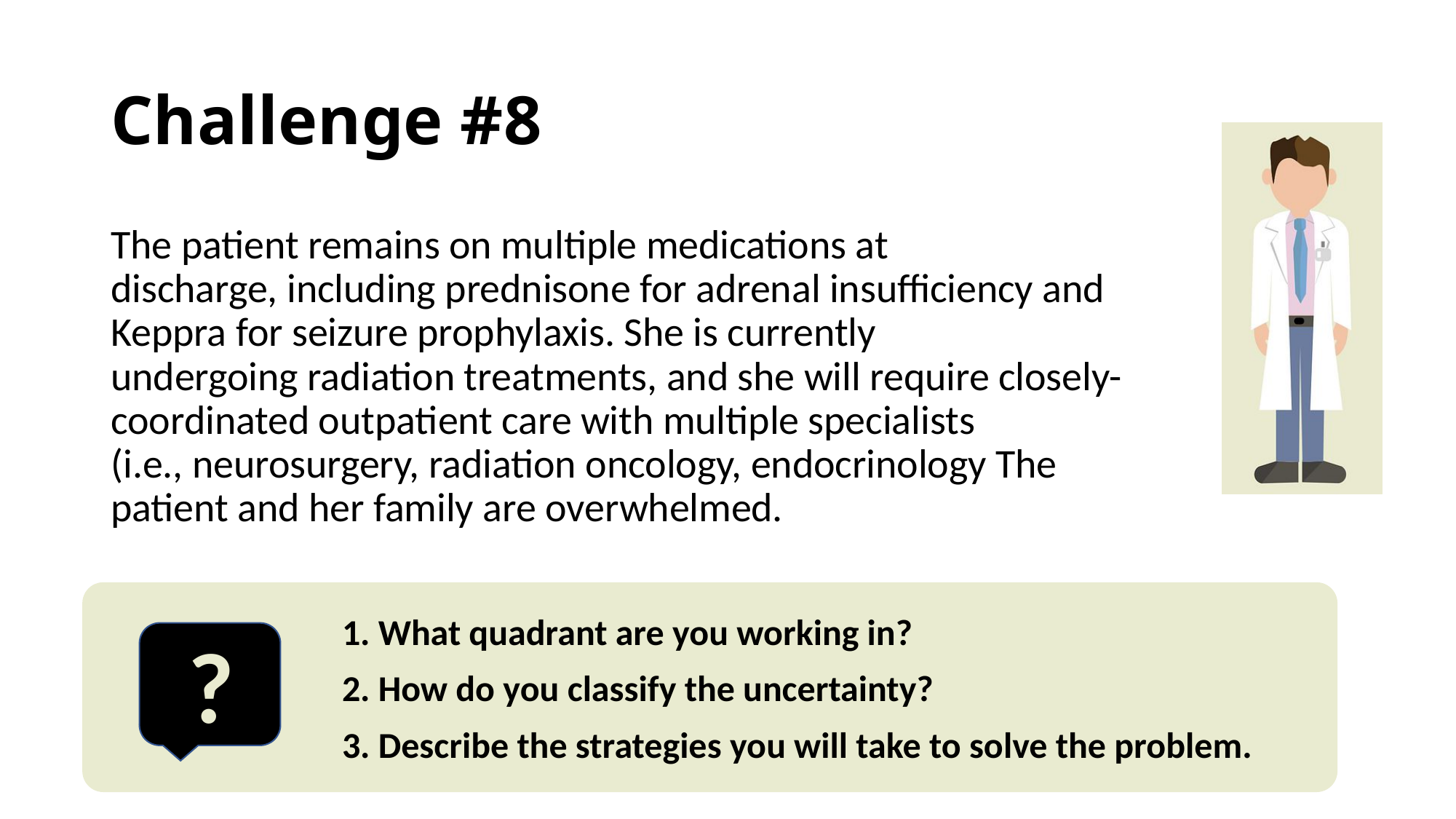

# Challenge #8
The patient remains on multiple medications at discharge, including prednisone for adrenal insufficiency and Keppra for seizure prophylaxis. She is currently undergoing radiation treatments, and she will require closely-coordinated outpatient care with multiple specialists (i.e., neurosurgery, radiation oncology, endocrinology The patient and her family are overwhelmed. ​
1. What quadrant are you working in?
2. How do you classify the uncertainty?
3. Describe the strategies you will take to solve the problem.
?

## Slide 21
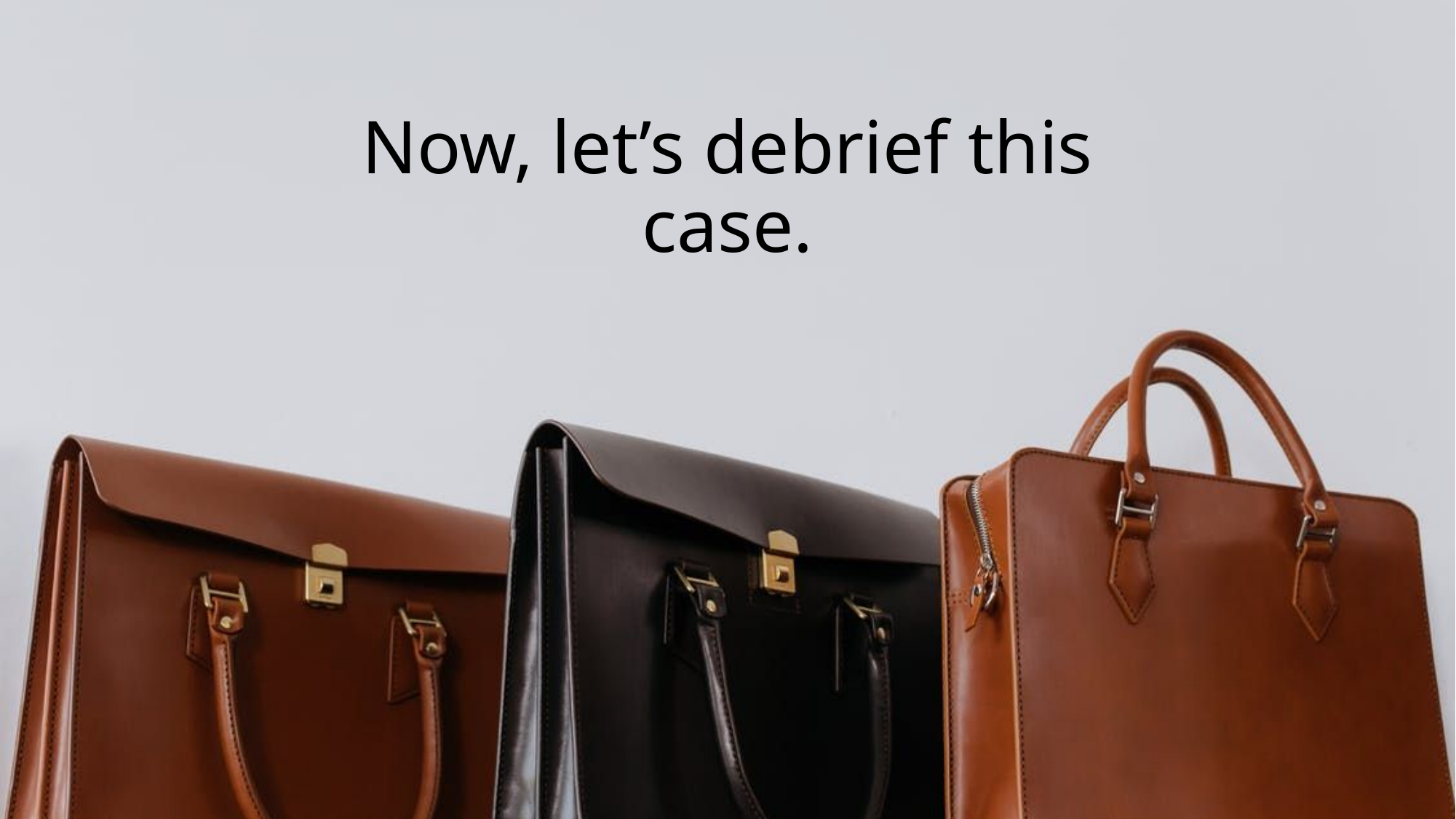

# Now, let’s debrief this case.

## Slide 22
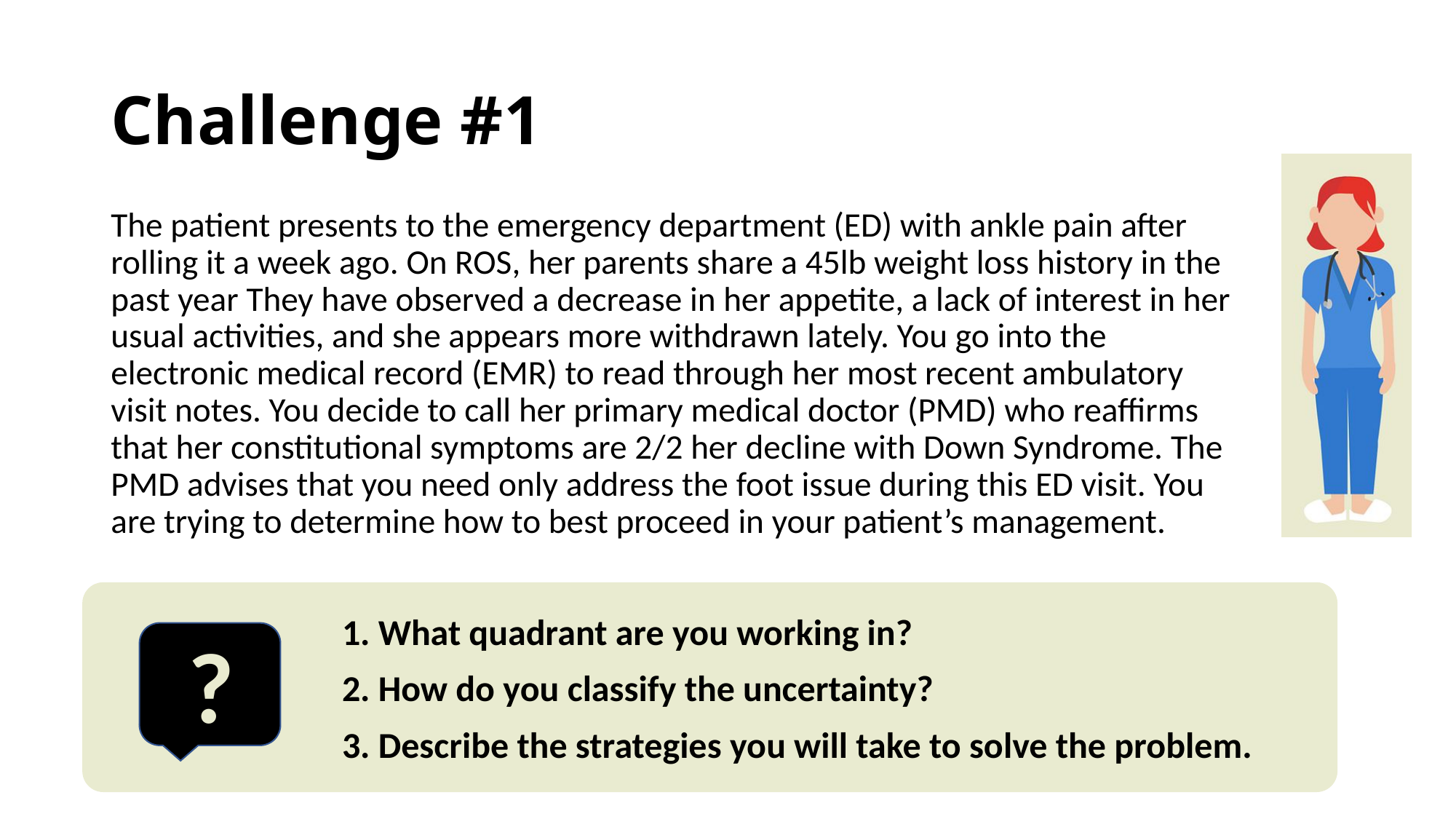

# Challenge #1
The patient presents to the emergency department (ED) with ankle pain after rolling it a week ago. On ROS, her parents share a 45lb weight loss history in the past year They have observed a decrease in her appetite, a lack of interest in her usual activities, and she appears more withdrawn lately. You go into the electronic medical record (EMR) to read through her most recent ambulatory visit notes. You decide to call her primary medical doctor (PMD) who reaffirms that her constitutional symptoms are 2/2 her decline with Down Syndrome. The PMD advises that you need only address the foot issue during this ED visit. You are trying to determine how to best proceed in your patient’s management.
1. What quadrant are you working in?
2. How do you classify the uncertainty?
3. Describe the strategies you will take to solve the problem.
?

## Slide 23
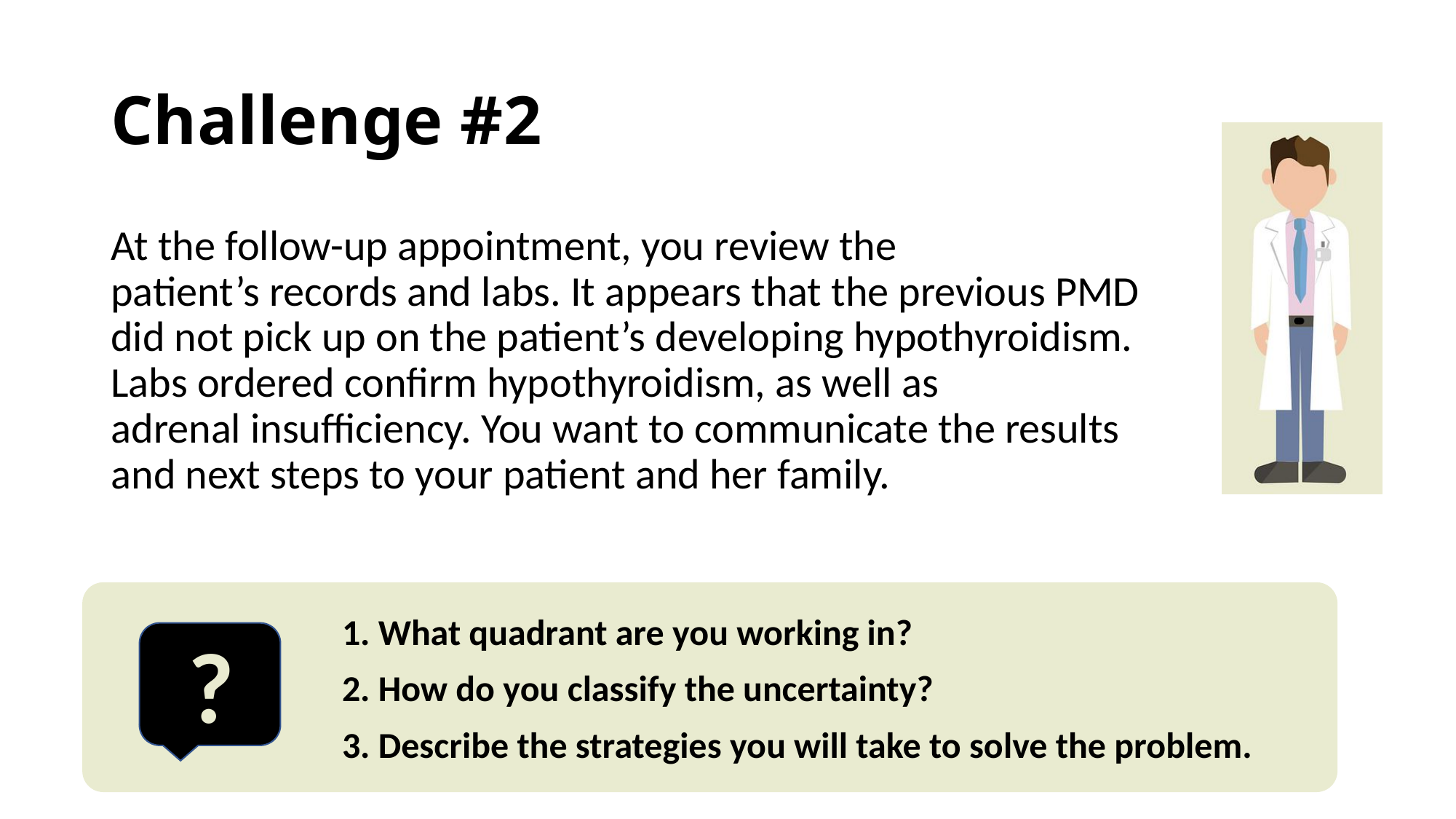

# Challenge #2
At the follow-up appointment, you review the patient’s records and labs. It appears that the previous PMD did not pick up on the patient’s developing hypothyroidism. Labs ordered confirm hypothyroidism, as well as adrenal insufficiency. You want to communicate the results and next steps to your patient and her family. ​
1. What quadrant are you working in?
2. How do you classify the uncertainty?
3. Describe the strategies you will take to solve the problem.
1. What quadrant are you working in?
2. How do you classify the uncertainty?
3. Describe the strategies you will take to solve the problem.
?

## Slide 24
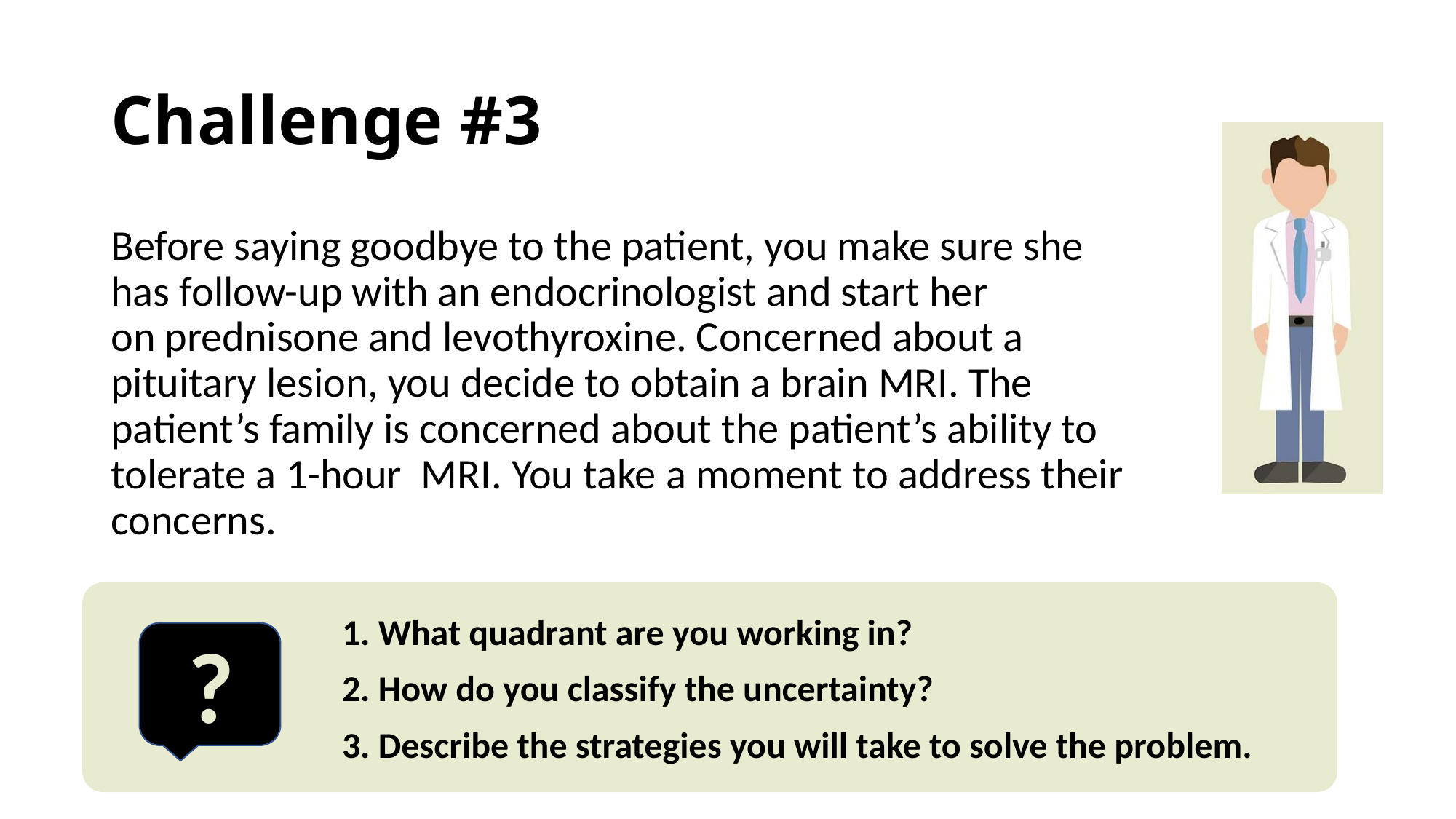

# Challenge #3
Before saying goodbye to the patient, you make sure she has follow-up with an endocrinologist and start her on prednisone and levothyroxine. Concerned about a pituitary lesion, you decide to obtain a brain MRI. The patient’s family is concerned about the patient’s ability to tolerate a 1-hour  MRI. You take a moment to address their concerns.  ​
1. What quadrant are you working in?
2. How do you classify the uncertainty?
3. Describe the strategies you will take to solve the problem.
?

## Slide 25
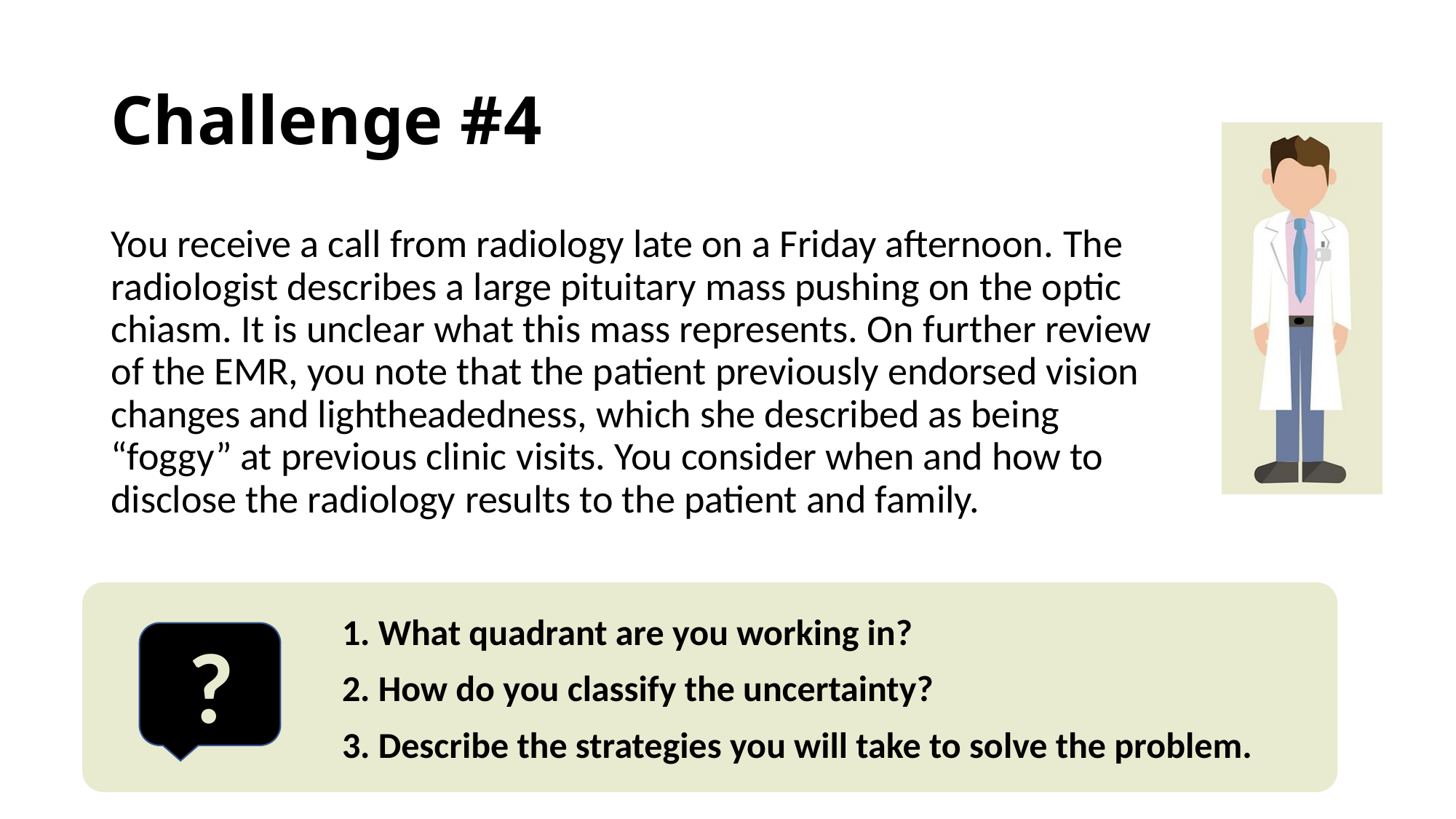

# Challenge #4
You receive a call from radiology late on a Friday afternoon. The radiologist describes a large pituitary mass pushing on the optic chiasm. It is unclear what this mass represents. On further review of the EMR, you note that the patient previously endorsed vision changes and lightheadedness, which she described as being “foggy” at previous clinic visits. You consider when and how to disclose the radiology results to the patient and family.  ​
1. What quadrant are you working in?
2. How do you classify the uncertainty?
3. Describe the strategies you will take to solve the problem.
?

## Slide 26
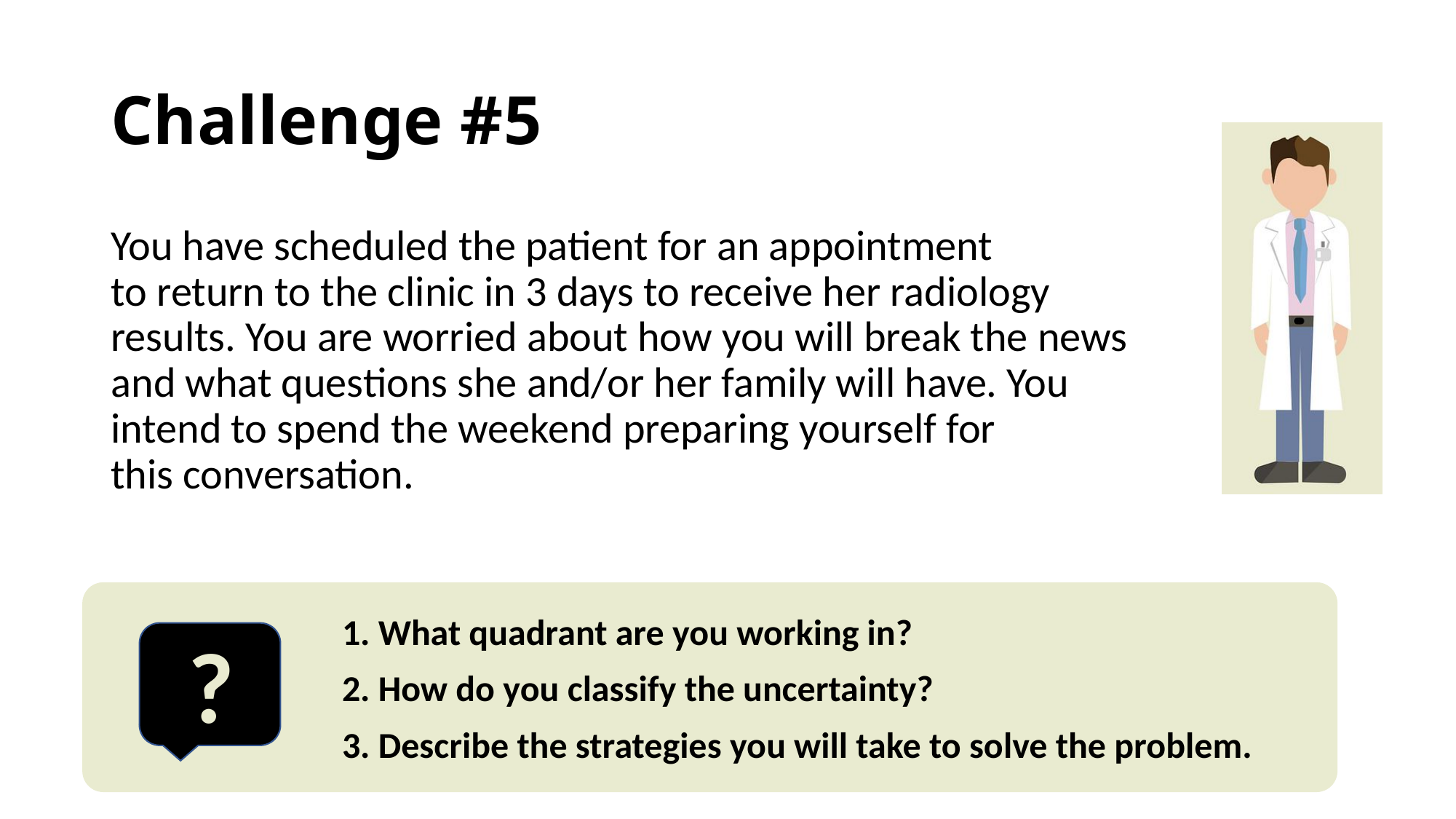

# Challenge #5
You have scheduled the patient for an appointment to return to the clinic in 3 days to receive her radiology results. You are worried about how you will break the news and what questions she and/or her family will have. You intend to spend the weekend preparing yourself for this conversation. ​
1. What quadrant are you working in?
2. How do you classify the uncertainty?
3. Describe the strategies you will take to solve the problem.
?

## Slide 27
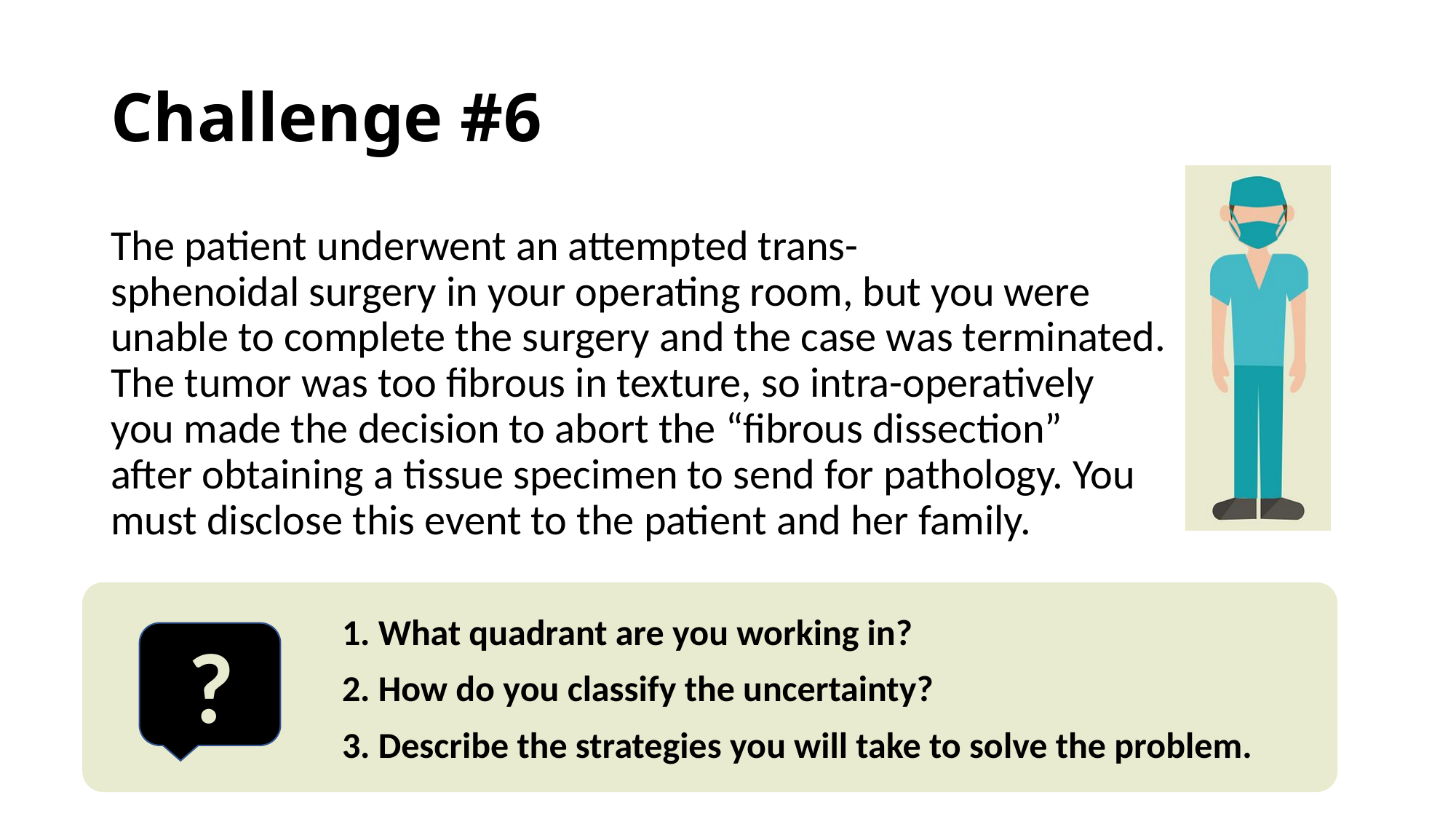

# Challenge #6
The patient underwent an attempted trans-sphenoidal surgery in your operating room, but you were unable to complete the surgery and the case was terminated. The tumor was too fibrous in texture, so intra-operatively you made the decision to abort the “fibrous dissection” after obtaining a tissue specimen to send for pathology. You must disclose this event to the patient and her family.​
1. What quadrant are you working in?
2. How do you classify the uncertainty?
3. Describe the strategies you will take to solve the problem.
?

## Slide 28
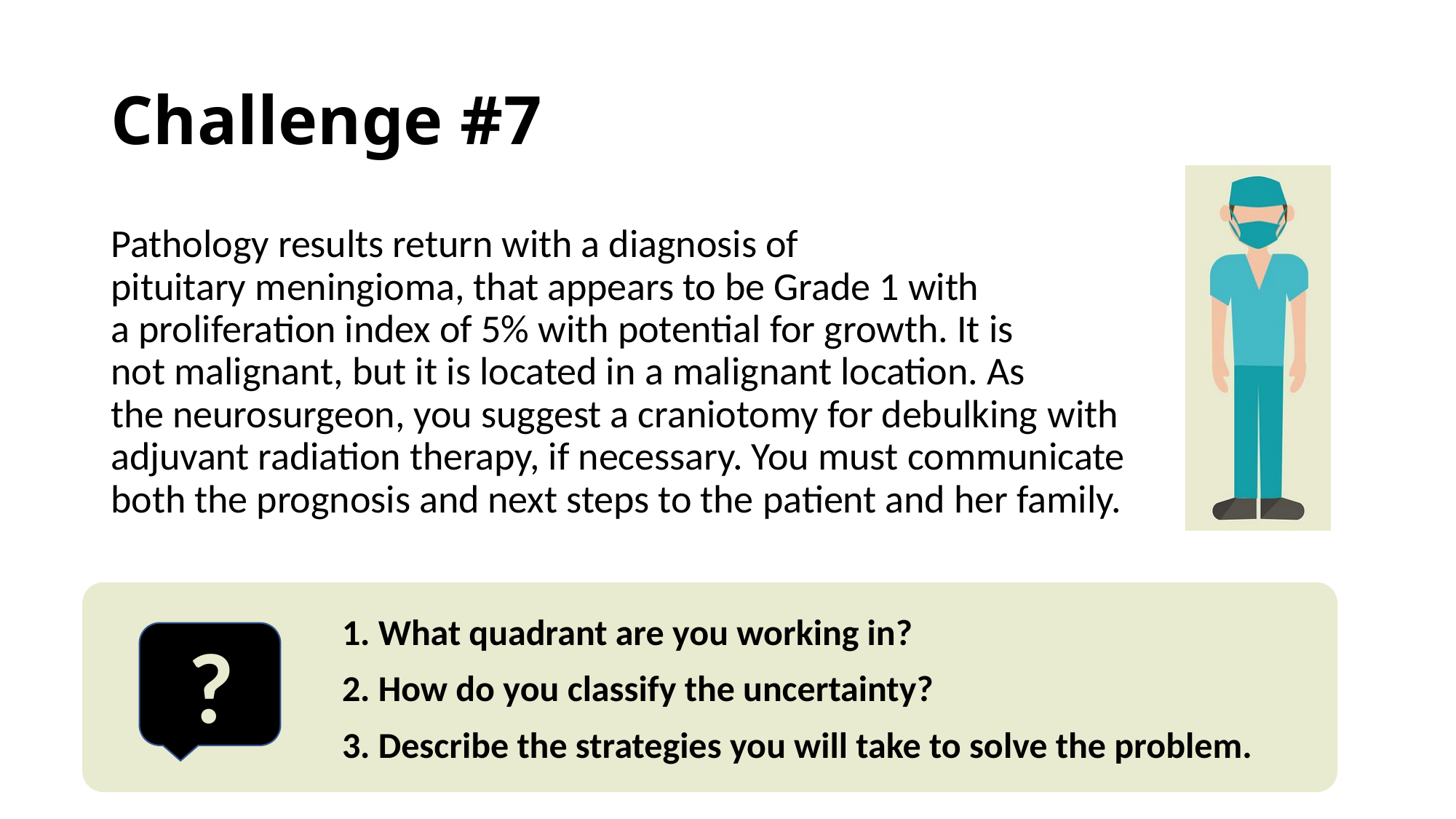

# Challenge #7
Pathology results return with a diagnosis of pituitary meningioma, that appears to be Grade 1 with a proliferation index of 5% with potential for growth. It is not malignant, but it is located in a malignant location. As the neurosurgeon, you suggest a craniotomy for debulking with adjuvant radiation therapy, if necessary. You must communicate both the prognosis and next steps to the patient and her family. ​
1. What quadrant are you working in?
2. How do you classify the uncertainty?
3. Describe the strategies you will take to solve the problem.
?

## Slide 29
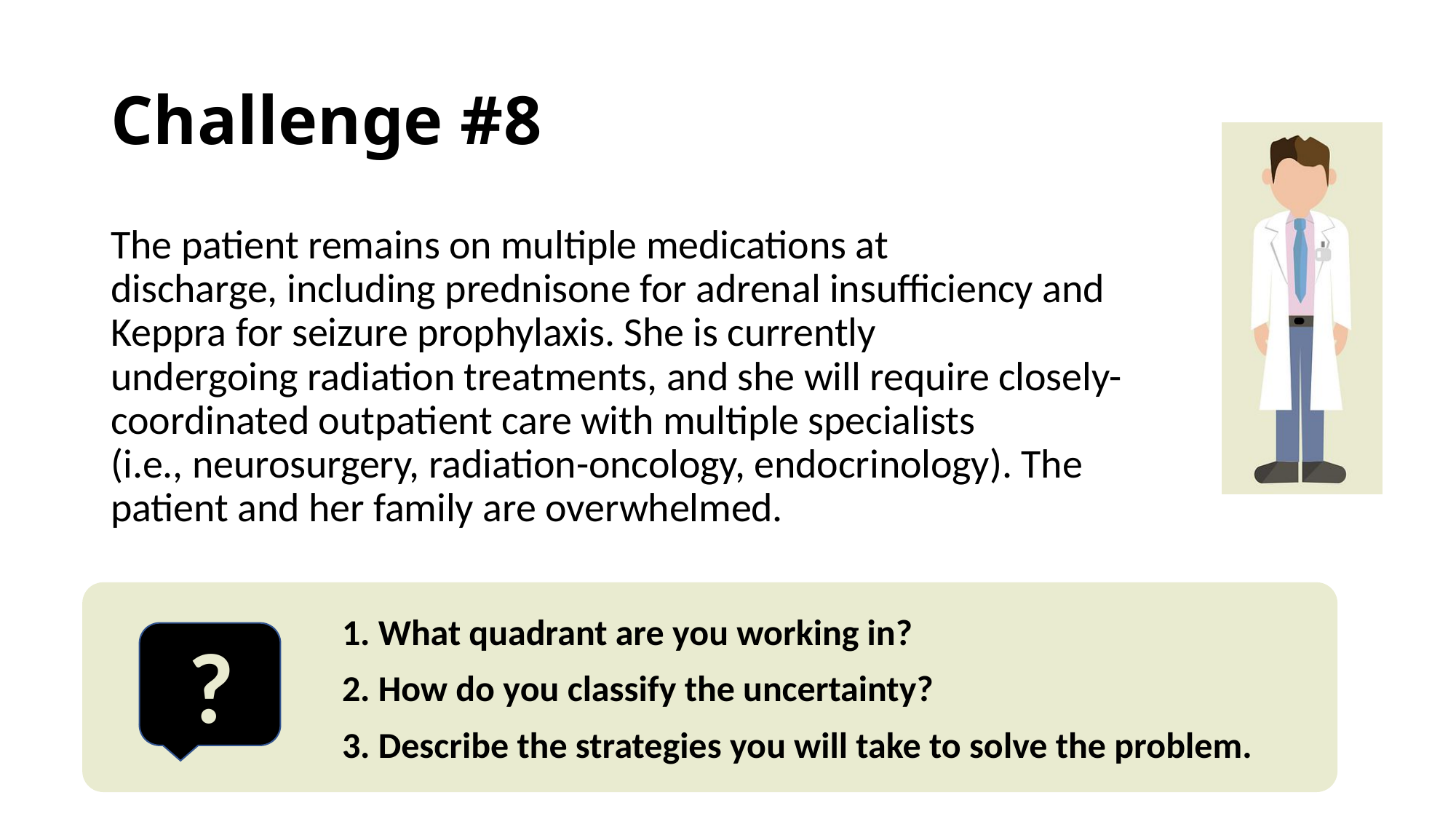

# Challenge #8
The patient remains on multiple medications at discharge, including prednisone for adrenal insufficiency and Keppra for seizure prophylaxis. She is currently undergoing radiation treatments, and she will require closely-coordinated outpatient care with multiple specialists (i.e., neurosurgery, radiation-oncology, endocrinology). The patient and her family are overwhelmed. ​
1. What quadrant are you working in?
2. How do you classify the uncertainty?
3. Describe the strategies you will take to solve the problem.
?

## Slide 30
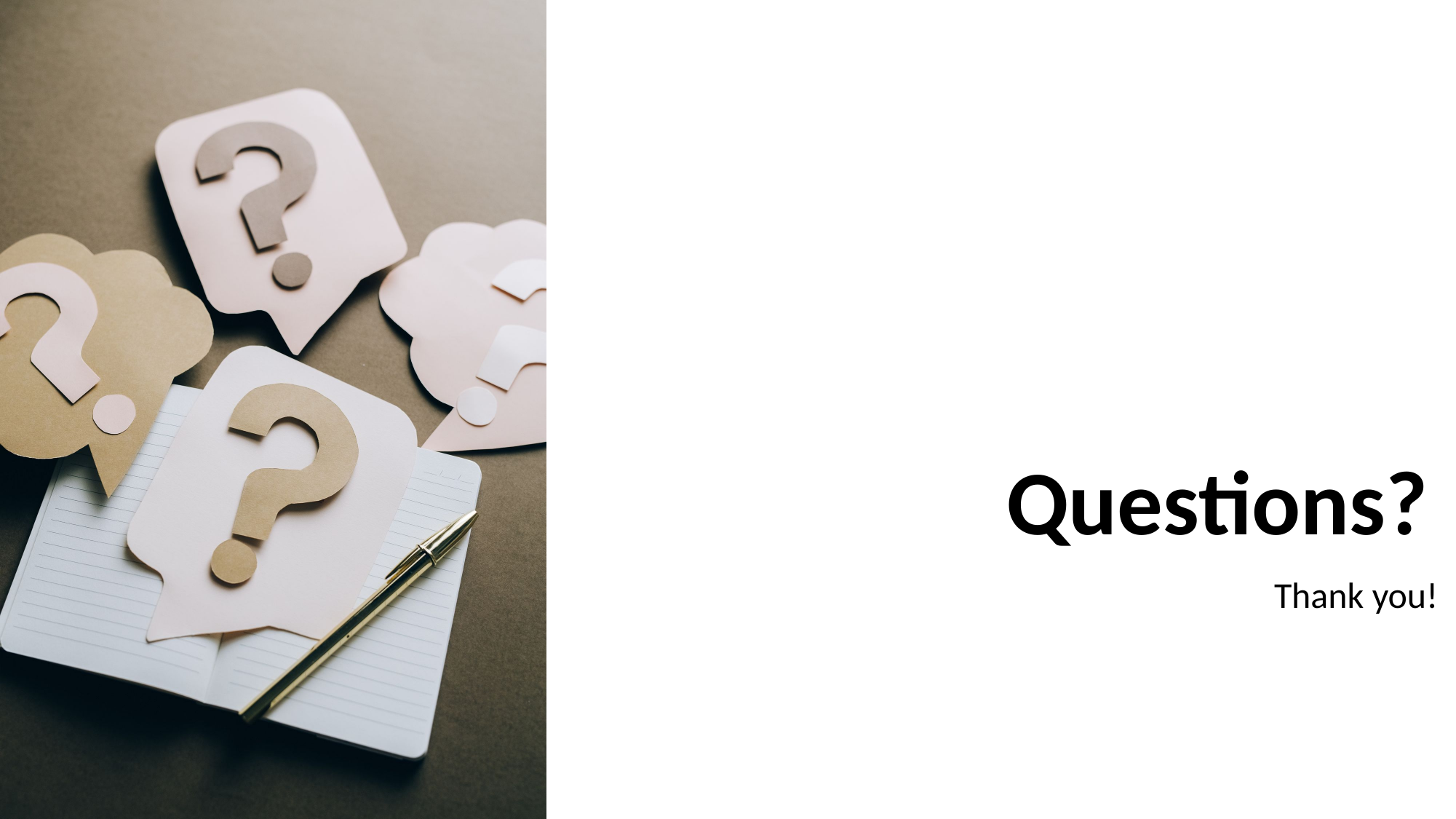

# Questions?
Thank you!
