# Supplementary material for: Navigating Uncertainty in Clinical Practice: A Workshop to Prepare Medical Students to Problem-Solve During Complex Clinical Challenges
Source: MedEdPORTAL. 2023 Aug 9;19:11334. doi: 10.15766/mep_2374-8265.11334 (PMC10409886; doi:10.15766/mep_2374-8265.11334)
Supplement: Supplementary file 1 — Case Slides.pptxStudent Instructions.docxUncertainty Didactic Slides.pptxFacilitator Instructions.docxPostsession Survey.docx [file mep_2374-8265.11334-s001.zip › B. Student Instructions.docx]

**Student Instructions**

Navigating Uncertainty in Clinical Practice:

A Workshop to Prepare Medical Students to Problem-Solve During Complex Clinical Challenges

**Classification of Uncertainty in Clinical Practice:**

 
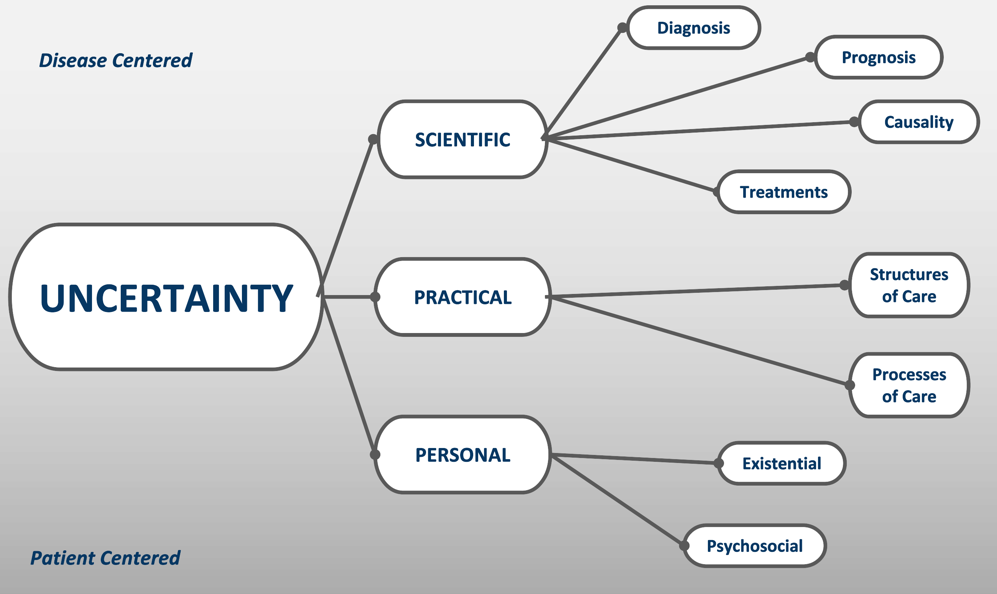


Adapted from: Han PK, Klein WM, Arora NK. Varieties of uncertainty in health care: a conceptual taxonomy. Med Decis Making. 2011;31(6):828-838. doi:10.1177/0272989x11393976

**Cynefin Framework:**


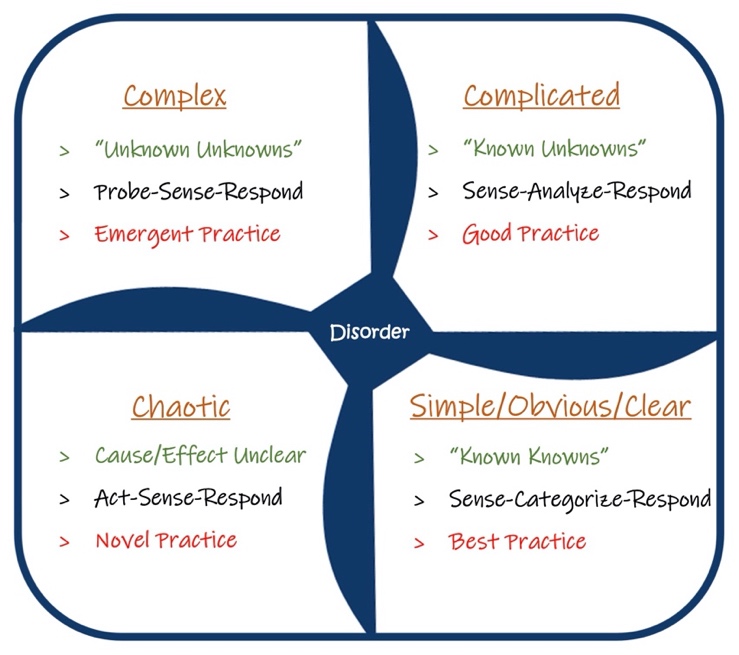


The Cynefin framework for sensemaking. Source: Papanagnou D, Jaffe R, Ziring D. Highlighting a curricular need: Uncertainty, COVID-19, and health systems science. Health Sci Rep. 2021;4:e363. Open access permission granted from Creative Commons. https://creativecommons.org/ licenses/by/4.0/legalcode. Accessed May 19, 2022.

**Case Introduction:**

**
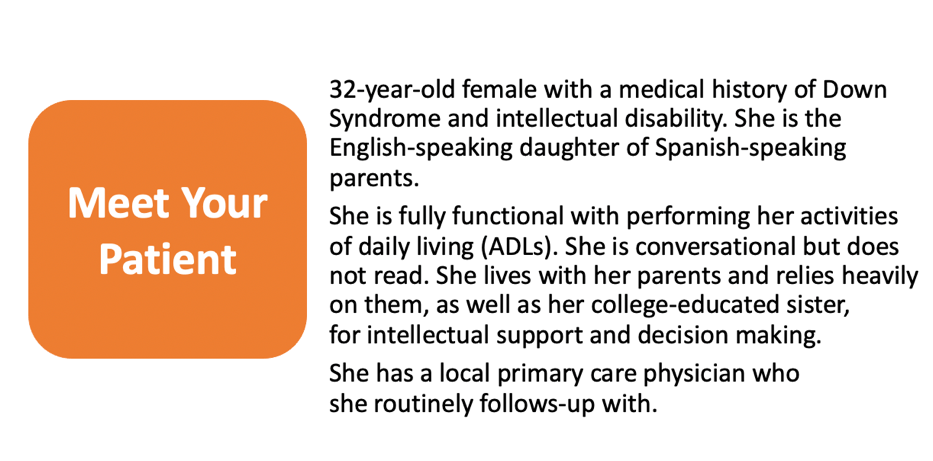
**

**Challenge Point Instructions:**

**
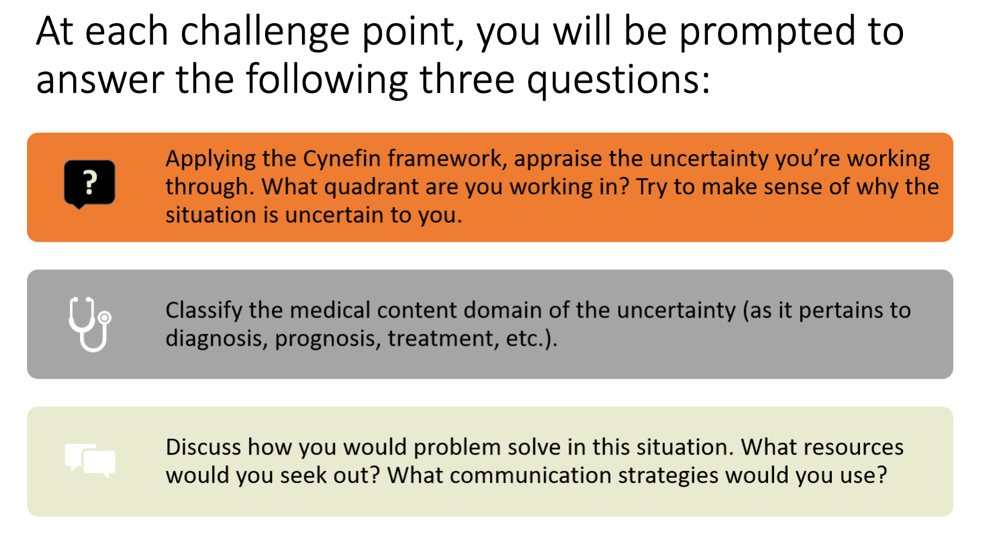
**
