# Supplementary material for: Navigating Uncertainty in Clinical Practice: A Workshop to Prepare Medical Students to Problem-Solve During Complex Clinical Challenges
Source: MedEdPORTAL. 2023 Aug 9;19:11334. doi: 10.15766/mep_2374-8265.11334 (PMC10409886; doi:10.15766/mep_2374-8265.11334)
Supplement: Supplementary file 1 — Case Slides.pptxStudent Instructions.docxUncertainty Didactic Slides.pptxFacilitator Instructions.docxPostsession Survey.docx [file mep_2374-8265.11334-s001.zip › C. Uncertainty Didactic Slides.pptx]

## Slide 1
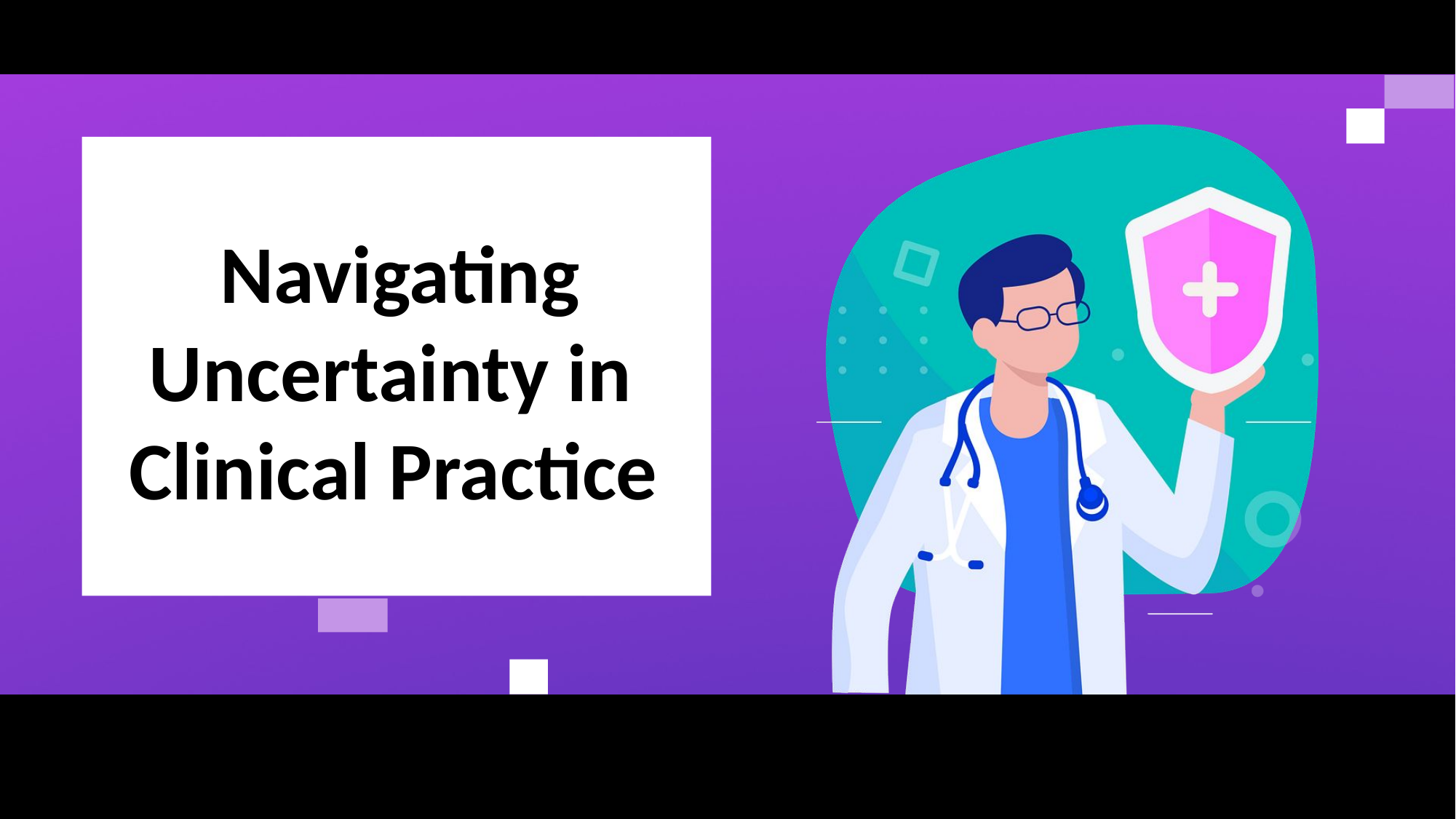

Navigating Uncertainty in
Clinical Practice

## Slide 2
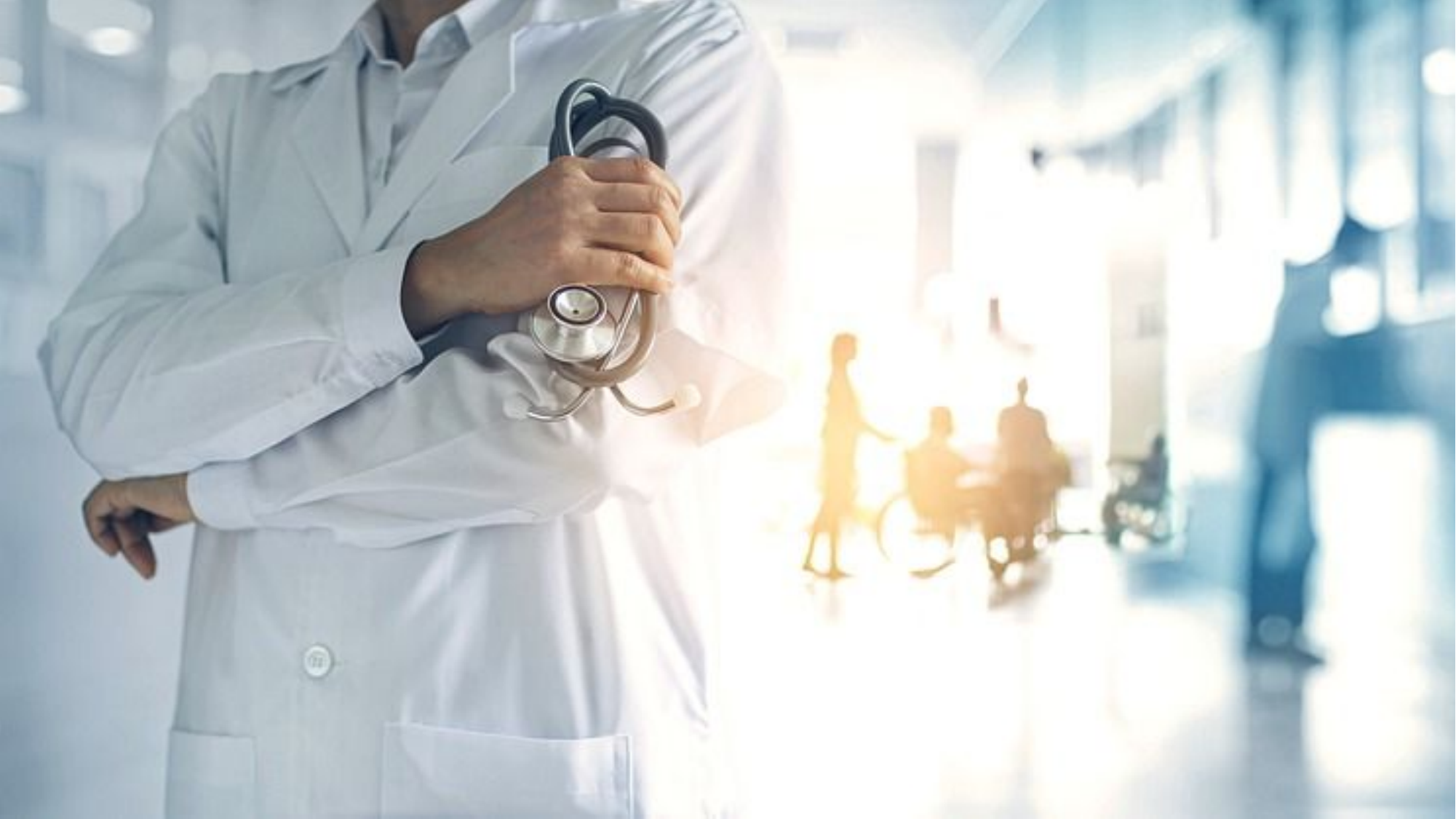

## Slide 3
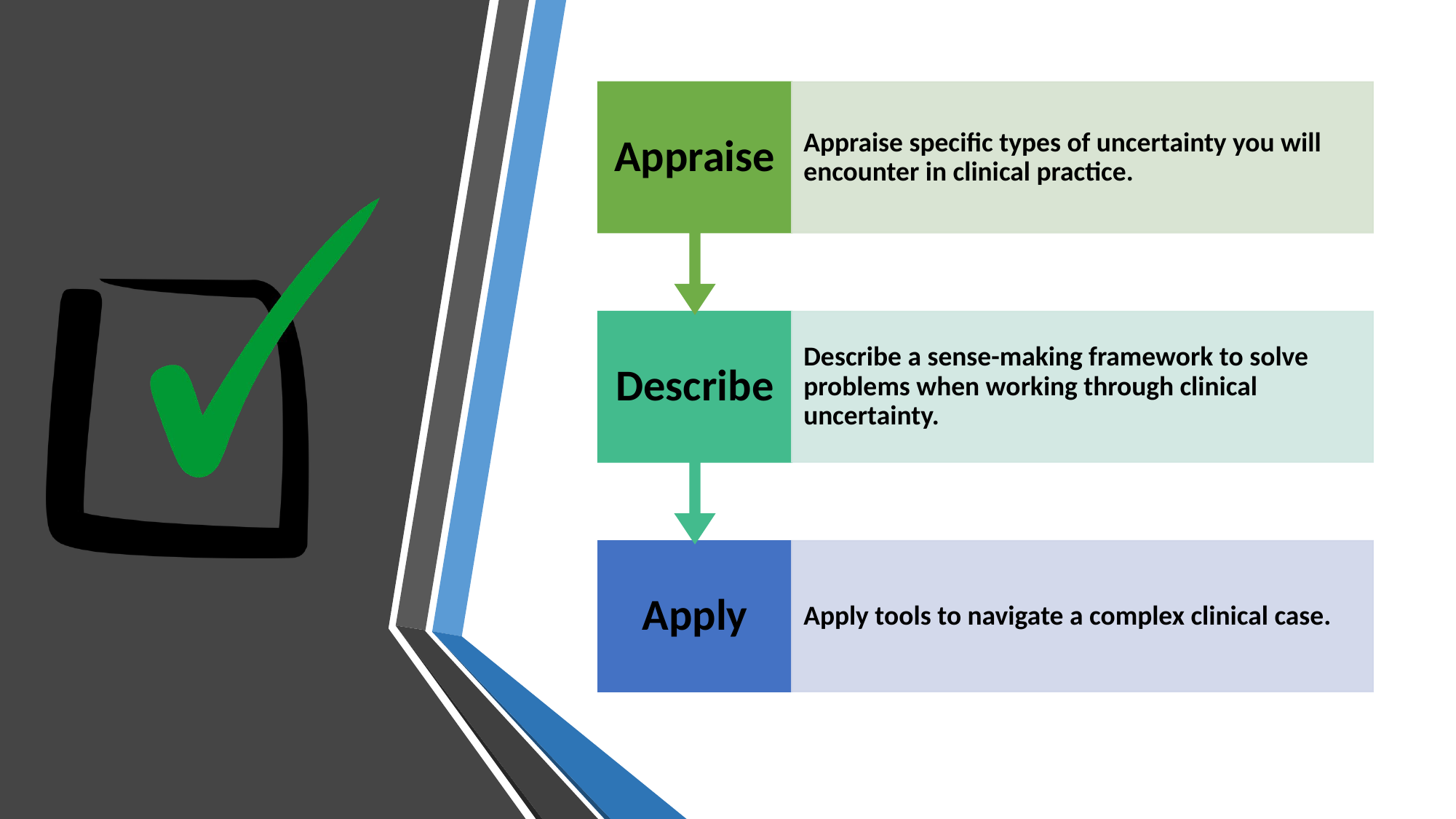

## Slide 4
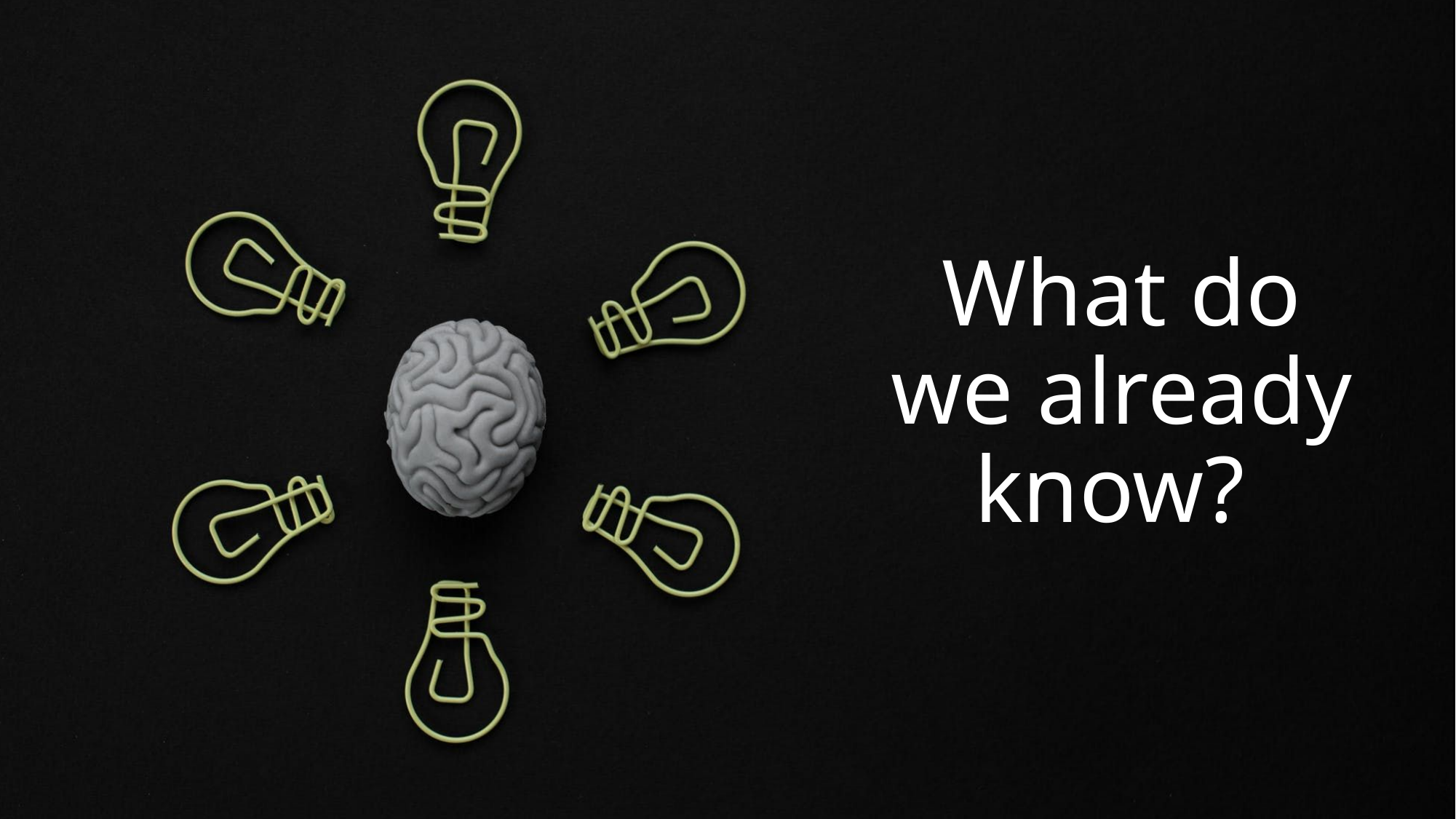

# What do we already know?

## Slide 5
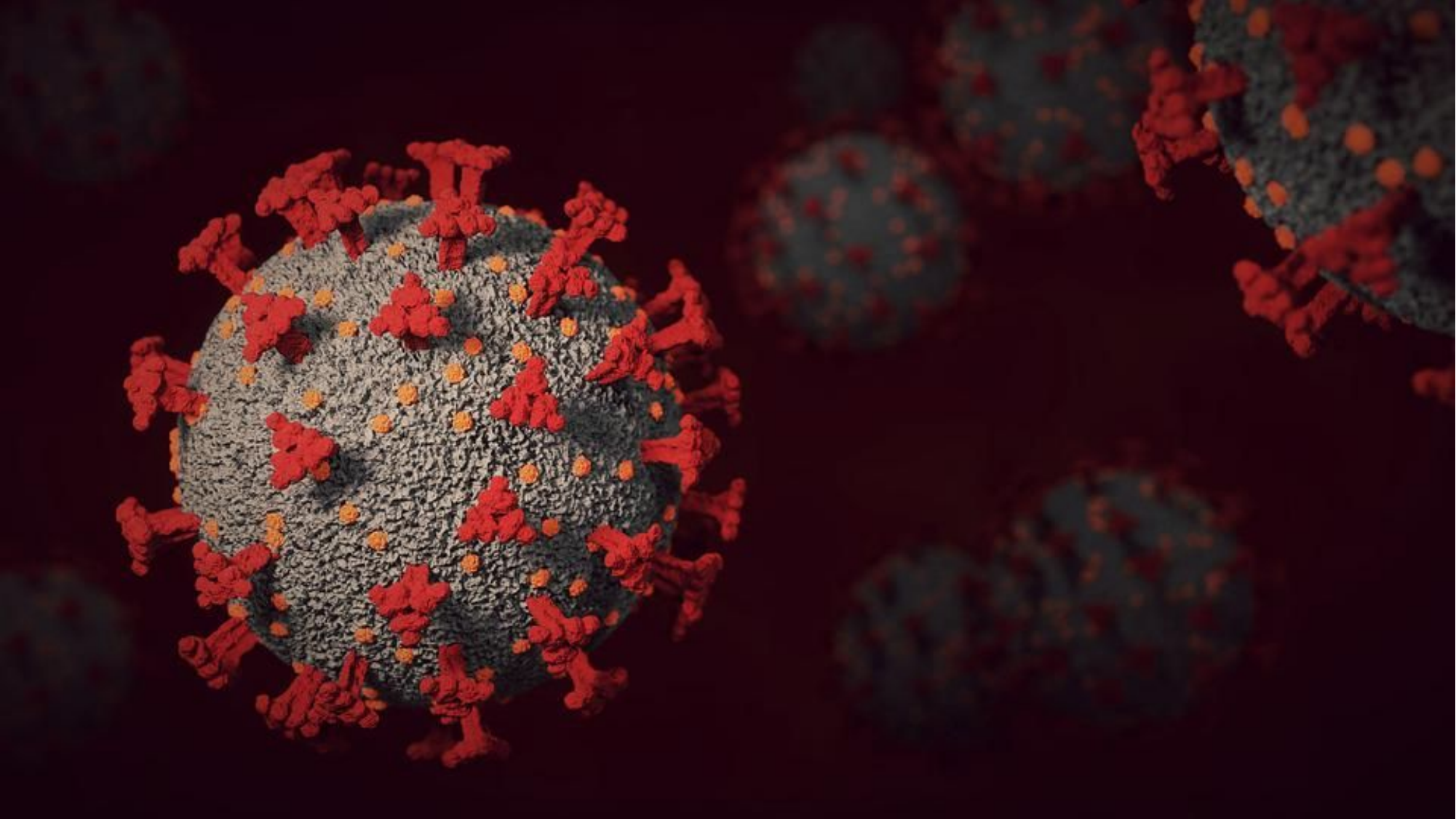

## Slide 6
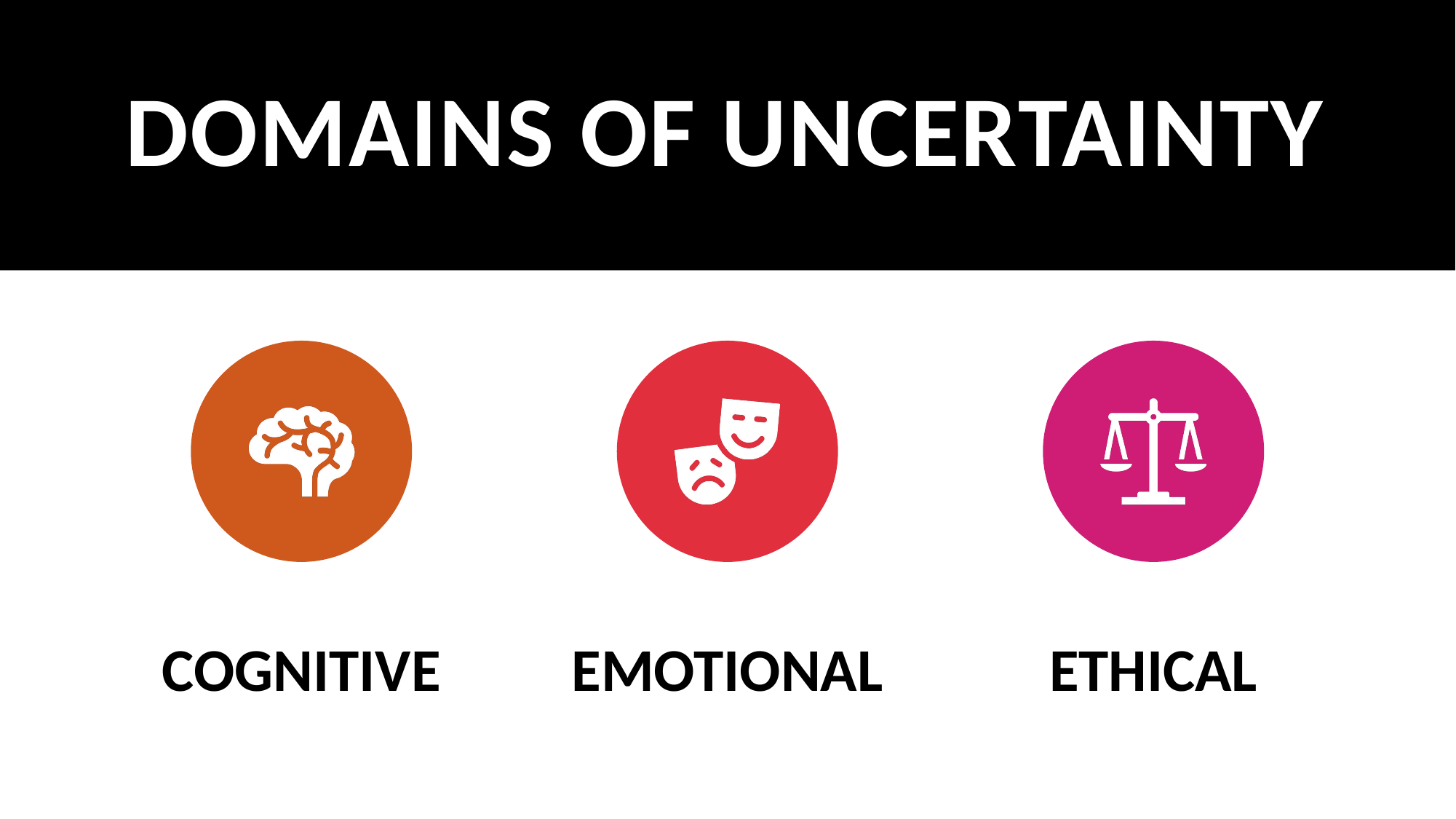

# Domains of Uncertainty

## Slide 7
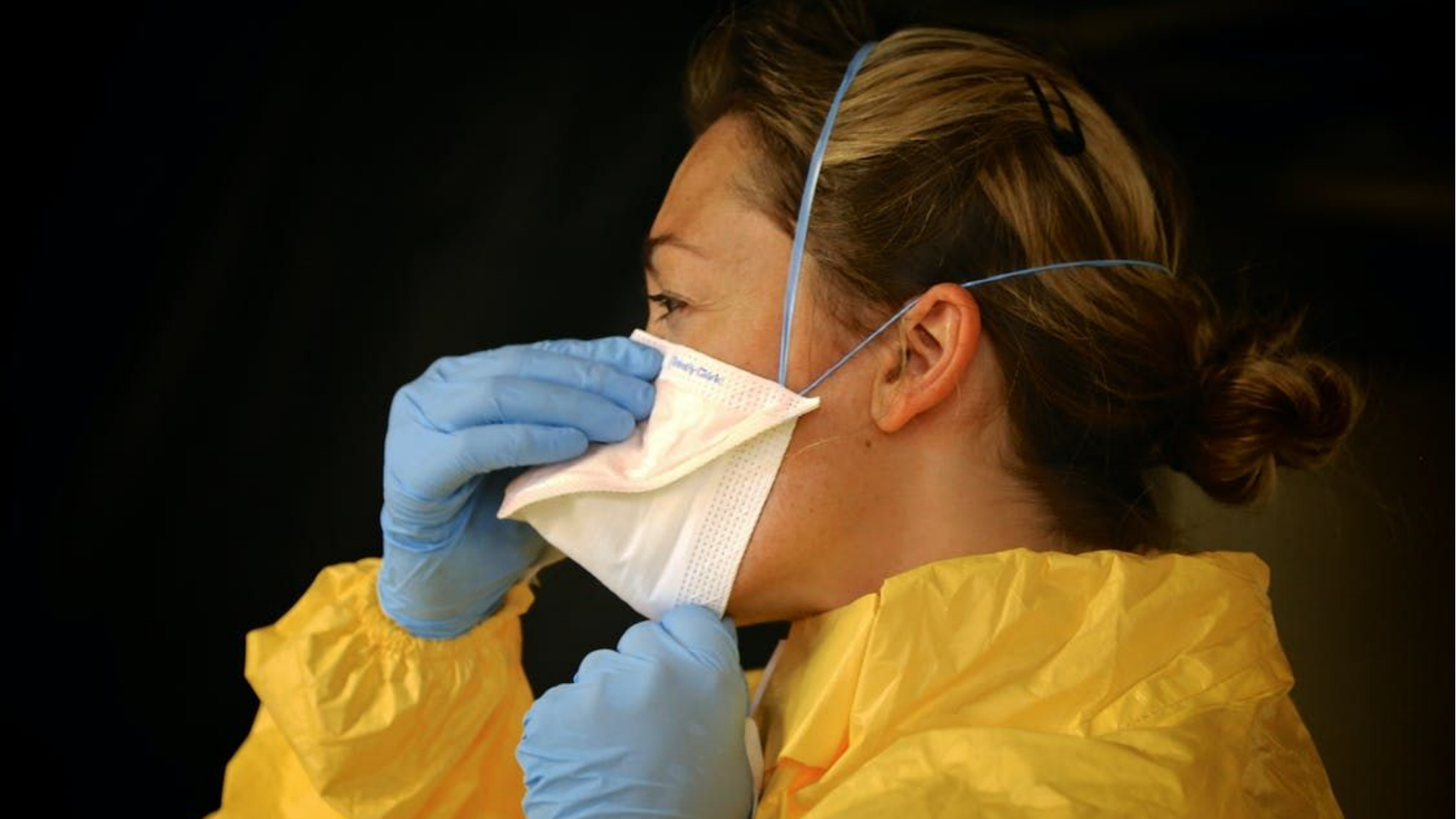

## Slide 8
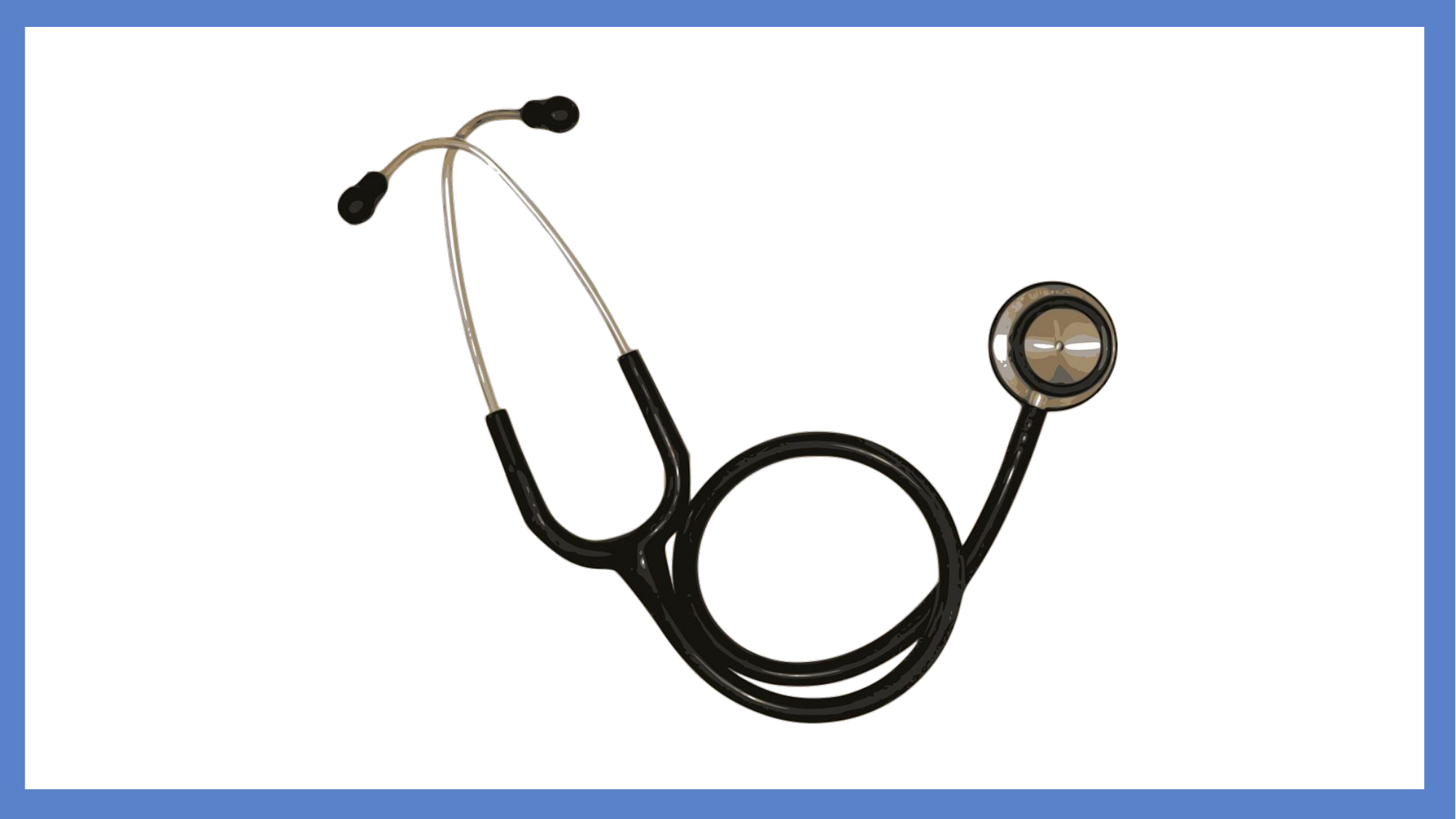

## Slide 9
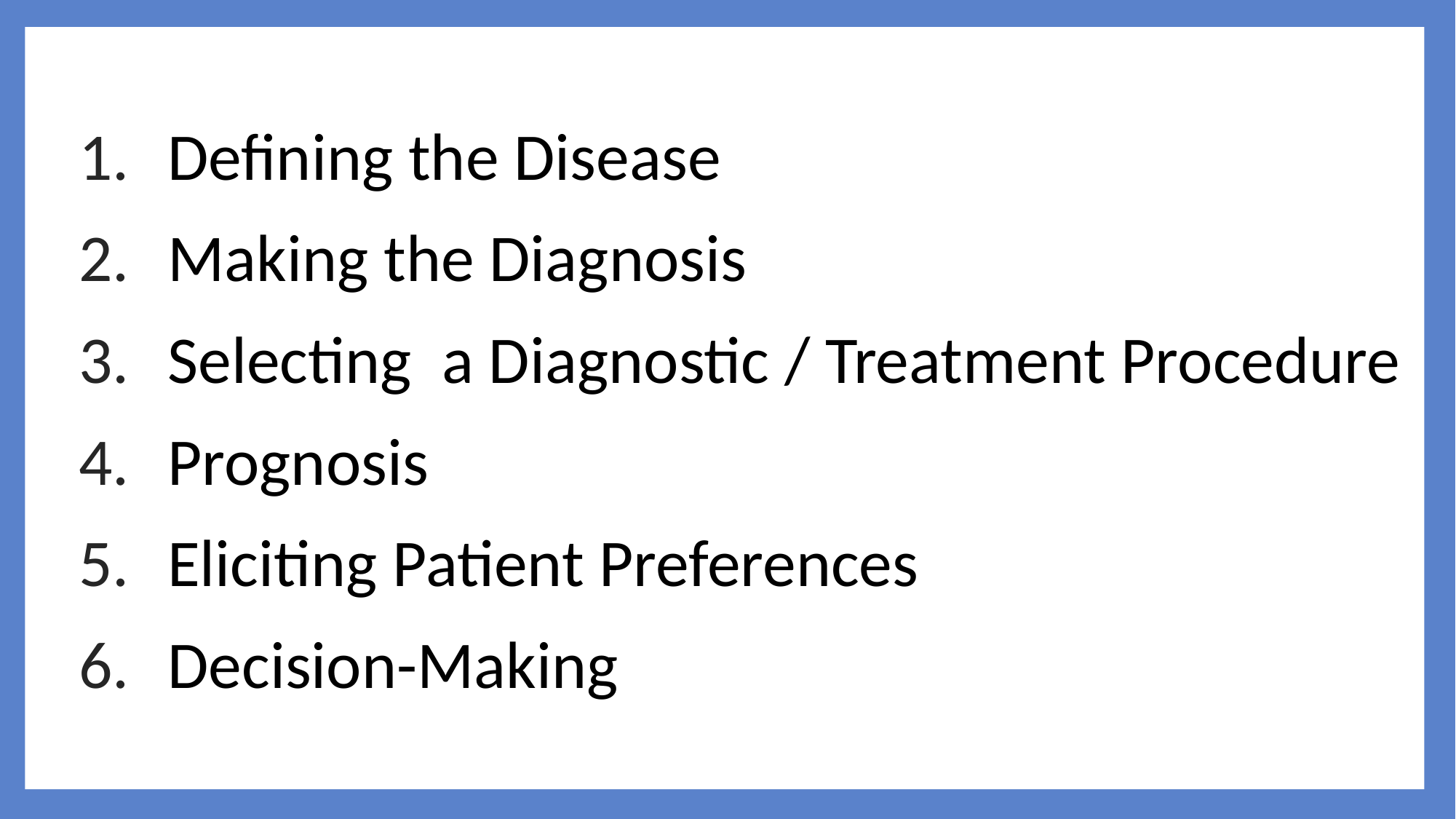

Defining the Disease
Making the Diagnosis
Selecting a Diagnostic / Treatment Procedure
Prognosis
Eliciting Patient Preferences
Decision-Making

## Slide 10
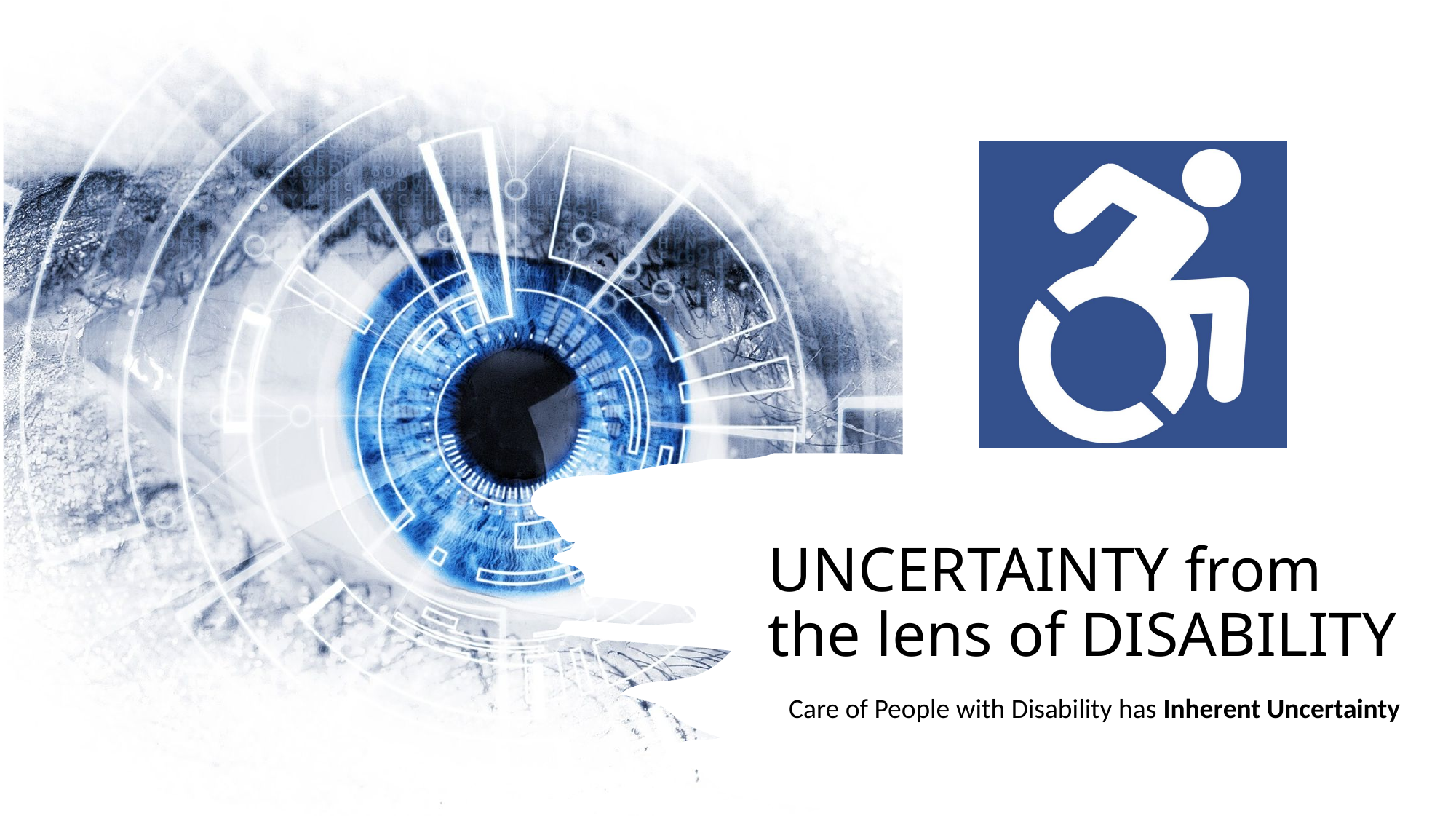

# UNCERTAINTY from the lens of DISABILITY
Care of People with Disability has Inherent Uncertainty

## Slide 11
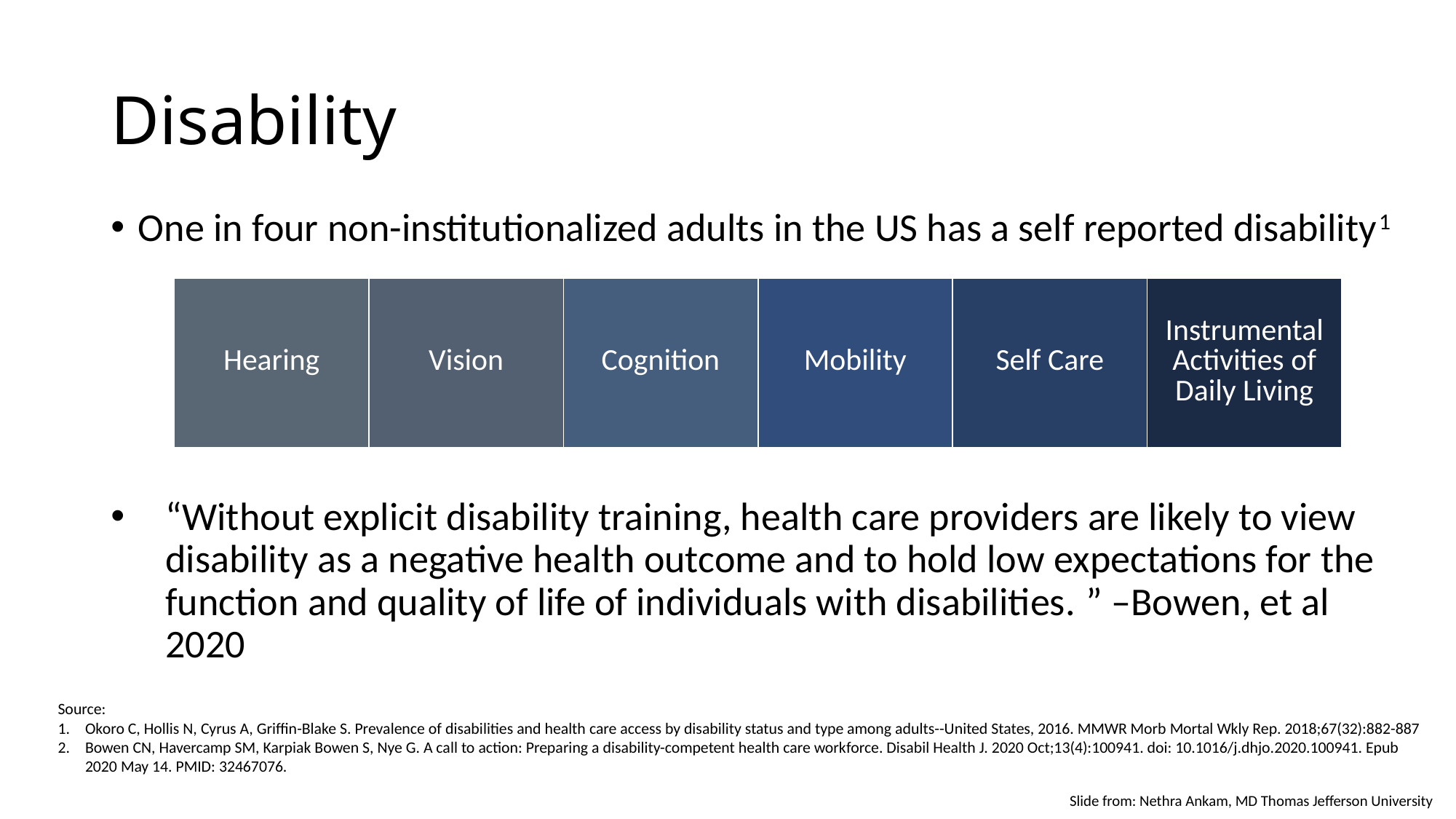

# Disability
One in four non-institutionalized adults in the US has a self reported disability1
“Without explicit disability training, health care providers are likely to view disability as a negative health outcome and to hold low expectations for the function and quality of life of individuals with disabilities. ” –Bowen, et al 2020
| Hearing | Vision | Cognition | Mobility | Self Care | Instrumental Activities of Daily Living |
| --- | --- | --- | --- | --- | --- |
Source:
Okoro C, Hollis N, Cyrus A, Griffin-Blake S. Prevalence of disabilities and health care access by disability status and type among adults--United States, 2016. MMWR Morb Mortal Wkly Rep. 2018;67(32):882-887
Bowen CN, Havercamp SM, Karpiak Bowen S, Nye G. A call to action: Preparing a disability-competent health care workforce. Disabil Health J. 2020 Oct;13(4):100941. doi: 10.1016/j.dhjo.2020.100941. Epub 2020 May 14. PMID: 32467076.
Slide from: Nethra Ankam, MD Thomas Jefferson University

## Slide 12
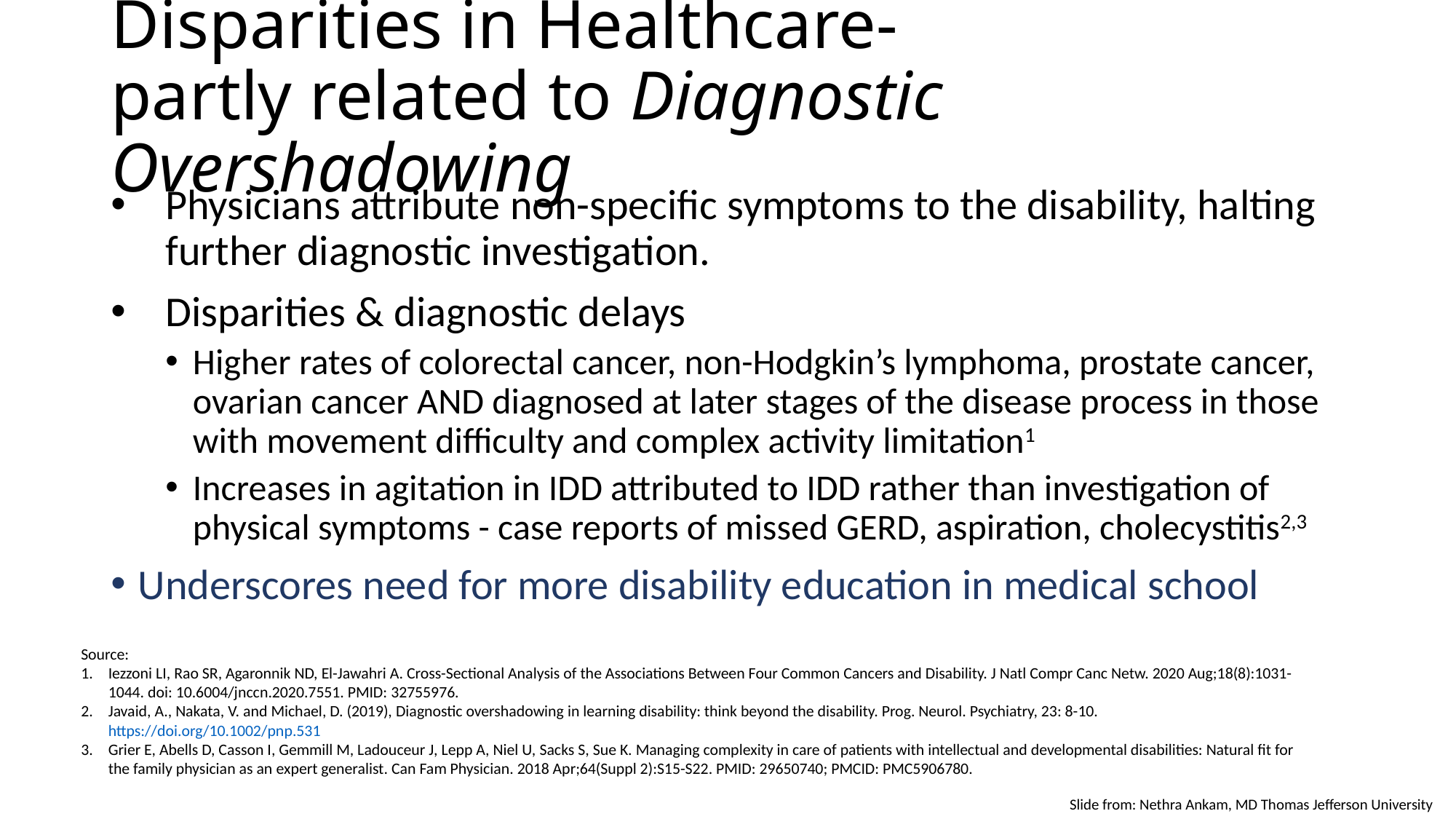

# Disparities in Healthcare- partly related to Diagnostic Overshadowing
Physicians attribute non-specific symptoms to the disability, halting further diagnostic investigation.
Disparities & diagnostic delays
Higher rates of colorectal cancer, non-Hodgkin’s lymphoma, prostate cancer, ovarian cancer AND diagnosed at later stages of the disease process in those with movement difficulty and complex activity limitation1
Increases in agitation in IDD attributed to IDD rather than investigation of physical symptoms - case reports of missed GERD, aspiration, cholecystitis2,3
Underscores need for more disability education in medical school
Source:
Iezzoni LI, Rao SR, Agaronnik ND, El-Jawahri A. Cross-Sectional Analysis of the Associations Between Four Common Cancers and Disability. J Natl Compr Canc Netw. 2020 Aug;18(8):1031-1044. doi: 10.6004/jnccn.2020.7551. PMID: 32755976.
Javaid, A., Nakata, V. and Michael, D. (2019), Diagnostic overshadowing in learning disability: think beyond the disability. Prog. Neurol. Psychiatry, 23: 8-10. https://doi.org/10.1002/pnp.531
Grier E, Abells D, Casson I, Gemmill M, Ladouceur J, Lepp A, Niel U, Sacks S, Sue K. Managing complexity in care of patients with intellectual and developmental disabilities: Natural fit for the family physician as an expert generalist. Can Fam Physician. 2018 Apr;64(Suppl 2):S15-S22. PMID: 29650740; PMCID: PMC5906780.
Slide from: Nethra Ankam, MD Thomas Jefferson University

## Slide 13
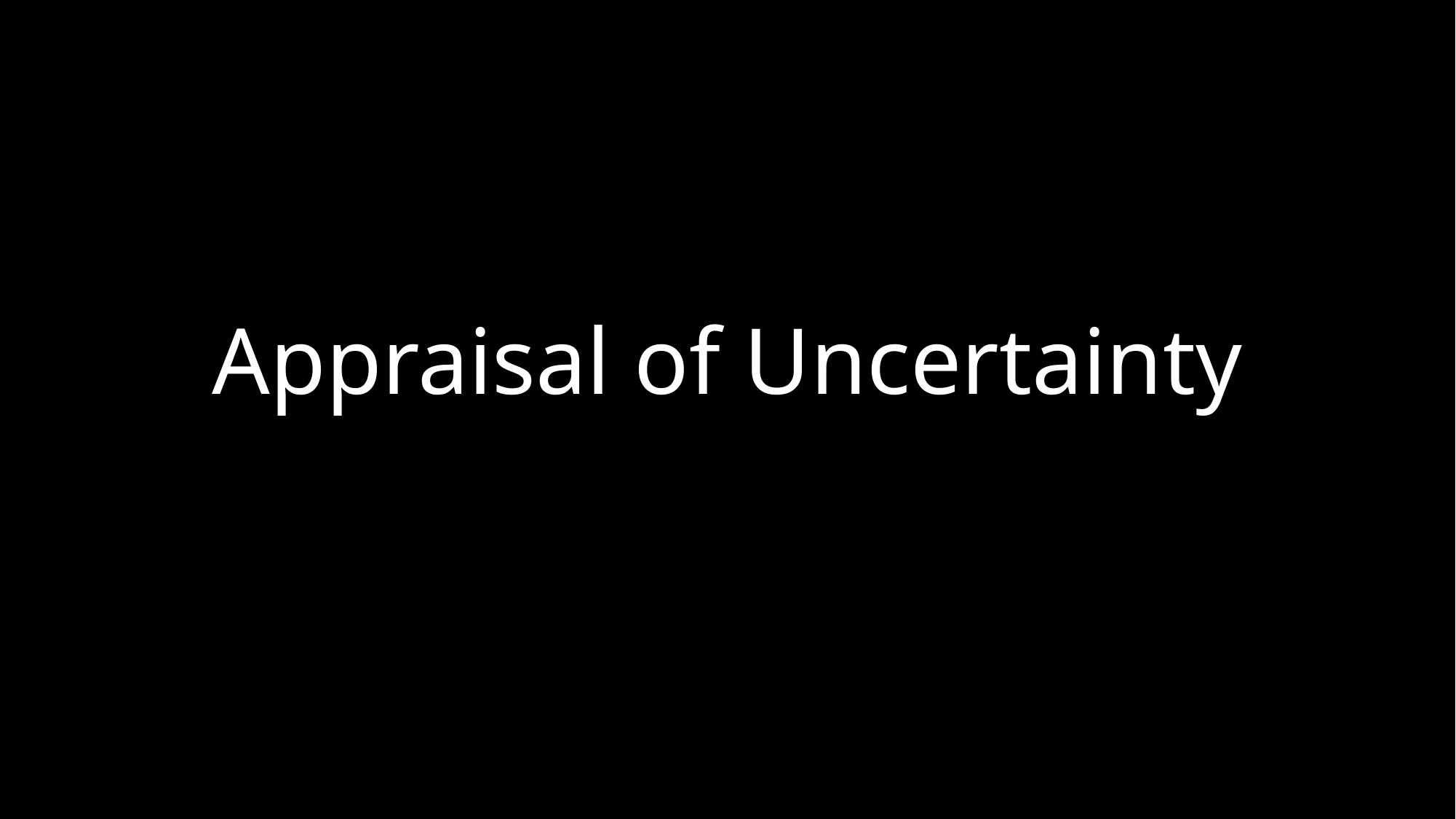

# Appraisal of Uncertainty

## Slide 14
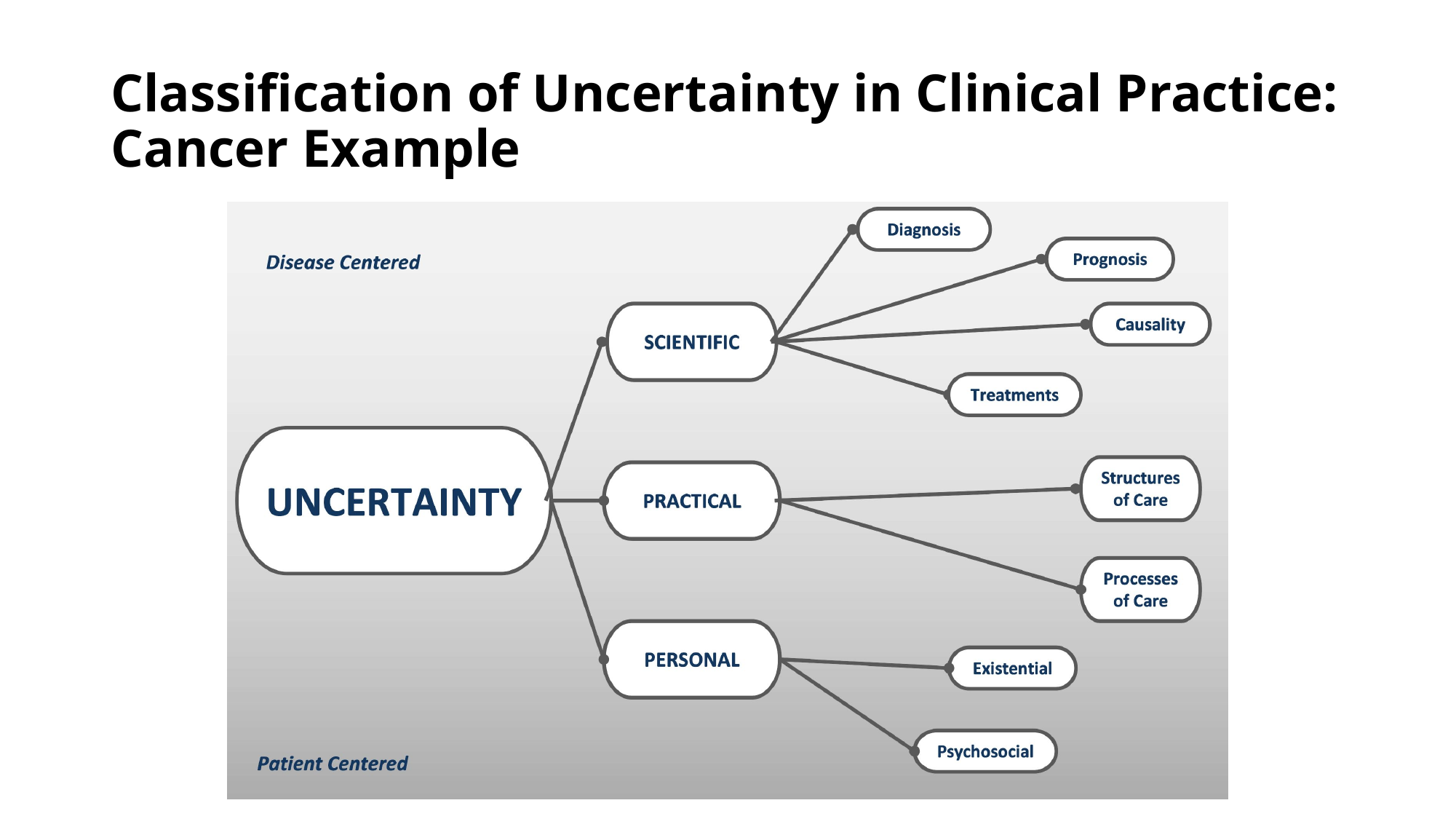

# Classification of Uncertainty in Clinical Practice: Cancer Example

## Slide 15
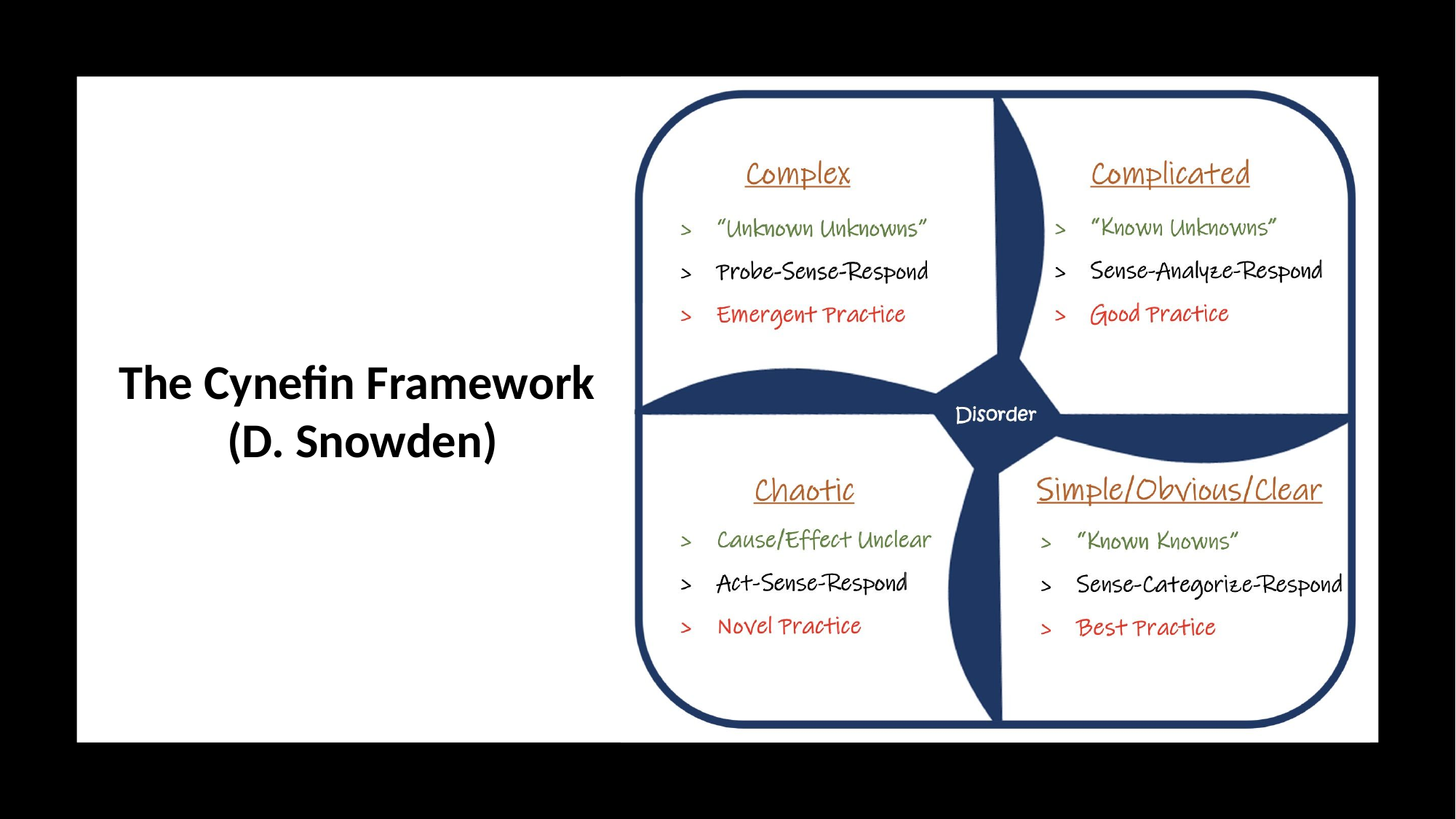

The Cynefin Framework
(D. Snowden)

## Slide 16
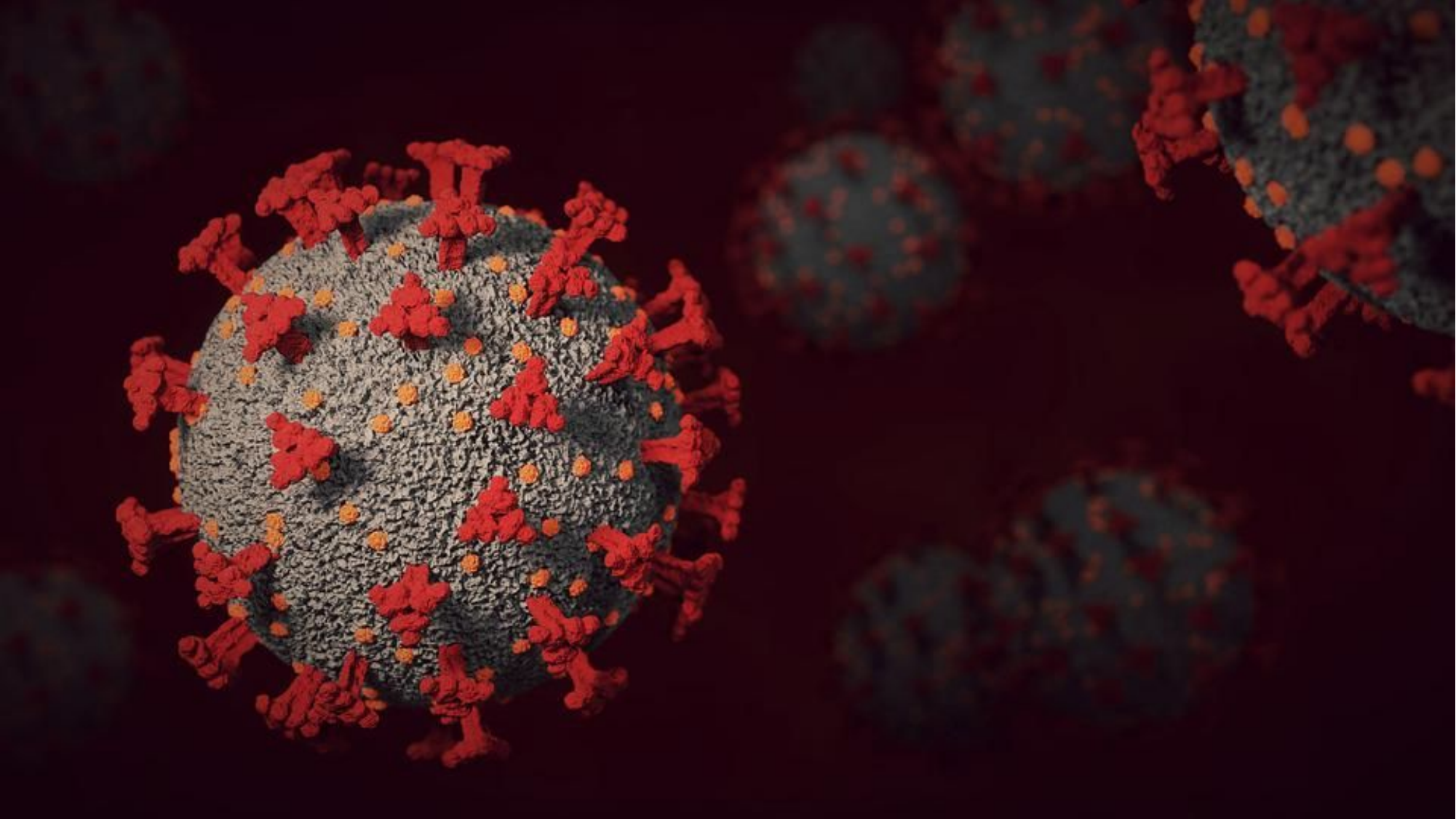

## Slide 17
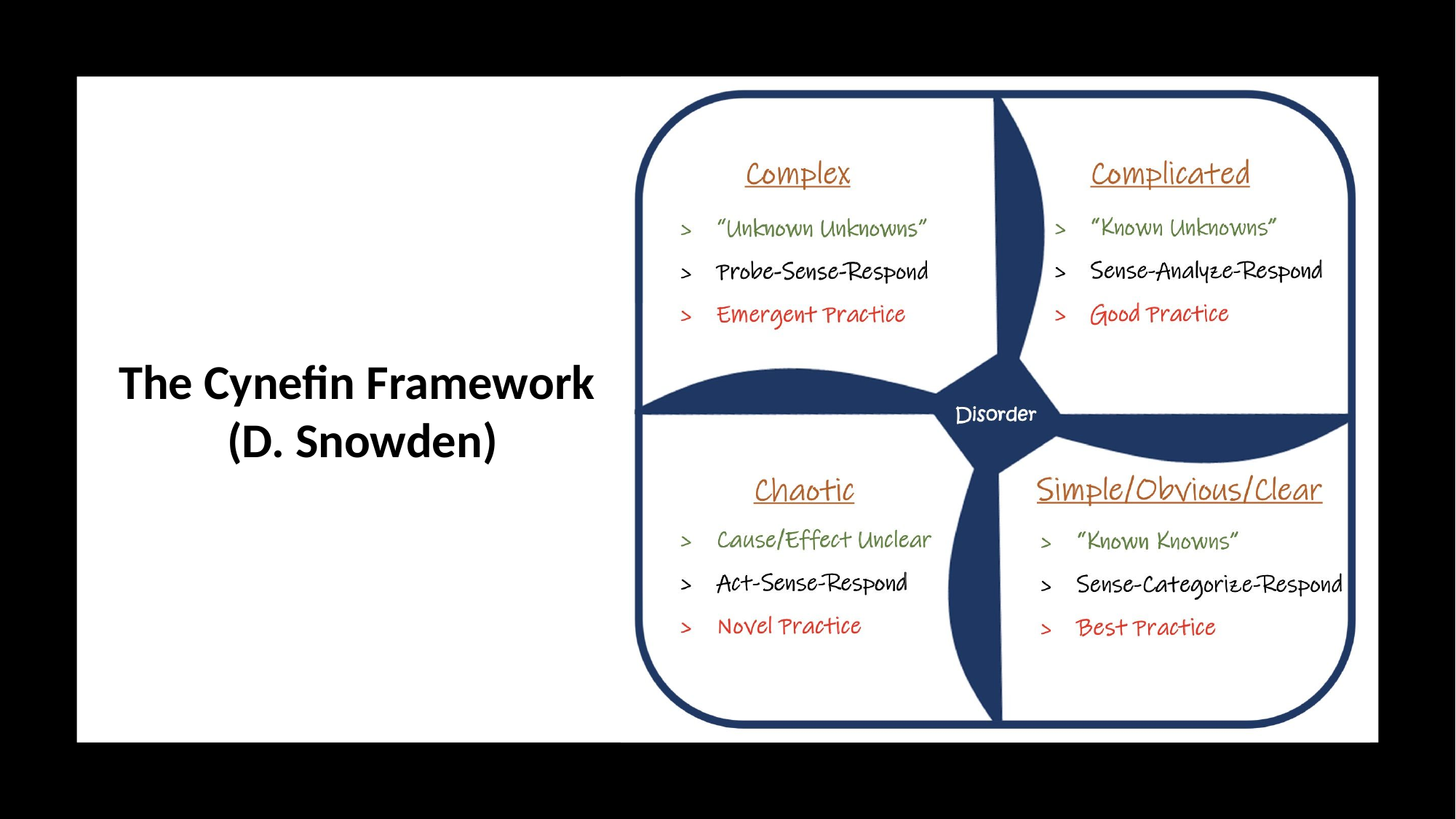

The Cynefin Framework
(D. Snowden)

## Slide 18
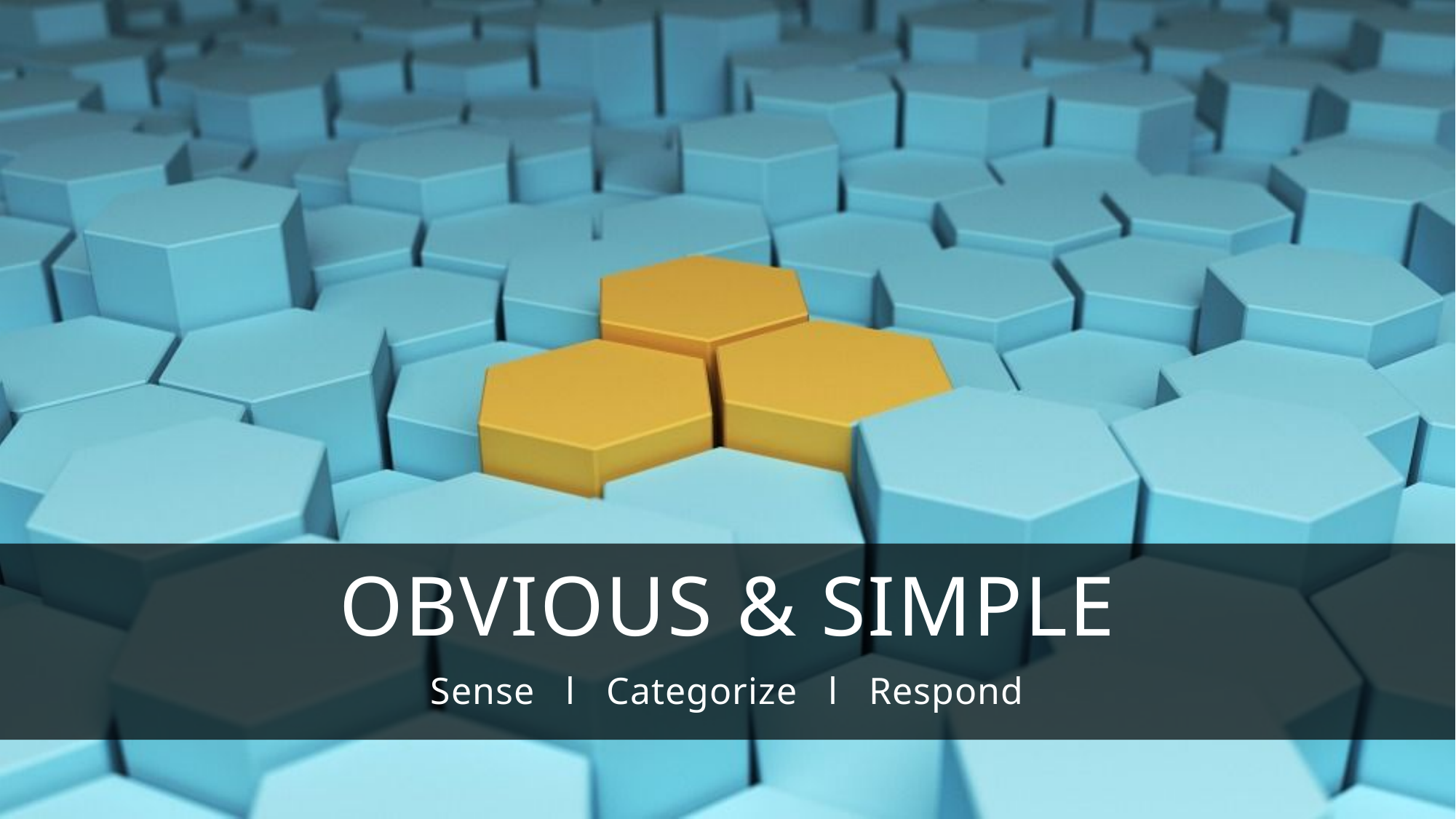

# Obvious & SIMPLE
Sense l Categorize l Respond

## Slide 19
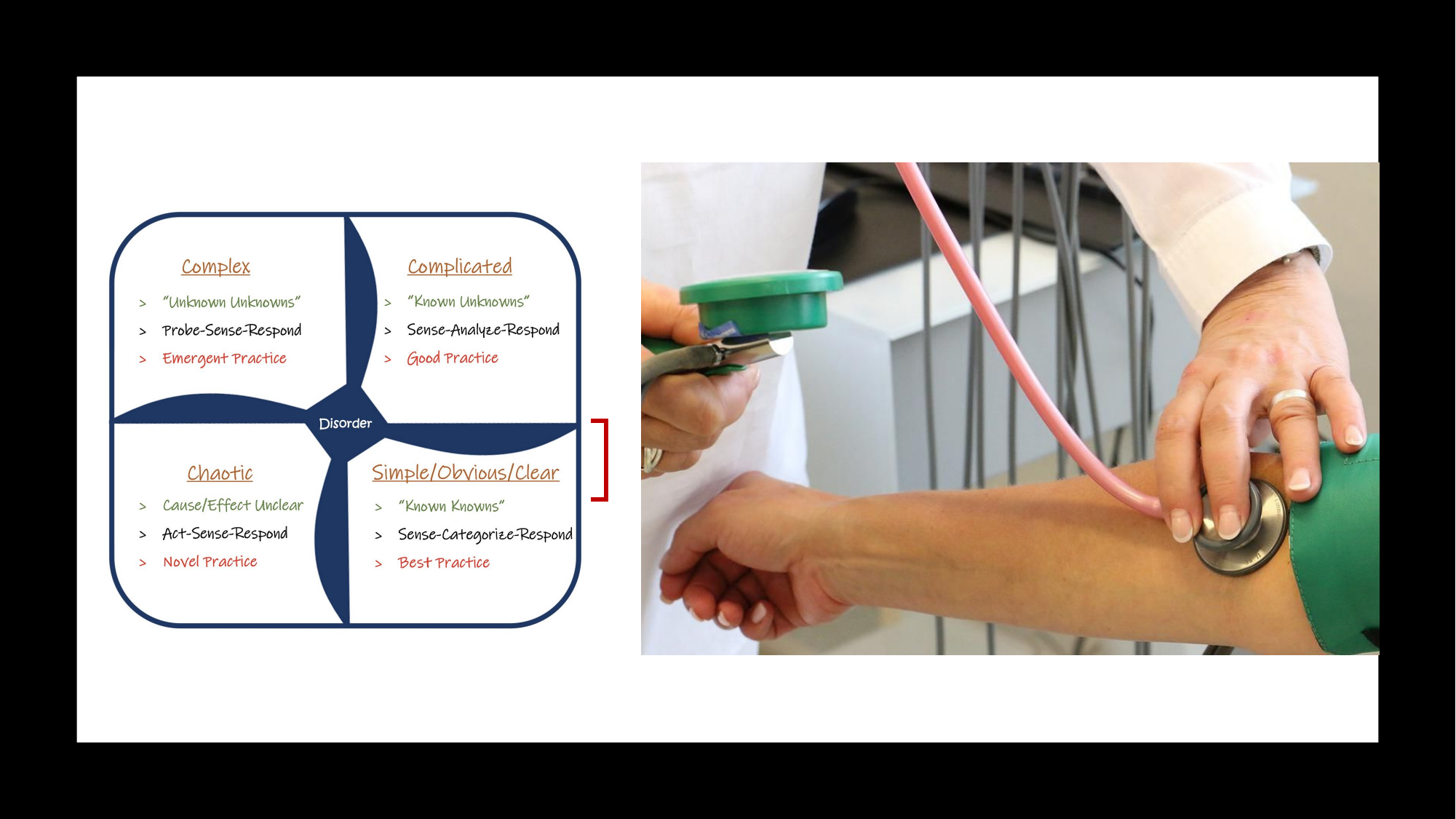

## Slide 20
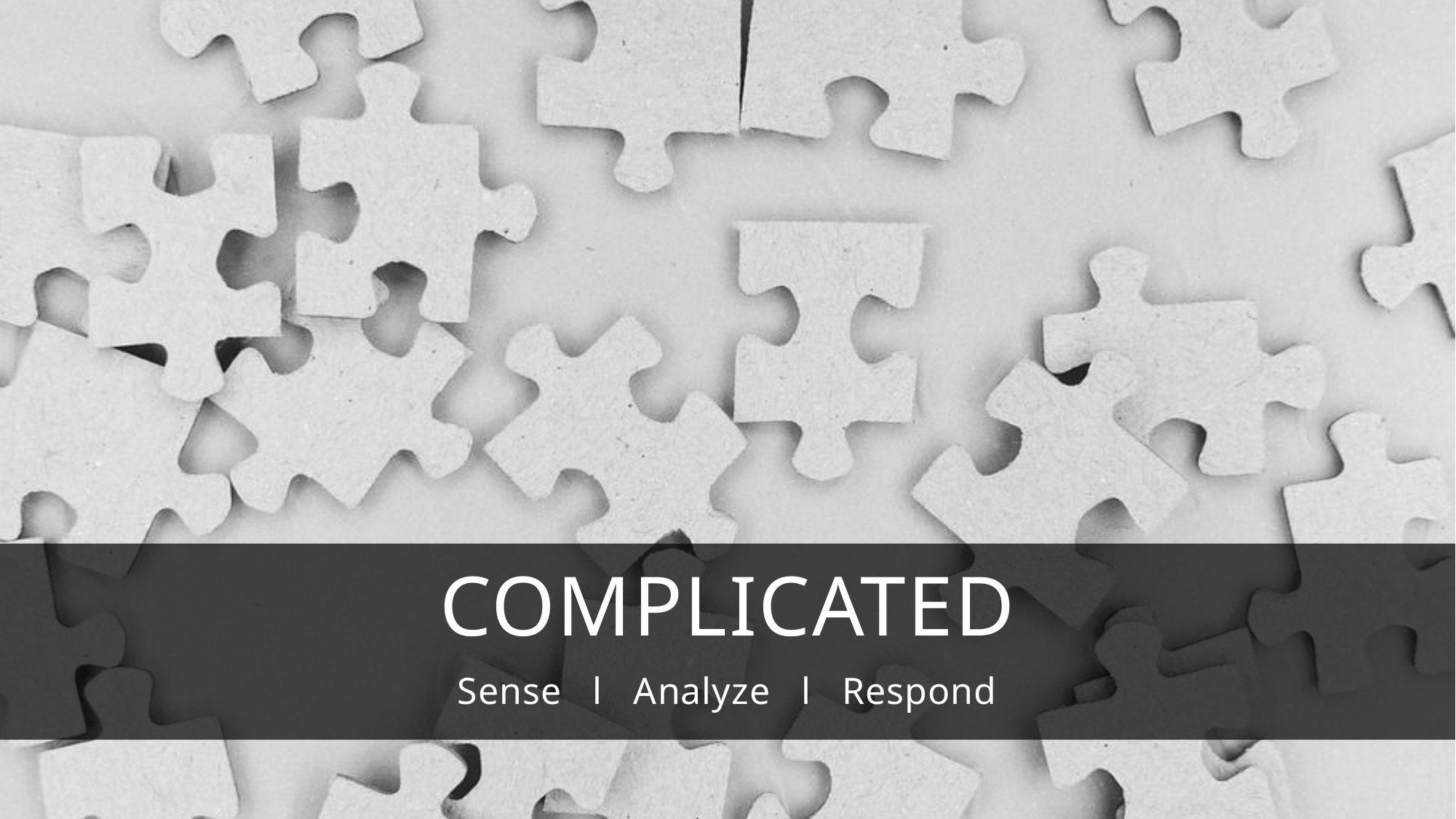

# Complicated
Sense l Analyze l Respond

## Slide 21
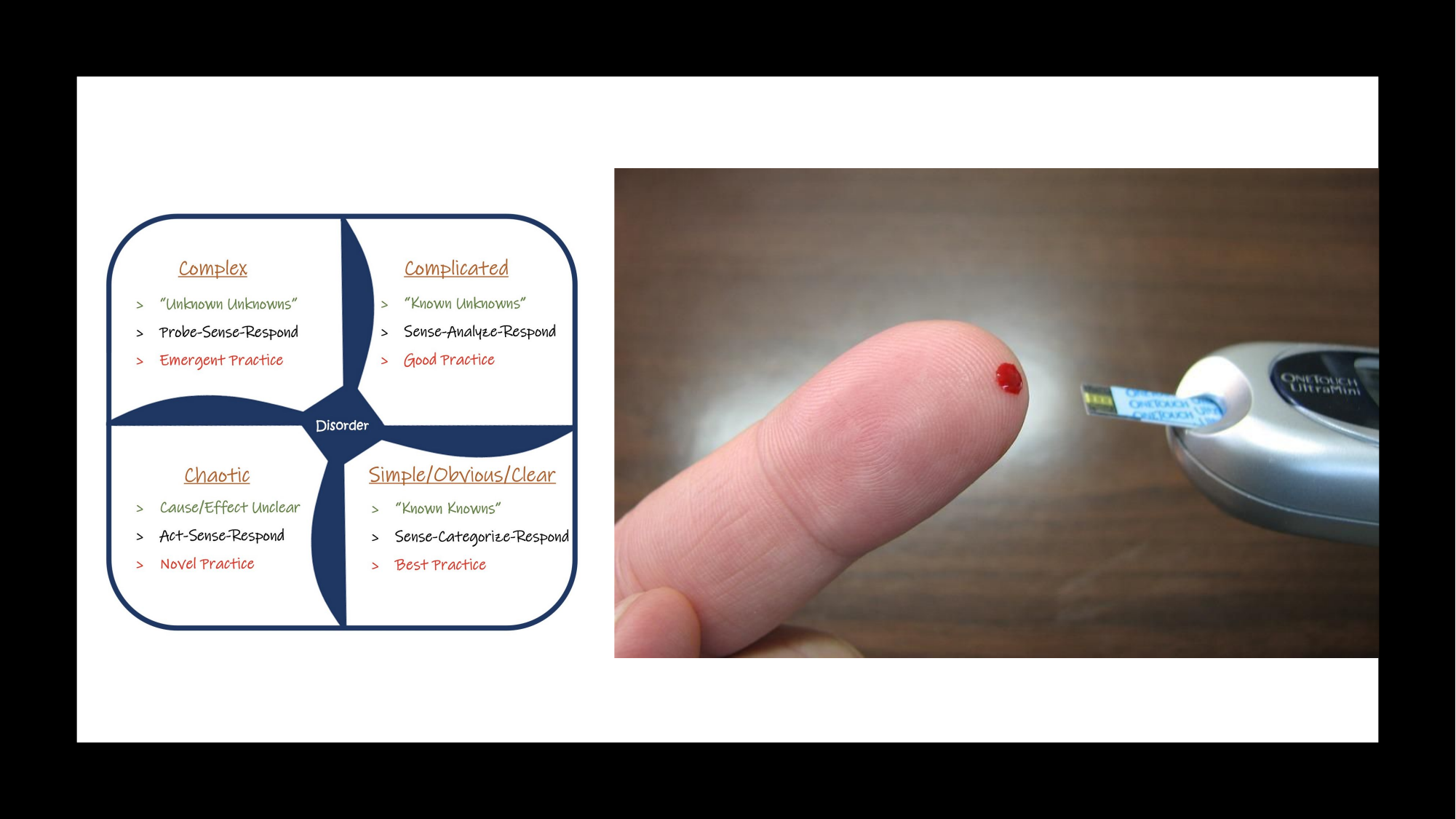

## Slide 22
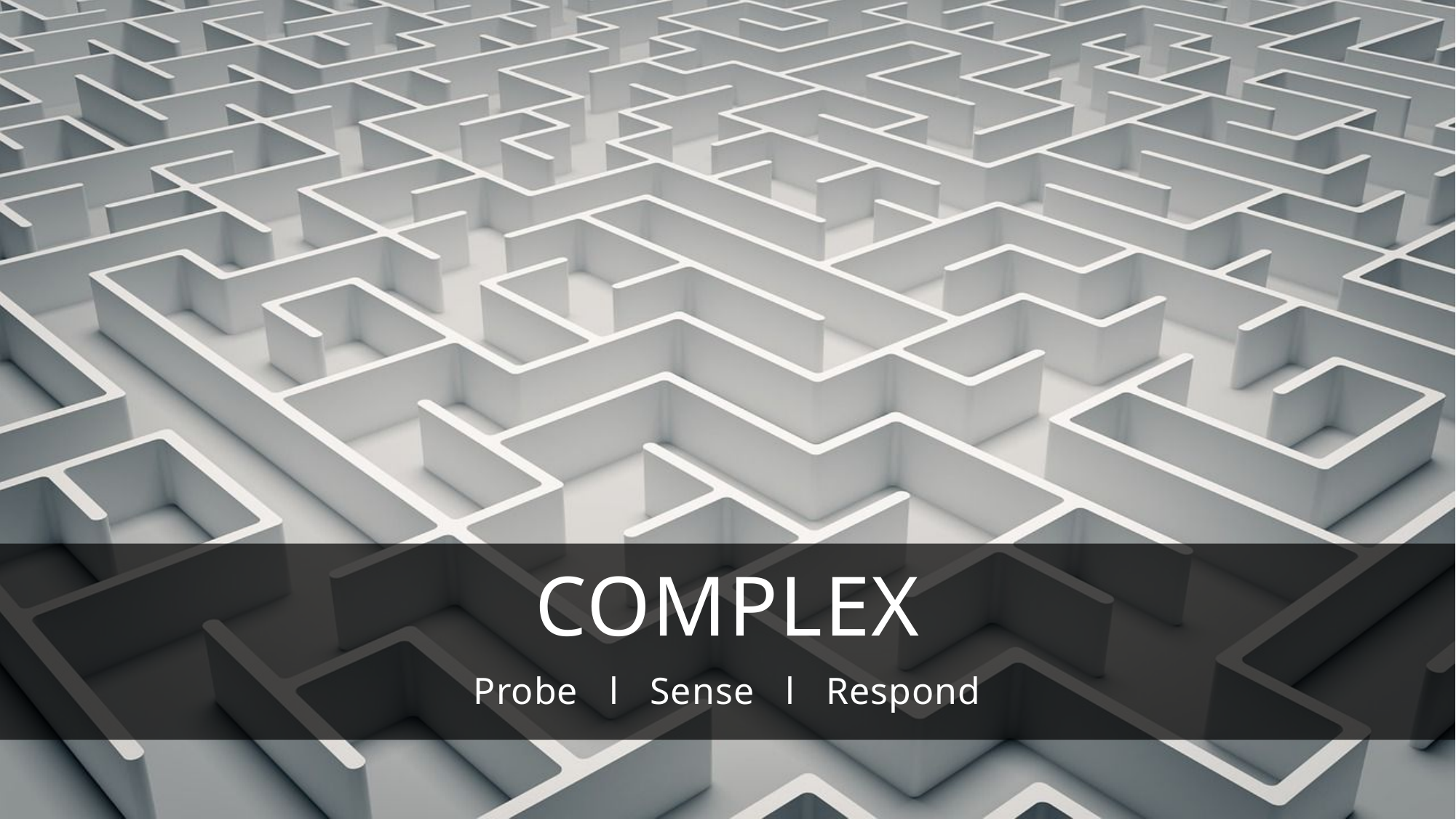

# COMPLEX
Probe l Sense l Respond

## Slide 23
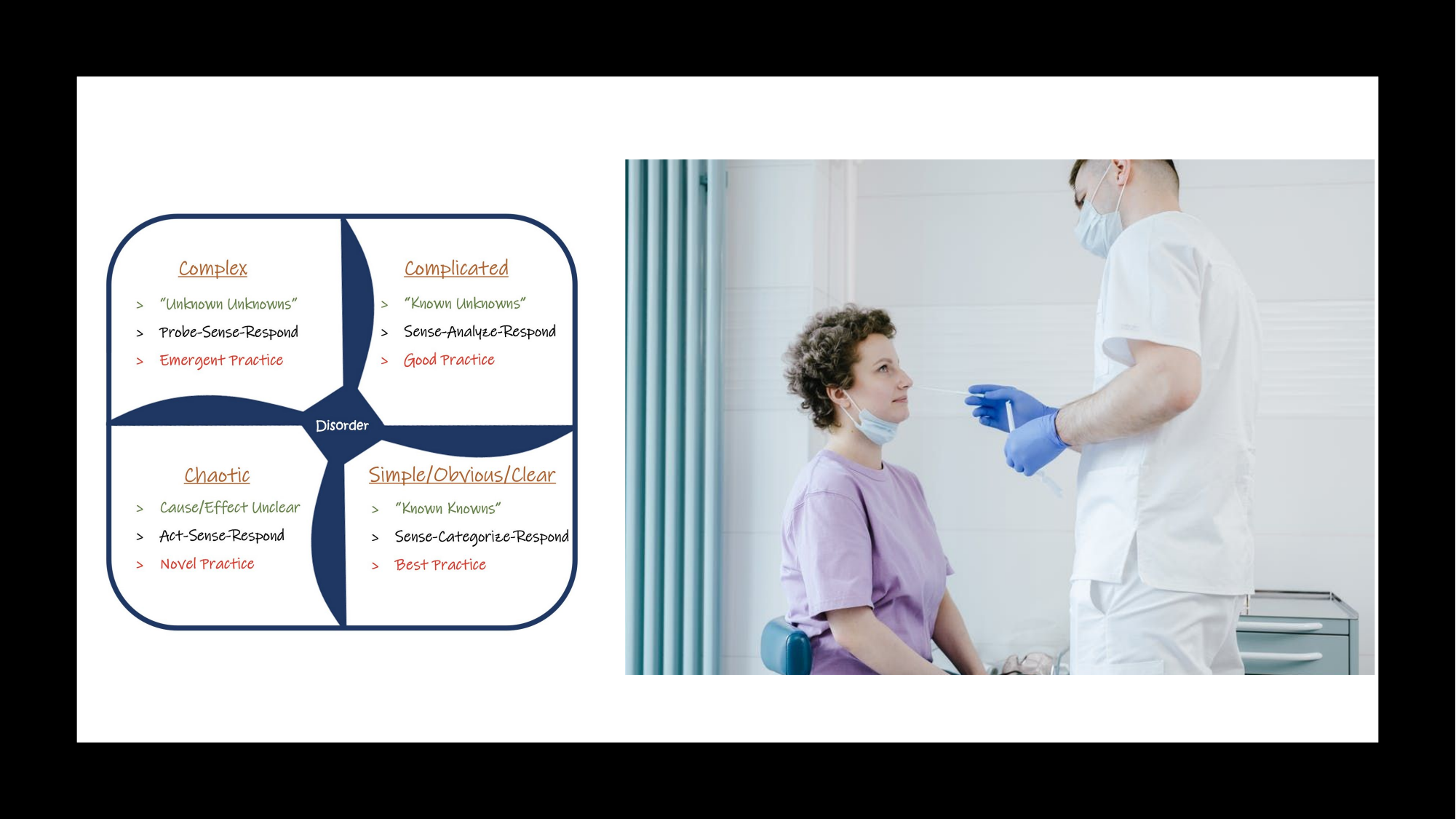

## Slide 24
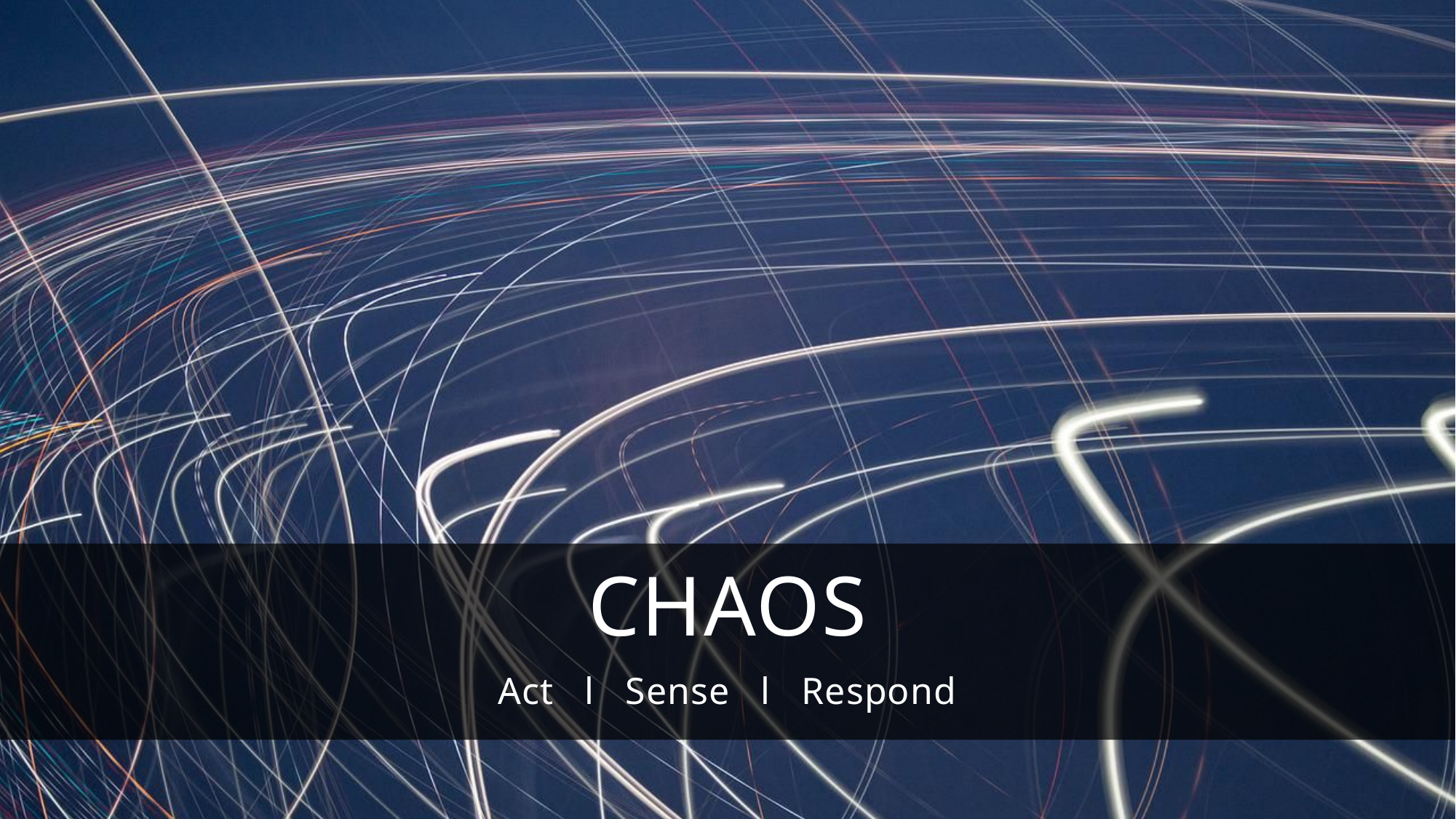

# Chaos
Act l Sense l Respond

## Slide 25
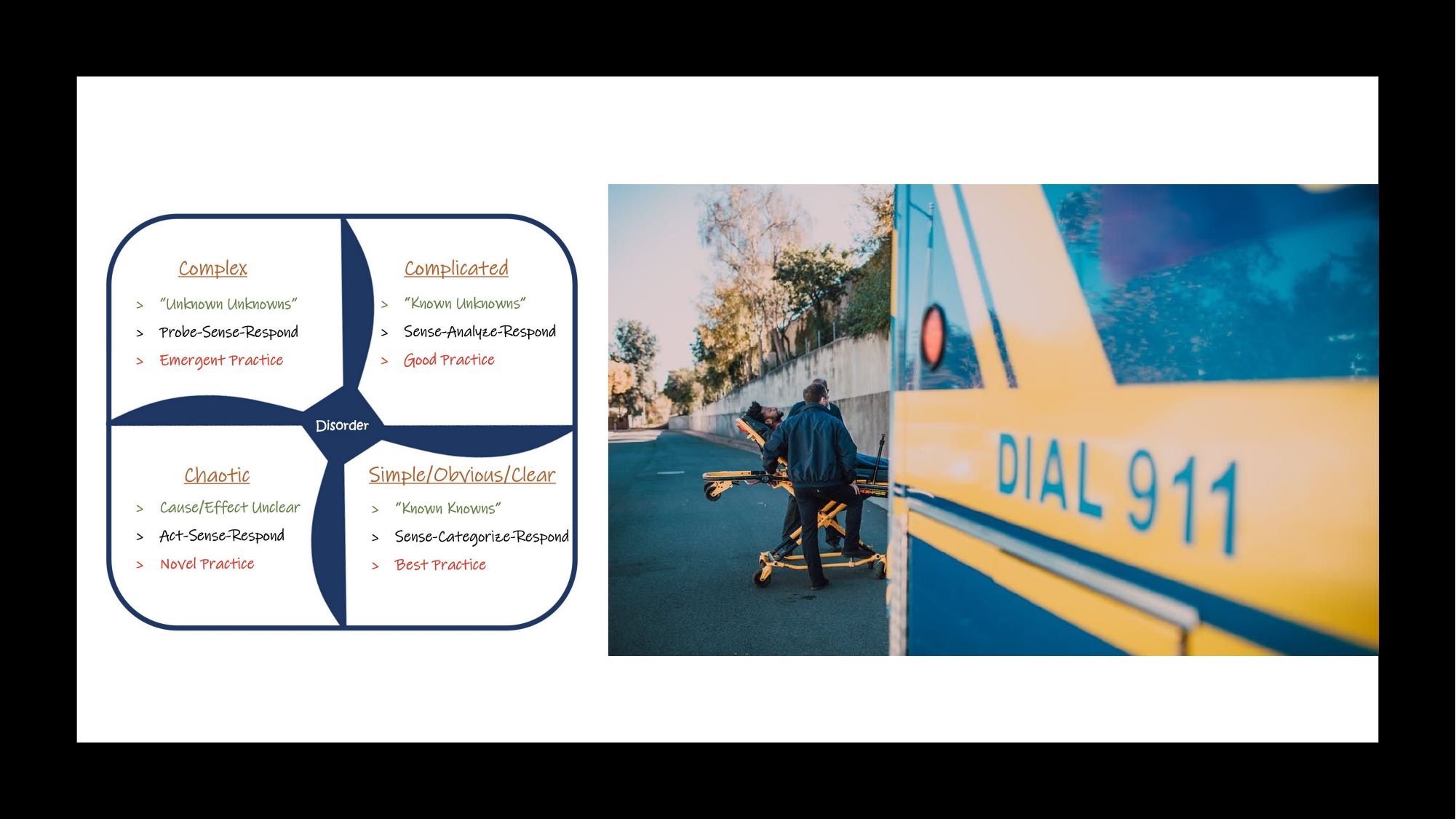

## Slide 26
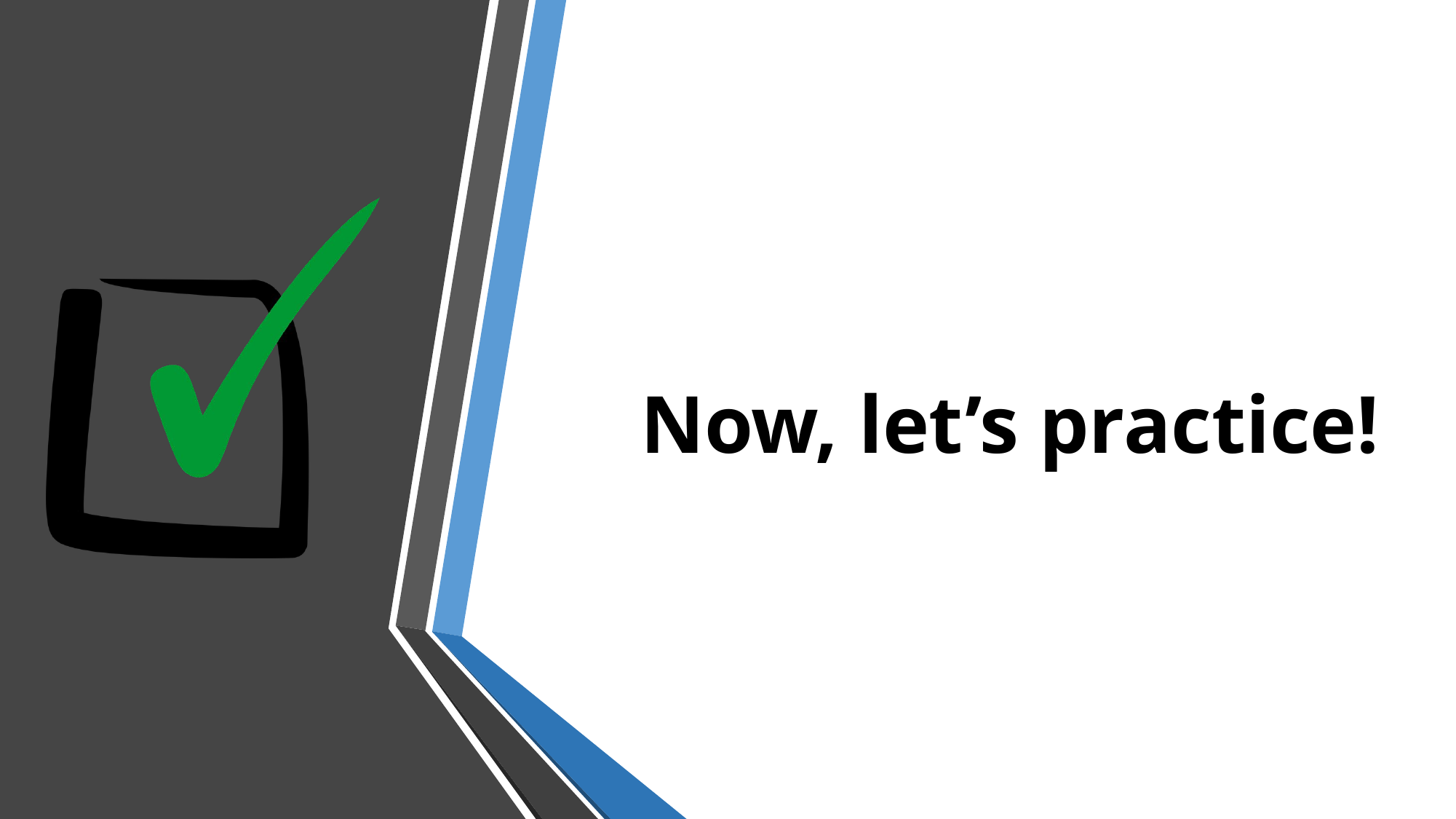

# Now, let’s practice!
