# Supplementary material for: Navigating Uncertainty in Clinical Practice: A Workshop to Prepare Medical Students to Problem-Solve During Complex Clinical Challenges
Source: MedEdPORTAL. 2023 Aug 9;19:11334. doi: 10.15766/mep_2374-8265.11334 (PMC10409886; doi:10.15766/mep_2374-8265.11334)
Supplement: Supplementary file 1 — Case Slides.pptxStudent Instructions.docxUncertainty Didactic Slides.pptxFacilitator Instructions.docxPostsession Survey.docx [file mep_2374-8265.11334-s001.zip › D. Facilitator Instructions.docx]

**Facilitator Instructions**

Navigating Uncertainty in Clinical Practice: A Workshop to Prepare Medical Students to Problem-Solve During Complex Clinical Challenges

**Sample Timeline:**

- Didactic Slides: 20 min
- Introduction to the Case Activity: 5 mins
  - Create breakout Zoom rooms during this segment (can determine based on class size)
- Case Activity: 8 challenges x 5 mins: 40 mins
  - Provide 2-minute warning for challenges – broadcast through Zoom
  - Provide 1-minute warning for challenges – broadcast through Zoom
  - Review responses and save any to highlight during the debrief
- Debrief: < 15 mins
  - 2 mins per challenge conversation
  - Highlight any exemplar strategies to share with larger group

**Didactic Instructions:**

Students will be primed for the interactive small group activity after the didactic portion of the presentation. These are located in Appendix C.

**Case Activity Instructions:**

The case activity will follow a longitudinal patient encounter during which the students will face 8 critical decision challenges. After surveying available tools to educators, the authors found Nearpod to be an easy tool to allow both slide-sharing as well as activity integration for our large class. Nearpod is a customizable, interactive virtual lesson plan software. It allows students to submit answers that can be reviewed by faculty in real time. Any similar platform may be used. Alternatively, slides can be used and answers can be submitted in other ways, such as through chat boxes, email, Google docs, etc.

In order to first create the case through Nearpod, an account should be created and provided slides can then be uploaded: <https://nearpod.com>. After accessing this link, first log in. Faculty may choose to create a free account with limited capability or choose to purchase an advanced account. Once logged in, go to “Create,” then “Create Lesson,” and then “Add Content & Activities.” In this context, items from Appendix A can be uploaded which contains the case slides. Once uploaded, select “Activities.” Here, faculty will be prompted to choose from various activities that allow them to engage with the learners through Nearpod. For this case, the authors found it best to insert “Open-ended Questions” after each challenge point.

Students should be provided a code to access the Nearpod presentation prior to being placed into breakout rooms. This code can be generated by the facilitators in Nearpod. Students should also receive a link or copy of the *Student Instructions* (Appendix B). This includes:

1. A diagram of the Cynefin Framework
2. A brief overview of the case
3. The activity instructions

Students will then be placed in a virtual breakout group (10 students per group). During this time, they will analyze the Nearpod case and the uncertainty it presents. The group should assign one student to screenshare the activity so that all breakout room participants can follow along. Another student should be assigned to enter the group’s responses to the challenge questions posed.

Students should be encouraged to reflect on the skills they have accrued thus far during their medical school training to solve clinical problems. They should apply these skills to address the patient care challenges presented to them. While they have been presented with a tool to appraise and help make sense of the uncertainty they will work through, they should know there is no correct answer. Students should be encouraged to authentically describe what they would do to solve the patient challenges. It should be reiterated that this is not necessarily about the direct medical management of the case, but rather how they navigate the uncertainty to arrive to the best course of action.

Students will have 5 minutes to appraise the uncertainty at each challenge point. They will then answer the same three questions for each challenge point:

1. Applying the Cynefin framework, appraise the uncertainty you’re working through. What quadrant are you working in? Try to make sense of why the situation is uncertain to you.
2. Classify the medical content domain of the uncertainty (as it pertains to diagnosis, prognosis, treatment, etc.).
3. Discuss how you would problem solve in this situation. What resources would you seek out? What communication strategies would you use?

To help keep time, facilitators should broadcast a 2-minute and 1-minute warning through Zoom for each challenge.

Facilitators will need to review responses to each challenge point as they are submitted, taking note of any answers to highlight or expand upon during the debrief.

**Debriefing Instructions:**

Following the interactive case portion, students will return to a main virtual room and a facilitator will debrief the scenario. The facilitator will provide additional information on how the case unfolded and will highlight any noteworthy strategies submitted by the students. They may use the following question prompts to scaffold further discussion:

- What did you observe within your group when navigating uncertainty that you feel was particularly affirming or helpful?
- What are some tips or strategies you can share with the rest of your class for dealing with uncertainty in the future?
- How did applying the Cynefin framework help your team navigate uncertainty?
- At what moment in the case experience did you feel most engaged with what was happening?
- At what moment in the case experience were you most distanced from what was happening?
- What about the workshop surprised you the most (this could be your own reactions to what went on, something you learned, or something that someone else contributed)?

**Content Expert Consensus on Challenge Point Answers:**

This information is also included in the notes section of the Case Slides PowerPoint (Appendix A).

**Challenge #1:**

1. **Quadrant:** Complicated; multiple correct answers that rely on good practices and prompt the learner to analyze elements of the patient presentation
2. **Classify The Uncertainty:** Diagnosis, Treatments, Processes of Care, Psychosocial
3. **Problem-solving Strategies/How the Case Unfolded:** Challenge #1 focused on meeting the patient in the ED. The good thing about being a medical student is that you usually have a little more time than the other providers to listen to and reflect on the patients you are seeing. The patient is here for evaluation of their ankle but you've identified a bigger problem. Something is happening beyond a typical presentation of an ankle sprain, and it is likely it is unrelated. This is certainly not normal for someone with Down Syndrome either. You're going to encounter this when you advocate for patients and need to deviate from a specific plan. This can feel uncomfortable. What you can do as the ER physician is to ask additional questions, loop in other services if needed, and establish appropriate follow-up care, maybe beyond that of their usual PCP. It is possible to end up in a complex category where you have to push boundaries. In this situation, the physician arranged for the patient and her family to meet with a primary physician who specializes in patients with Down Syndrome.

**Challenge #2:**

1. **Quadrant:** Simple; there are best practices in communication the learner can consult to articulate these findings to the patient and family
2. **Classify The Uncertainty:** Diagnosis, Causality, Structures of Care
3. **Problem-solving Strategies/How the Case Unfolded:** The new primary physician evaluated some new labs for the patient and diagnosed her with hypothyroidism. The presentation of hypothyroidism was not straightforward, but if you listened to the patient's complaints over the past few months, you could see that the diagnosis was there. This was a case of central hypothyroidism with secondary adrenal insufficiency. Additional diagnostic workup provided this clarity. The new primary doctor had to give these results and had to inform the patient and family that the prior information was not correct. The focus was forward thinking and together they focused on the next steps.

**Challenge #3:**

1. **Quadrant:** Simple/Complex; while there are best practices the learner can adhere to, some of these may be complicated by the circumstances of the patient’s condition. Both best and good practices can be considered
2. **Classify The Uncertainty:** Diagnosis, Treatment
3. **Problem-solving Strategies/How the Case Unfolded:** At this point, the patient requires an MRI and the physician needs to address his ability to tolerate this imaging study. It was important for the physician to emphasize the need for a further workup to look for a pituitary mass. Most of these cases are benign, but you have to get additional imaging to determine this. The physician focused on the patient's concerns - how do you do with loud noises, with lying still for an hour? They discussed other options and decided to try the MRI and if that didn't work, then they would proceed with some sedation for the MRI. The patient tolerated the study by listening to her favorite soundtrack.

**Challenge #4:**

1. **Quadrant:** Complicated; multiple correct answers that rely on good practices and prompt the learner to analyze elements of the patient presentation
2. **Classify The Uncertainty:** Diagnosis, Prognosis, Psychosocial, Processes of Care
3. **Problem-solving Strategies/How the Case Unfolded:** A few days later, the physician received a note on a Friday afternoon about a concerning pituitary mass that is pushing on the optic chiasm. The physician starts to piece together additional complaints the patient had, such as her blurred vision. The physician has to navigate this complex situation in which there are multiple approaches for gathering more information and discussing this with the patient and family.  The MRI did not indicate an immediate threat and so the physician decided it was better to take the weekend to do some homework. He realized it can be difficult to start a conversation and then leave the patient and family in the dark. Instead, he sought out additional resources by reaching out to his neurosurgery colleague to help determine the rest of the treatment plan. The family was called on Monday to schedule an appointment that same day to review the test results, indicating that they want to have some time to answer all of their questions. In order to make the most of the meeting, the medical team asked for the parents and sister to come, knowing that the sister played a big part in the decisions. They established beforehand if they would like a translator present or not.

**Challenge #5:**

1. **Quadrant:** Complicated; multiple correct answers that rely on good practices and prompt the learner to analyze elements of the patient presentation
2. **Classify The Uncertainty:** Diagnosis, Prognosis, Psychosocial, Processes of Care
3. **Problem-solving Strategies/How the Case Unfolded:** The physician rehearsed what he was going to say and played out the different possible scenarios in order to be as prepared as possible. The physician planned to focus on the gravity of the news, but emphasize his continued support.

**Challenge Point 6:**

1. **Quadrant:** Complicated; multiple correct answers that rely on good practices and prompt the learner to analyze elements of the patient presentation
2. **Classify The Uncertainty:** Prognosis, Existential, Causality
3. **Problem-solving Strategies/How the Case Unfolded:** Part of the consent should involve some of these discussions so it should not be unexpected when the surgeon tells the patient and family that things did not go as planned. The patient suffered no harm from the procedure but the surgeon understands there will be more time and additional workup. The surgeon communicated this with the primary physician as well so everyone was on board.

**Challenge Point 7:**

1. **Quadrant:** Complicated; multiple correct answers that rely on good practices and prompt the learner to analyze elements of the patient presentation
2. **Classify The Uncertainty:** Diagnosis, Prognosis, Treatment, Processes of Care
3. **Problem-solving Strategies/How the Case Unfolded:** The results showed it was not a malignant tumor but the location was concerning. In order to communicate this best with the patients, the surgeon arranged an appointment with the patient and her family, similar to how the primary doctor did. He communicated the results to the sister who then explained it to the patient. In order to involve the parents as well, the surgeon used a translator service. The surgeon used clear communication and showed a lot of compassion toward everyone. The surgeon and primary physician also communicated thoroughly so that the primary doctor could also follow up with the family and help to answer any additional questions. When dealing with the uncertainty in this situation, the physician and surgeon relied on each other a lot to come up with the management plan and communicate this to the patient and family. They were transparent with each other and with the patient.

**Challenge Point 8:**

1. **Quadrant:** Complex; multiple unknowns to consider in the outpatient settings. Social and structural components complicate the management plan.
2. **Classify The Uncertainty:** Treatment, Structures of care, Psychosocial
3. **Problem-solving Strategies/How the Case Unfolded:** In this last challenge point, the patient is ready to be discharged home in this challenging condition. There was a complexity to the follow-up care, but this was navigated by focusing on the main points with the patient and family which were to continue the prednisone and Keppra. Having established a good patient-doctor relationship, the physician was comfortable that the patient and her family would be comfortable reaching out if they had additional questions and they reviewed the various ways to get in touch should they need to at any time and no matter how often.
