# Supplementary material for: Navigating Uncertainty in Clinical Practice: A Workshop to Prepare Medical Students to Problem-Solve During Complex Clinical Challenges
Source: MedEdPORTAL. 2023 Aug 9;19:11334. doi: 10.15766/mep_2374-8265.11334 (PMC10409886; doi:10.15766/mep_2374-8265.11334)
Supplement: Supplementary file 1 — Case Slides.pptxStudent Instructions.docxUncertainty Didactic Slides.pptxFacilitator Instructions.docxPostsession Survey.docx [file mep_2374-8265.11334-s001.zip › E. Postsession Survey.docx]

**Post-Session Survey**

**Q1 - Do you feel your perspectives surrounding uncertainty in clinical practice have changed after this session?**

Yes

No

**Q2 - Please describe how your perspectives on uncertainty in clinical practice have changed.**

**Q3 - Uncertainty is frequently encountered at various points in clinical practice. Which of the following do you feel LEAST prepared for when navigating uncertainty in clinical practice? Please select all that apply.**

Asking for help during times of uncertainty

Communicating with patients during times of uncertainty

Decision-making during times of uncertainty

Diagnostic uncertainty

Eliciting patient preferences during times of uncertainty

Prognostic uncertainty

Uncertainty when selecting a treatment plan

Other (please describe)

**Q5 - If “other”, please describe.**

**Q6 - How useful was today's workshop in preparing you to problem-solve during instances of uncertainty in clinical practice?**

Not at all useful

Slightly useful

Moderately useful

Very useful

Extremely useful

**Q7 - Did you find the Cynefin framework to be a helpful tool when appraising the uncertainty in clinical practice?**

Yes

No

**Q8 - Please share why you found the Cynefin framework to be helpful when appraising the uncertainty you are working through during clinical practice.**

**Q9 - Please share why you DID NOT find the Cynefin framework to be helpful when appraising the uncertainty you are working through during clinical practice.**

**Q10 - This is the first year we have included this workshop in the Gateway curriculum. Should this workshop be included in the Gateway curriculum in the future?**

Yes

No

**Q11 - Please share one strategy that was discussed today you will apply when problem-solving through uncertainty in the clinical environment.**

**Q12 - Please offer any suggestions to improve this workshop in the future.**
